# Iridoid glycoside dimers from fruits of *Cornus officinalis* and their anti-inflammatory activity

**DOI:** 10.3389/fchem.2025.1558075

**Published:** 2025-03-17

**Authors:** Ying-Chu Shi, Yu-Xin Yu, Jiu-Xia Gao, Xin Wang, Xiao-Ya Shang, Jia Xu

**Affiliations:** ^1^ Beijing University of Chinese Medicine, Beijing, China; ^2^ Beijing Hospital of Traditional Chinese Medicine, Capital Medical University, Beijing, China; ^3^ College of Applied Arts and Science, Beijing Union University, Beijing, China

**Keywords:** *Cornus officinalis*, iridoid glycoside dimers, isolation, structure identification, anti-inflammatory activity

## Abstract

A bioassay-guided phytochemical study of the fruits of *Cornus officinalis* led to the isolation of six new iridoid glycoside dimers, named corndiridoside A-F (**1–6**), along with 11 analogs (**7–17**). The structure of these dimers was elucidated using HRESIMS, 1D and 2D NMR, IR, and UV spectra, as well as literature comparisons. The anti-inflammatory activity of all compounds was evaluated, revealing a significant inhibitory effect on all dimers on the production of NO in LPS-stimulated RAW 264.7 cells at concentrations of 25 and 50 μM. Of the six, compounds **2** and **3** showed the strongest anti-inflammatory activity.

## 1 Introduction

Inflammation is a defensive response of host tissues to injuries, external pathogens, and foreign bodies; long-term inflammatory responses and persistent chronic inflammation can damage the body’s homeostasis (Kotas and Medzhitov, 2015). Inflammatory responses include both acute and chronic inflammation. Chronic inflammation is associated with diseases such as diabetes, obesity, cancer, atherosclerosis, neurological diseases, and atopic dermatitis (Headland and Norling, 2015; Germolec et al., 2018). Natural products with anti-inflammatory activity and extracted from plants play an important role in the development of anti-inflammatory drugs or functional foods.

*Cornus officinalis* plant belongs to the Cornaceae family and is mainly found in East Asia, including China, Korea, and Japan. Its dried mature fruits are widely used both as traditional Chinese medicine as well as healthy edible food and have anti-inflammatory, antioxidation, neuroprotective, and hypoglycemic effects (Huang et al., 2018; Gao et al., 2021). The extract of *C. officinalis* has shown significant anti-inflammatory activity in several studies (Sung et al., 2009; Quah et al., 2020; Yang et al., 2024). For example, Quanh et al. (2020) reported that the ethanol extract of *C. officinalis* had a potential therapeutic effect on atopic dermatitis by inhibiting iNOS mRNA expression, and pro-inflammatory cytokines (IL-1β, IL-6, and TNF-α) and LPS-induced nitric oxide (NO) production in RWA 264.7 cells (Quah et al., 2020). Iridoid glycosides, gallate derivatives, and triterpenoids are considered to be anti-inflammatory components of *C. officinalis* (Jang et al., 2014; Yuan et al., 2020; Li et al., 2021). Of these components, iridoid glycosides, including monomers and dimers, are the main anti-inflammatory active ingredients in *C. officinalis*. Total cornel iridoid glycoside and some iridoid glycosides including morroniside, loganin, cornuside, and iridoid dimers have been reported to show significant anti-inflammatory activity by regulating different inflammatory factors (Ye et al., 2017; Park et al., 2021; Peng et al., 2022; Wang et al., 2022; Zheng et al., 2022; Tong et al., 2023; Yan et al., 2024). Although some iridoid glycosides with anti-inflammatory activity have been extracted from *C. officinalis*, their anti-inflammatory components remain poorly understood. To further elucidate the anti-inflammatory activity of iridoid glycosides, based on an *in vitro* anti-inflammatory activity test, the 50% ethanol aqueous extract of *C. officinalis* was chromatographically isolated to obtain the iridoid glycoside enrichment site with the strongest anti-inflammatory activity. Herein, six new iridoid glycoside dimers (**1–6**) and 11 (**7–17**) analogs were isolated from the strongest anti-inflammatory fraction, and their isolated procedures, and structural elucidation were reported in the present study. Additionally, the *in vitro* anti-inflammatory activity of the isolated compounds was measured on the LPS-stimulated RAW 264.7 cells model.

## 2 Materials and methods

### 2.1 General Experimental Procedures

Nuclear magnetic resonance (NMR) spectroscopy of isolated compounds (CD_3_OD) was acquired on a Bruker AV-III-500 (500 MHz for ^1^H and 125 MHz for ^13^C) spectrometers (Bruker, Billerica, MA, United States). High-resolution electrospray ionization mass spectroscopy (HRESIMS) data were obtained using a Thermo QE Plus spectrometer (Thermo Scientific, Waltham, MA, United States). Optical rotation data were recorded using a Rudolph Research Autopol III automatic polar spectrometer (Rudolph Research Analytical, Zurich, Switzerland). Infrared (IR) spectra were recorded on a Nicolet Impact 400 FT-IR spectrophotometer (Nicolet, Madison, WI, United States). Furthermore, column chromatographical separation was performed on Diaion HP-20 macroporous resin (Mitsubishi Chemical Corporation, Tokyo, Japan), Sephadex LH-20 gel (Pharmacia Biotech AB, Uppsala, Sweden), silica gel (Qingdao Marin Chemical Inc., Qingdao, China). A Waters HPLC (Waters 2,545 controller with a Waters 2,998 dual-wavelength absorbance detector) with a Waters preparative Rp C_18_ chromatographic column (X-bridge, 250 mm × 19 mm, 5 μm) was employed for HPLC preparative (Waters, Milford, MA, United States). The chemical reagents, including analytical grade and chromatographic grade, were purchased from Tianjin Fuyu (Tianjin, China). The RAW 264.7 cells (No. 1101MOU-PUMC000146) used in this study were obtained from the National Infrastructure of Cell Line Resource (Peking Union Medical College, Beijing, China).

### 2.2 Plant material

The matured and dried fruits of *C. officinalis* were obtained in June 2023 from the Beijing Hospital of Traditional Chinese Medicine (Beijing, China), and authenticated by Pro. Sheng Lin (Beijing University of Traditional Chinese Medicine). A voucher specimen (No. 20230101) has been deposited at the Department of Dermatology, Beijing Hospital of Traditional Chinese Medicine, Beijing, China.

### 2.3 Isolation and purification

The air-dried fruits (10 kg) of *C. officinalis* were powdered and extracted with 50% EtOH aqueous (100 L × 3) by ultrasound at room temperature. After filtration, the extract was evaporated under reduced pressure to obtain crude extract. After suspending into H_2_O, a Diaion HP-20 Macroporous Resin column was used to separate the extract with EtOH-H_2_O (0:100, 20:80, 40:60, 70:30, and 95:5, *v/v*), yielding five fractions (Fr.A –Fr. E). The EtOH-H_2_O (40:60) fraction (Fr. C) was further subjected to a silica gel chromatography eluting with CHCl_3_-MeOH (15:1 – 8:1, *v/v*) to afford six major fractions (Fr.C1 – Fr.C6). The anti-inflammatory activity test exhibited that Fr.C4 had the strongest inhibitory activity; therefore, it was selected as the target fraction for further separation. Fraction Fr.C4 was subjected to a reversed-phase C_18_ silica gel with MeOH-H_2_O (10:90 – 100:0, *v/v*) to obtain seven faction Fr.C4-1 ∼ Fr.C4-7. Subfraction Fr.C4-2 was further separated by a reversed-phase C_18_ silica gel eluting with MeOH-H_2_O (20:80 ∼ 100:0, *v/v*) to yield five fractions Fr.C4-2-1∼ Fr.C4-2-5. Subfraction Fr.C4-2-2 was separated by a Sephadex LH-20 gel column (CHCl_3_-MeOH 2:1, *v/v*) and further purified by preparative HPLC using MeCN-H_2_O (20:80, 18 mL/min) to yield compounds **7**, **9**, and **12**. Subfraction Fr.C4-2-3 was subjected to a Sephadex LH-20 gel column eluting with CHCl_3_-MeOH (2:1, *v/v*) and further purified by preparative HPLC eluting with MeOH-H_2_O (40:60, 18 mL/min) to yield **4**, **14** and **8**. Fr.C4-2-4 was purified using preparative HPLC eluting with MeCN-H_2_O (20:80, 18 mL/min) to yield compounds **5** and **6**. Subfraction Fr.C4-2-5 was given to a Sephadex LH-20 gel column eluting with CHCl_3_-MeOH (2:1, *v/v*) and further purified by using preparative HPLC (20% MeCN/H_2_O, 18 mL/min) to yield **1**, **10** and **11**. Fr.C4-3 was purified on a Sephadex LH-20 gel column with CHCl_3_-MeOH (2:1, *v/v*) elution and further prepared by HPLC (20% MeCN/H_2_O, 18 mL/min) to yield compounds **13** and **15**. Fr.C4-4 was given to HPLC using 40% MeOH/H_2_O (18 mL/min) to yield **16** and **17**. Fr.C4-6 was separated on a Sephadex LH-20 gel column with CHCl_3_-MeOH (2:1, *v/v*) and further purified by using preparative HPLC (25% MeCN/H_2_O, 18 mL/min) to yield compounds **2** and **3**.

Corndiridoside A (**1**): White amorphous powder; [*α*] −45.6 (0.03, MeOH); UV (MeOH) λ_max_ (log ε) 239 (3.95) nm; IR v_max_ 3,401, 2,916, 1703, 1,636, 1,077 cm^−1^; ^1^H NMR (500 MHz, CD_3_OD) and ^13^C NMR (125 MHz, CD_3_OD) ( see Table 1); HRESIMS *m/z* 775.26660 [M−H]^−^ (calculated for C_34_H_47_O_20_, 775.26662).

**TABLE 1:** The ^1^H and^13^C NMR data of compounds **1–3**.

Position	1		2		3	
	*δ*_H_	*δ*_C_	*δ*_H_	*δ*_C_	*δ*_H_	*δ*_C_
1	5.66, d, *J* = 2.8 Hz	94.9	5.90, d, *J* = 9.3 Hz	95.8	5.82, d, *J* = 8.8 Hz	96.1
3	7.51, d, *J* = 1.1 Hz	153.2	7.52, s	154.5	7.55, s	154.1
4	—	111.1	—	111.7	—	110.8
5	3.29, m	28.1	3.06, m	27.8	2.81, m	31.8
6	(a) 2.55, m (b) 2.61, dd, *J* = 19.1, 11.2 Hz	43.4	(a) 1.45, m (b) 1.97, m	34.0	(a) 1.14, m (b) 1.96, m	35.7
7	—	220.8	4.92, d, *J* = 3.2 Hz	99.1	5.00, d, *J* = 3.2 Hz	102.4
8	2.09, m	44.5	4.41, m	66.5	3.92, m	74.1
9	2.34, m	46.5	1.81, m	40.3	1.84, m	40.3
10	1.15, d, *J* = 7.1 Hz	13.5	1.34, d, *J* = 6.9 Hz	19.7	1.40, d, *J* = 6.9 Hz	19.7
11	—	168.9	—	168.6	—	168.6
12	3.71, s	51.8	3.67, s	51.7	3.66, s	51.7
Glc-1′	4.75, d, *J* = 7.8 Hz	99.7	4.81, d, *J* = 7.9 Hz	100.4	4.83, *J* = 7.3 Hz	100.2
2′	3.21, m	73.2	3.34, m	75.0	3.41, m	75.2
3′	3.59, m	86.0	3.28, m	78.5	3.30, m	78.4
4′	3.38, m	70.2	3.36, m	71.6	3.38, m	72.2
5′	3.27, m	78.2	3.48, m	76.7	3.51, m	77.2
6′	(a) 3.66, dd, *J* = 5.6, 11.9 Hz (b) 3.90, dd, *J* = 1.5, 11.9, Hz	62.9	(a) 3.62, dd, *J* = 1.9, 11.2 Hz (b) 3.97, dd, *J* = 6.1, 11.2 Hz	67.6	(a) 3.6, dd, *J* = 7.5, 11.5 Hz (b) 4.03, dd, *J* = 1.7, 11.5 Hz	68.5
1″	5.88, d, *J* = 9.3 Hz	96.4	5.84, d, *J* = 9.3 Hz	95.7	5.78, d (8.8)	95.6
3″	7.52, s	154.7	7.51, s	154.6	7.43, s	154.5
4″	—	110.8	—	111.7	—	111.7
5″	2.87, m	32.1	3.06, m	27.8	3.09, m	27.8
6″	(a) 1.31, m (b) 2.25, m	35.5	(a) 1.40, m (b) 1.90, m	33.7	(a) 1.51, m (b) 1.99, m	34.0
7″	4.79, dd, *J* = 2.3, 9.6 Hz	103.3	4.87, d, *J* = 3.2 Hz	97.9	4.80, d, *J* = 3.0 Hz	99.7
8″	4.01, m	74.4	4.30, m	66.3	4.45, m	66.5
9″	1.81, m	39.8	1.81, m	40.1	1.79, m	40.0
10″	1.45, d, *J* = 6.8 Hz	19.7	1.25, d, *J* = 6.85 Hz	19.6	1.36, d, *J* = 6.9 Hz	19.7
11″	—	168.6	—	168.6	—	168.6
12″	3.70, s	51.7	3.68, s	51.7	3.70, s	51.7
Glc-1‴	4.75, d, *J* = 7.8 Hz	100.7	4.80, d, *J* = 7.9 Hz	100.0	4.80, d, *J* = 7.9 Hz	100.1
2‴	3.19, m	74.9	3.24, m	75.0	3.22, m	75.0
3‴	3.21, m	78.7	3.26, m	78.1	3.30, m	78.0
4‴	3.24, m	71.8	3.26, m	71.7	3.30, m	71.5
5‴	3.36, m	78.0	3.38, m	77.9	3.40, m	77.9
6‴	(a) 3.55, dd, *J* = 12.3, 6.7 Hz (b) 3.85, dd, *J* = 12.3, 1.9 Hz	62.5	(a) 3.67, m (b) 3.89, dd, *J* = 1.8, 11.1 Hz	62.8	(a) 3.67, m (b) 3.87, dd,*J* = 1.9, 12.2 Hz	62.8
1⁗			9.56	179.5	9.58	179.6
2⁗			—	154.2	—	154.6
3⁗			6.67, d, *J* = 3.5 Hz	124.9	6.64, d, *J* = 3.6 Hz	124.0
4⁗			7.38, d, *J* = 3.5 Hz	112.9	7.41, d, *J* = 3.6 Hz	112.7
5⁗			—	160.0	—	159.8
6⁗			4.59, d, *J* = 13.7 Hz 4.63, d, *J* = 13.7 Hz	61.9	4.64, d, *J* = 13.8 Hz 4.64, dd, *J* = 1.6, 13.8 Hz	63.2

Recorded at 500 MHz (^1^H NMR) and 125 MHz (^13^C NMR) in CD_3_OD.

Corndiridoside B (**2**) White amorphous powder; [*α*] −65.6 (0.02, MeOH); UV (MeOH) λ_max_ (log ε) 238 (4.00), 282 (3.24) nm; IR v_max_ 3,392, 2,939, 1,682, 1,639, 1,078 cm^−1^; ^1^H NMR (500 MHz, CD_3_OD) and ^13^C NMR (125 MHz, CD_3_OD) see Table 1; HRESIMS *m/z* 901.29828 [M−H]^−^ (calculated for C_40_H_53_O_23_, 901.29831).

Corndiridoside C (**3**) White amorphous powder; [*α*] −63.1 (0.02, MeOH); UV (MeOH) λ_max_ (log ε) 238 (4.01), 282 (3.13) nm; IR v_max_ 3,424, 2,906, 1,681, 1,639, 1,076 cm^−1^; ^1^H NMR (500 MHz, CD_3_OD) and ^13^C NMR (125 MHz, CD_3_OD) see Table 1; HRESIMS *m/z* 901.29816 [M−H]^−^ (calculated for C_40_H_53_O_23_, 901.29831).

Corndiridoside D (**4**) White amorphous powder; [*α*] −35.6 (0.03, MeOH); UV (MeOH) λ_max_ (log ε) 243 (4.00) nm; IR v_max_ 3,418, 2,922, 1,693, 1,616, 1,077 cm^−1^; ^1^H NMR (500 MHz, CD_3_OD) and ^13^C NMR (125 MHz, CD_3_OD) see Table 2; HRESIMS *m/z* 745.25659 [M−H]^−^ (calculated for C_33_H_45_O_19_, 745.25605).

**TABLE 2:** ^1^H and^13^C NMR data of compounds **4–6**

Position	4		5		6	
	*δ*_H_	*δ*_C_	*δ*_H_	*δ*_C_	*δ*_H_	*δ*_C_
1	5.56, d, *J* = 1.7 Hz	97.6	5.57, d, *J* = 1.4 Hz	98.0	5.50, d, *J* = 1.7 Hz	98.0
3	7.61, d, *J* = 2.4 Hz	153.8	7.61, d, *J* = 2.4 Hz	154.0	7.59, d, *J* = 2.4 Hz	153.9
4	—	106.2	—	105.9	—	106.0
5	3.15, m	28.4	3.13, m	28.5	3.14, m	28.4
6	(a) 1.69, m (b) 1.77, m	25.9	(a) 1.73, m (b) 1.83, m	25.9	(a) 1.69, m (b) 1.77, m	25.9
7	(a) 4.44, m (b) 4.46, m	69.8	(a) 4.38, m (b) 4.46, m	69.6	(a) 4.38, td, *J* = 2.4, 11.7 Hz (b) 4.46, m	69.7
8	5.53, m	133.3	5.56, m	133.5	5.55, m	133.3
9	2.69, m	43.7	2.70, m	44.0	2.70, m	43.8
10	5.29, m	120.8	5.30, dd, *J* = 1.7, 10.4 Hz 5.34, dd, *J* = 1.5, 17.3 Hz	120.9	5.29, m	120.8
11	—	168.7		168.5	—	168.4
12	—	—	—	—	—	—
Glc-1′	4.77, d, *J* = 7.9 Hz	99.1	4.71, d, *J* = 8.0 Hz	99.6	4.67, d, *J* = 7.9 Hz	99.7
2′	3.38, m	73.2	3.23, m	75.4	3.21, m	74.8
3′	3.60, m	85.7	3.55, t, *J* = 8.7 Hz	83.1	3.50, t, *J* = 9.1 Hz	77.4
4′	3.19, m	70.1	3.37, m	70.1	3.59, t, *J* = 9.4 Hz	76.6
5′	3.36, m	78.1	3.40, m	78.0	3.34, m	76.9
6′	(a) 3.56, m (b) 3.83, dd, *J* = 1.7, 11.9 Hz	63.0	(a) 3.67, m (b) 3.90, m	62.8	(a) 3.80, dd, *J* = 4.8, 12.6 Hz (b) 3.88, m	63.3
1″	5.87, d, *J* = 9.4 Hz	96.5	5.90, d, *J* = 9.2 Hz	95.6	5.88, d, *J* = 9.4 Hz	96.0
3″	7.52, s	154.7	7.54, s	154.6	7.52, s	154.5
4″	—	110.8	—	111.7	—	110.7
5″	2.87, m	32.1	3.11, m	27.6	2.82, m	32.0
6″	(a) 1.32, m (b) 2.29, m	35.5	(a) 1.53, m (b) 2.05, m	34.0	(a) 1.23, m (b) 2.20, m	35.6
7″	4.80, d, *J* = 2.3 Hz	103.0	5.34, d, *J* = 3.6 Hz	99.0	4.93, d, *J* = 2.2 Hz	103.5
8″	4.01, m	74.4	4.73, m	66.5	3.92, m	74.1
9″	1.81, m	39.7	1.82, m	40.5	1.79, m	39.8
10″	1.46, d, *J* = 6.8 Hz	19.7	1.32, d, *J* = 6.9 Hz	19.5	1.43, d, *J* = 6.8 Hz	19.7
11″	—	168.5	—	168.8	—	168.6
12″	3.70, s	51.7	3.71, s	51.73	3.70, s	51.7
Glc-1‴	4.74, d, *J* = 7.8 Hz	100.8	4.80, *J* = 7.9 Hz	100.0	4.77, d, *J* = 7.8 Hz	100.3
2‴	3.22, m	74.9	3.23, m	75.0	3.22, m	75.0
3‴	3.27, m	78.9	3.39, m	78.5	3.21, m	78.4
4‴	3.20, m	71.7	3.29, m	71.6	3.30, m	71.4
5‴	3.35, m	77.9	3.30, m	78.3	3.36, m	77.9
6‴	(a) 3.67, m (b) 3.89, dd, *J* = 1.8 12.1 Hz	62.4	(a) 3.70, m (b) 3.92, m	62.9	(a) 3.70, m (b) 3.89, m	62.6

The 1H NMR of compounds 1-6 were recorded at 500 MHz (^1^H NMR) and 125 MHz (^13^C NMR) in CD_3_OD.

Corndiridoside E (**5**) White amorphous powder; [*α*] −27.4 (0.02, MeOH); UV (MeOH) λ_max_ (log ε) 244 (4.20) nm; IR v_max_ 3,393, 2,912, 1,696, 1,617, 1,074 cm^−1^; ^1^H NMR (500 MHz, CD_3_OD) and ^13^C NMR (125 MHz, CD_3_OD) see Table 2; HRESIMS *m/z* 745.25513 [M−H]^−^ (calculated for C_33_H_45_O_19_, 745.25605).

Corndiridoside F (**6**) White amorphous powder [*α*] −31.6 (0.02, MeOH); UV (MeOH) λ_max_ (log ε) 239 (4.20) nm; IR v_max_ 3,403, 2,927, 1,697, 1,618, 1,074 cm^−1^; ^1^H NMR (500 MHz, CD_3_OD) and ^13^C NMR (125 MHz, CD_3_OD) see Table 2; HRESIMS *m/z* 745.25549 [M−H]^−^ (calculated for C_33_H_45_O_19_, 745.25605).

### 2.4 Acid hydrolysis and determination of the stereochemistry of the sugar moiety of **1–6**

The acid hydrolysis method of each compound has previously been reported (Wang et al., 2018). Each compound (**1–6,** 1.0 mg) was dissolved in 2 M HCl (2.0 mL) and reacted at 80°C for 1.5 h. EtOAc was used to extract the reaction mixture and obtain an H_2_O layer. After evaporation and dilution by H_2_O, a neutral residue was obtained. Then, anhydrous pyridine (1.0 mL) and 1 mg of L-cysteine methyl ester hydrochloride was added. After stirring at 60°C for 2 h, the mixture was evaporated to dryness, and 0.1 mL of N-trimethylsilyl imidazole was added. After stirring 2 h at 60°C, the reaction mixture was extracted with n-hexane. The n-hexane extract was subjected to GC analysis with an HP-5 capillary column (30 m, 0.25 mm, Dikma), FID detection, 280°C detector temperature, and a stepwise heating from 160°C to 280°C at 5°C/min, using N_2_ as the carrier gas. Compared with the retention time of D-glucose (20.55 min), the sugar of all compounds was found to be D-glucose.

### 2.5 Anti-inflammatory activity test in LPS-stimulated RAW264.7 cells

The anti-inflammatory activity of the isolated compounds was determined by a previously reported method (Xu et al., 2024). The cell viability assay was measured using CCK-8 assay and NO production in LPS-stimulated RAW264.7 cells was measured using Griess assay. All experiments were performed in triplicate. Data were processed using GraphPad Prism 9.0.

## 3 Results and discussion

### 3.1 Structure elucidation

**FIGURE 1:**
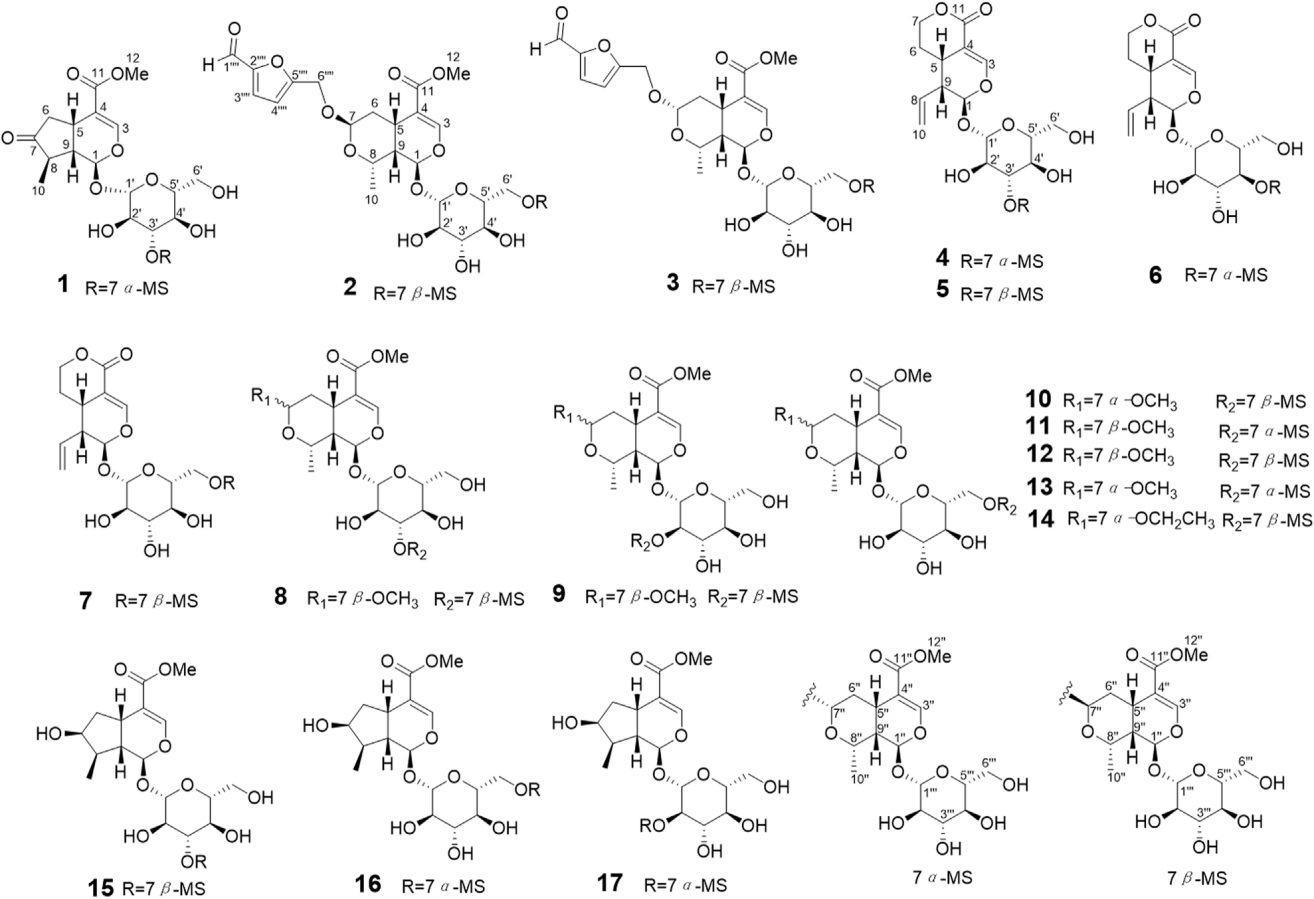
The structures of compounds **1–17**.

Guided by the anti-inflammatory activity test, six new iridoid glycoside dimers (**1–6**) and eleven known iridoid glycoside dimers (**11–17**) were isolated from the fraction with strong anti-inflammatory activity using chromatography techniques (Figure 1).

Compound **1** was obtained as an amorphous white powder. HRESIMS data exhibited an ion at *m/z* 775.26660 [M−H]^−^, confirming the molecular formula to be C_34_H_48_O_20_ with an unsaturation of 11. The IR spectra had absorption bands at 3,401 cm^−1^ (hydroxyl group), 1737 and 1703 cm^−1^ (carbonyl groups). The ^1^H NMR data (Table 1) of compound **1** displayed two olefinic protons at *δ*_H_ 7.52 (1H, s, H-3″) and 7.51 (1H, d, *J* = 1.1 Hz, H-3), four oxygenated methine protons at *δ*_H_ 5.66 (1H, d, *J* = 2.8 Hz, H-1), 5.88 (1H, d, *J* = 9.3 Hz, H-1″), 4.79 (1H, dd, *J* = 9.6, 2.3 Hz, H-6″), and 4.01 (1H, m, H-8″), two methoxy protons at *δ*_H_ 3.71 (3H, s, H-12) and 3.70 (3H, s, H-12″), and a series of glycosyl protons. The molecular formula and ^13^C NMR data suggested that structure **1** could be an iridoid dimer. ^13^C NMR data combined with HSQC spectrum suggested the presence of one ketone carbonyl carbon signal (*δ*_C_ 220.8), two ester carbonyl carbon signals (168.6 and 168.9), four olefin carbon signals (*δ*_C_ 111.1, 153.2, 110.8, 154.7), two methoxy carbon signals (*δ*_C_ 51.8 and 51.7), two secondary methyl carbon signals (*δ*_C_ 13.5 and 19.7), five acetal carbon signals (*δ*_C_ 94.9, 96.4, 99.7, 100.7, 103.3) and one oxygenated methine carbon signal (*δ*_C_ 74.4). A series of signals for two glycosyl compounds were displayed at *δ*_C_ 86.0, 78.7, 78.2, 78.0, 74.9, 73.2, 71.8, 70.2, 62.9, and 62.5. A comparison of the data for **1** with those of 7*α-*morroniside (Han et al., 2004) and 7-dehydrologanin (Chen et al., 2017) suggested that **1** might be a condensation product of the above two compounds.

**FIGURE 2:**
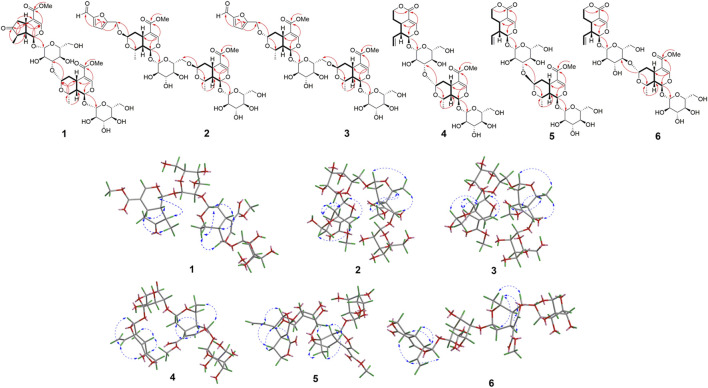
The ^1^H-^1^H COSY (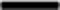) HMBC (
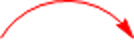
) and NOESY (
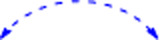
) correlations of compounds **1–6**.

Comprehensive analysis of the two dimensional NMR (2D NMR) data displayed the planar structure of **1** (Figure 1). ^1^H-^1^H COSY spectrum demonstrated the spin system involving C6-C5-C9-C1 and C8-C9, combined with the HMBC correlations from H-5 to C-7, from H-10 to C-9 and C-7, from H-1 to C-3, C-5 and C-1″, from H-6 to C-4, from H-3 to C-11, and from H-12 to C-11 indicated the presence of a 7-dehydrologanin unit (Figure 2). Additionally, ^1^H-^1^H COSY correlations of H-1′′/H-9″, H-9′′/H-8″, H-9′′/H-5″, H-5′′/H-6″ combined with HMBC correlations from H-8″ to C-5″ and C-7″, from H-7″ to C-5″, H-3″ to C-5″ and C-1‴ established the 7*α-*morroniside unit. The key HMBC correlations of H-7″ with C-3′ suggested the linkage of C-7″-O-C-3′. The ROESY spectra exhibited correlations of H-1/H-8, H-1′′/H-10″, suggesting H-1, H-8, H-1″, and H-10″ were co-facial and defined as *α*-orientation (Figure 2). Consequently, ROESY correlations of H-10/H-5, H-5/H-9, H-8′′/H-7′′/H-5″, H-5′′/H-9″ indicated the H-5, H-9, H-10, H-5″, H-7″, H-8″ and H-9″ were in *β*-oriented. Based on the fact that iridoid compounds in *C. officinalis* are all 5*β* and 9*β* configurations, according to the coupling constants and chemical shifts of H-1, H-1″, H-7″, the absolute configurations of C-1, C-1″, and C-7 were determined to be 1*R*, 1″*R* and 7″*S*. Furthermore, the configurations of C-1, C-5, C-8, C-9, C-1″, C-5″, C-7″, C-8″ and C-9″ were identified as 1*R*, 5*S*, 8*R*, 9*S*, 1″*R*, 5″*S*, 7″*S*, 8″*S* and 9″*S*, which were consistent with 7*α-*morroniside (Han et al., 2004) and 7-dehydrologanin (Chen et al., 2017). After acid hydroxylation and derivatization, **1** was confirmed as D-glucose by GC analysis. The coupling constants of the anomeric proton signal at *δ*_H_ 4.75 (1H, d, *J* = 7.8 Hz, H-1′) and 4.76 (1H, d, *J* = 7.8 Hz, H-1‴) confirmed the *β*-configuration of D-glucose. Compound **1** was assigned as corndiridoside A.

Compound **2** was obtained as an amorphous powder with the molecular formula C_40_H_54_O_23_ by HRESIMS *m/z* 901.29828 [M-H]^-^ analysis. Its ^1^H NMR displayed four olefin protons at *δ*_H_ 6.67 (1H, d, *J* = 3.5 Hz, H-3′′′′), 7.38 (1H, d, *J* = 3.5 Hz, H-4′′′′), 7.51 (1H, s, H-3″), and 7.52 (1H, s, H-3), six acetal protons at *δ*_H_ 4.80 (1H, d, *J* = 7.9 Hz, H-1‴), 4.81 (1H, *J* = 7.9 Hz, H-1′), 4.87 (1H, d, *J* = 3.2 Hz, H-7″), 4.92 (1H, d, *J* = 3.2 Hz, H-7), 5.84 (1H, d, *J* = 9.3 Hz, H-1″), and 5.90 (1H, d, *J* = 9.3 Hz, H-1). There are two oxygenated methine protons at *δ*_H_ 4.30 (1H, m, H-8″) and 4.41 (1H, m, H-8), and oxygenated methylene protons at *δ*_H_ 4.59 (1H, d, *J* = 13.7 Hz, H-6a′′′′) and 4.63 (1H, d, *J* = 13.7 Hz, H-6b′′′′); a series of protons displayed between *δ*_H_ 3.24 ∼ 3.97 correspond to the sugar moieties. The ^13^C NMR data showed 40 carbon signals, including two carbonyl carbon signals at *δ*_C_ 168.6 and 168.6, one ketone carbonyl carbon signals at *δ*_C_ 179.5, eight olefin carbon signals at *δ*_C_ 111.7, 111.7, 112.9, 124.9, 154.2, 154.5, 154.6, and 160.0, six acetal carbon signals at *δ*_C_ 95.7, 95.8, 97.9, 99.1, 100.0, and 100.4, two oxygenated methine carbon signals at *δ*_C_ 62.8 and 66.3, one oxygenated methylene carbon signal at *δ*_C_ 61.9, two methoxy carbon signals at *δ*_C_ 51.7. These NMR data showed close similarity to those of 7*β-*morroniside (Han et al., 2004). Combined with the molecular formula, **2** was speculated as a 7*β-*morroniside dimer with a 5-hydroxymethylfurfuraly moiety. The planar structure of **2** (Figure 1) was confirmed by the comprehensive analysis of the 2D NMR data (Figure 2). The key HMBC correlations of H-7″ with C-6′ indicated linkage of C-6′-O-C-7″ between two 7*β-*morroniside moieties. The structure of 5-hydroxymethylfurfuraly moiety was determined by the key HMBC correlations of H-3′′′′ with C-1′′′′ and C-5′′′′, H-4′′′′ with C-2′′′′ and C-6′′′′, which was connected to 7*β-*morroniside through 7-O-C-6′′′′ according to the HMBC correlations of H-7 with C-6′′′′. The configuration was found similar to that of the 7*β-*morroniside moieties by the NOESY correlations analysis as shown in Figure 2 and consistent with 7*β-*morroniside. Thus, compound **2** was determined as corndiridoside B.

Compound **3** had the same molecular formula as **2** according to HREISMS (*m/z* 901.29816 [M-H]^-^) and NMR data (Table 1). The NMR spectrum of compound **3** was highly similar to that of **2**, except for the chemical downshift of C-7″ and C-8′′ (from *δ*_C_ 99.1, 66.5 to *δ*_C_ 102.4, 74.1), which indicated that one of the 7*β-*morroniside in **2** was replaced by 7*α-*morroniside in **3**. HMBC correlation of H-7″ with C-6′ confirmed that 7*α-*morroniside and 7*β-*morroniside were connected through C-6′-O-C-7′′ bond (Figure 2). The HMBC correlation of H-7 with C-6′′′′ indicated that the 5-hydroxymethylfurfural moiety was linked to 7*α*-morroniside via an ester bond. Furthermore, the NOESY correlation between H-7″ and H-8*β* confirmed that H-7 was the *β*-oriented, and the absolute configuration was the same as 7*α-*morroniside and 7*β-*morroniside; hydrolysis and GC analysis proved that the sugar groups were D-glucose, so compound **3** was identified as corndiridoside C.

Compound **4** was a white amorphous powder. The molecular formula of C_33_H_46_O_19_ was determined by the HRESIMS ion at *m/z*: 745.25659 [M−H]^−^. The ^1^H NMR data of **4** (Table 2) showed two four olefin protons, including two terminal olefinic protons at *δ*_H_ 5.53 (1H, m, H-8) and 5.29 (2H, m, H-10) and two olefin protons at *δ*_H_ 7.52 (1H, s, H-3″), 7.61 (1H, d, *J* = 2.4 Hz, H-3), five acetal protons at *δ*_H_ 5.87 (1H, d, *J* = 9.4 Hz, H-1″), 4.74 (1H, d, *J* = 7.8 Hz, H-1‴), 4.77 (1H, d, *J* = 7.9 Hz, H-1′), 5.56 (1H, d, *J* = 1.7 Hz, H-1), 4.80 (1H, d, *J* = 2.3 Hz, H-7″), one oxygenated methylene proton at *δ*_H_ 4.44 (1H, m, H-7a) and 4.46 (1H, m, H-7b), one oxygenated methine proton at *δ*_H_ 4.01 (1H, m, H-8″), one methoxy protons at *δ*_H_ 3.70 (3H, s, H-12″), and a series of protons at *δ*_H_ 3.19 ∼ 4.78 were assigned to the glycosyl groups. ^13^C NMR and HSQC data were assigned to 33 carbon signals. Among them, there was one methyl carbon (*δ*_C_ 19.7), one methoxy carbon signal (*δ*_C_ 51.7), two oxygenated olefin carbon signals (*δ*_C_ 154.7, 153.8), four olefin carbon signals (*δ*_C_ 106.2, 110.8, 133.3, 120.8), five acetal carbon signals (*δ*_C_ 103.0, 96.5, 100.8, 97.6, 99.1), one oxygenated methylene carbon signal (*δ*_C_ 69.8), one oxygenated methine carbon signal (*δ*_C_ 74.4), two methylene carbon signals (*δ*_C_ 35.5 and 25.9), four methine carbon signals (*δ*_C_ 28.4, 43.7, 32.1, 39.7), two carbonyl carbon signals (*δ*_C_ 168.7 and 168.5), and two sets of glycosyl carbons (*δ*_C_ 62.4 ∼ 100.8). The above data of **4** were very similar to those of 7*α-*morroniside (Han et al., 2004) and sweroside (Chen et al., 2017), indicating that **4** was an iridoid glycoside dimer. Detailed 2D NMR analysis confirmed the structures of the two moieties (Figure 2). In the ^13^C NMR data of sweroside, the chemical shift of C-2′ was significantly shifted up (from *δ*_C_ 74.9 to 73.2) and the chemical shift of C-3′ was significantly shifted down (from *δ*_C_ 78.9 to 85.7), indicating that sweroside and 7*α-*morroniside were connected via C-3′-O-C-7″, which was also confirmed by the HMBC correlation from H-7″ to C-3′. The NOESY spectral correlations of H-8/H-1, H-8/H-6a, H-6b/H-5, H-6b/H-9, H-1′′/H-10″, H-8′′/H-5″, H-8′′/H-7″, H-5′′/H-7″ and H-5′′/H-9″, combined with chemical shift and coupling constants, thereby confirming the structure of compound **4**, which was named corndiridoside D.

Compound **5** has the molecular formula C_33_H_46_O_19_ by the HRESIMS ion at *m/z*: 745.25513 [M-H]^-^. Its NMR data (Table 2) was consistent with compound **4**, except for the obvious chemical upshift of C-7′′ (**4**, *δ*_C_ 103.0; **5**, *δ*_C_ 99.0 and C-8′′ (**4**, *δ*_C_ 74.4; **5**, *δ*_C_ 66.5) indicated the presence of 7*β*-morroniside unit in **5** instead of 7*α*-morroniside in **4**. The linkage of C-3′-O-C-7″ between two units was confirmed by the HMBC correlations of H-7″ and C-3′ (Figure 2). Compound **5** was determined as corndiridoside E.

Compound **6** has the same molecular formula of C_33_H_46_O_19_ as compounds **4** and **5** based on the HREISMS (*m/z*: 745.25549 [M-H]^-^) and NMR data (Table 2). The NMR data of **6** was very similar to those of **4,** except for the chemical shifts of C-3′, C-4′, and C-5′. The upshift of C-3′ (from *δ*_C_ 85.7 in **4** to *δ*_C_ 77.4 in **6**), C-5′ (from *δ*_C_ 78.1 in **4** to *δ*_C_ 76.9 in **6**) and downshift of C-4′ (from *δ*_C_ 70.1 in **4** to *δ*_C_ 76.6 in **6**) indicated that the 7*α-*morroniside moiety was linked via C-4′. HMBC correlation analysis of H-7″ at *δ*_H_ 4.93 and C-4′ at *δ*_C_ 76.6 confirmed that the C-7″ and C-4″ were lined via an ether bond (Figure 2). Thus, compound **6** was determined as corndiridoside F.

Comparing the NMR and HRESIMS data with literatures, 11 known compounds were identified as: cornuofficinaliside L (**7**) (Hao et al., 2023), cornuside C (**8**) (Ye et al., 2017), cornuside B (**9**) (Ye et al., 2017), cornuside E (**10**) (Ye et al., 2017), cornuside J (**11**) (Ye et al., 2017), cornuside A (**12**) (Ye et al., 2017), cornuside G (**13**) (Ye et al., 2017), cornuside K (**14**) (Ye et al., 2017), cornuofficinali side D (**15**) (Hao et al., 2023), cornuside L (**16**) (Ye et al., 2017), cornuside M (**17**) (Ye et al., 2017).

### 3.2 Anti-inflammatory effects of compounds **1–17**

**FIGURE 3:**
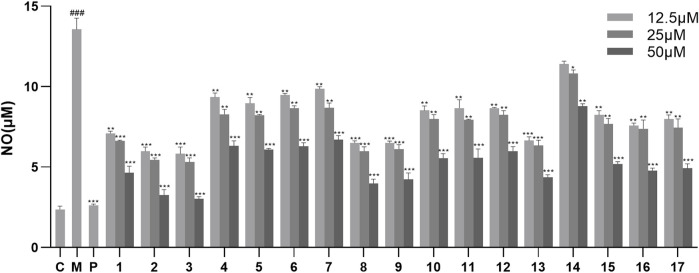
Effects of NO production in LPS-stimulated RAW246.7 cells treated with compounds **1–17**. Data are expressed as mean ± S. D (n = 3). C = control, M = model, P = hydrocortisone. *p < 0.05, **p < 0.01 and ***p < 0.001 vs. LPS-simulated group, ###p < 0.001 vs*.* control group.

The inhibitory effects of the isolated compounds on NO production in LPS-stimulated RAW264.7 cells were evaluated. First, the cell viability assays exhibited that compounds **1–17** had no cytotoxic effect on RAW264.7 cells at a concentration below 50 μM (*p* > 0.05) (Supplementary Figure S1). Therefore, the inhibitory activity of compounds **1–17** on NO production in LPS-stimulated RAW264.7 cells was measured at concentrations of 12.5, 25, and 50 μM. As the results showed (Figure 3; Supplementary Table S1), compounds **1–17** showed significant anti-inflammatory activity at concentrations of 25 and 50 μM. Among them, compounds **2** and **3** exhibited the strongest anti-inflammatory activity in a dose-dependent manner, thereby suggesting that the 5-hydroxymethylfurfural group might enhance the activity. In addition, compound **14** showed the weakest anti-inflammatory activity compared with the other compounds, suggesting that the ethoxy group at the C-7 position might reduce its anti-inflammatory activity.

## 4 Conclusion

In summary, six new iridoid glycoside dimers, named corndiridoside A-F (**1–6**), and eleven known analogs (**7–17**) were isolated from the anti-inflammatory active fraction of *C. officinalis* fruits in the current study. The constituent units in these dimers are composed of morrnoiside, 7-dehydrologanin, and sweroside analogs. Among them, compound **1,** a dimer containing 7-dehydrologanin unit, was discovered for the first time from *C. officinalis*. In addition, their anti-inflammatory activities were assayed on the LPS-stimulated 264.7 RAW cell model. All compounds showed no cytotoxic effect on the cell viability of 264.7 RAW cells at 50 μM, and a majority of compounds exhibited significant anti-inflammatory activity at concentrations of 12.5, 25, and 50 μM. Compounds **2** and **3** containing 5-hydroxymethylfurfural group showed the strongest anti-inflammatory, indicating that the 5-hydroxymethylfurfural group might play an important role in enhancing anti-inflammatory activity. These compounds with anti-inflammatory activity may represent promising natural anti-inflammatory compounds that can be used in the development of drugs and functional foods. Moreover, this study also provides a basic scientific basis for the clinical anti-inflammatory application of *C. officinalis*.

## Data Availability

The original contributions presented in the study are included in the article/Supplementary Material, further inquiries can be directed to the corresponding author.
