# Supplementary material for: Iridoid glycoside dimers from fruits of Cornus officinalis and their anti-inflammatory activity
Source: Front Chem. 2025 Mar 17;13:1558075. doi: 10.3389/fchem.2025.1558075 (PMC11955624; doi:10.3389/fchem.2025.1558075)
Supplement: Supplementary file 1 [file DataSheet1.pdf]

Supplementary Material

Iridoid Glycoside Dimers from Fruits of *Cornus officinlis* and Their Anti-inflammatory Activity

Ying-Chu Shi[†1, 2], Yu-Xin Yu[†1, 2], Jiu-Xia Gao[1, 2], Xin Wang[3], Xiao-Ya Shang[3], Jia Xu[1*]

[1] Beijing University of Chinese Medicine, Beijing, 100105, China

[2] Beijing Hospital of Traditional Chinese Medicine, Capital Medical University, Beijing,100010, China

[3] College of Applied Arts and Science, Beijing Union University, Beijing 100191, China

***** Correspondence: doctorxujia@163.com;

[†] These authors contributed equally

List of supplementary materials	
1	**Figure S1** The cytotoxic of compounds **1**-**17** on RAW264.7 cell
2	**Table S1** Effect of compounds 1-17 on NO production in RAW246.7 cells
3	**Figure S2** HRESIMS spectrum of compound **1**
4	**Figure S3** IR spectrum of compound **1**
5	**Figure S4.** ^{1}H NMR spectrum of compound **1**
6	**Figure S5** ^{13}C NMR spectrum of compound **1**
7	**Figure S6** HSQC spectrum of compound **1**
8	**Figure S7** ^{1}H-^{1}H COSY spectrum of compound **1**
9	**Figure S8** HMBC spectrum of compound **1**
10	**Figure S9** ROESY spectrum of compound **1**
11	**Figure S10** HRESIMS spectrum of compound **2**
12	**Figure S11** IR spectrum of compound **2**
13	**Figure S12** ^{1}H NMR spectrum of compound **2**
14	**Figure S13** ^{13}C NMR spectrum of compound **2**
15	**Figure S14** HSQC spectrum of compound **2**
16	**Figure S15** ^{1}H-^{1}H COSY spectrum of compound **2**
17	**Figure S16** HMBC spectrum of compound **2**
18	**Figure S17** ROESY spectrum of compound **2**
19	**Figure S18** HRESIMS spectrum of compound **3**
20	**Figure S19** IR spectrum of compound **3**
21	**Figure S20** ^{1}H NMR spectrum of compound **3**
22	**Figure S21** ^{13}C NMR spectrum of compound **3**
23	**Figure S22** HSQC spectrum of compound **3**
24	**Figure S23** ^{1}H-^{1}H COSY spectrum of compound **3**
25	**Figure S24** HMBC spectrum of compound **3**
26	**Figure S25** ROESY spectrum of compound **3**
27	**Figure S26** HRESIMS spectrum of compound **4**
28	**Figure S27** IR spectrum of compound **4**
29	**Figure S28** ^{1}H NMR spectrum of compound **4**
30	**Figure S29** ^{13}C NMR spectrum of compound **4**
31	**Figure S30** HSQC spectrum of compound **4**
32	**Figure S31** ^{1}H-^{1}H COSY spectrum of compound **4**
33	**Figure S32** HMBC spectrum of compound **4**
34	**Figure S33** ROESY spectrum of compound **4**
35	**Figure S34** HRESIMS spectrum of compound **5**
36	**Figure S35** IR spectrum of compound **5**
37	**Figure S36** ^{1}H NMR spectrum of compound **5**
38	**Figure S37** ^{13}C NMR spectrum of compound **5**
39	**Figure S38** HSQC spectrum of compound **5**

**40**	**Figure S39** ^{1}H-^{1}H COSY spectrum of compound **5**
**41**	**Figure S40** HMBC spectrum of compound **5**
**42**	**Figure S41** ROESY spectrum of compound **5**
**43**	**Figure S42** HRESIMS spectrum of compound **6**
**44**	**Figure S43** IR spectrum of compound **6**
**45**	**Figure S44** ^{1}H NMR spectrum of compound **6**
**46**	**Figure S45** ^{13}C NMR spectrum of compound **6**
**47**	**Figure S46** HSQC spectrum of compound **6**
**48**	**Figure S47** ^{1}H-^{1}H COSY spectrum of compound **6**
**49**	**Figure S48** HMBC spectrum of compound **6**
**50**	**Figure S49** ROESY spectrum of compound **6**
**51**	**Figure S50** ^{1}H NMR spectrum of compound **7**
**52**	**Figure S51** ^{13}C NMR spectrum of compound **7**
**53**	**Figure S52** ^{1}H NMR spectrum of compound **8**
**54**	**Figure S53** ^{13}C NMR spectrum of compound **8**
**55**	**Figure S54** ^{1}H NMR spectrum of compound **9**
**56**	**Figure S55** ^{13}C NMR spectrum of compound **9**
**57**	**Figure S56** ^{1}H NMR spectrum of compound **10**
**58**	**Figure S57** ^{13}C NMR spectrum of compound **11**
**59**	**Figure S58** ^{1}H NMR spectrum of compound **12**
**60**	**Figure S59** ^{13}C NMR spectrum of compound **12**
**61**	**Figure S60** ^{1}H NMR spectrum of compound **13**
**62**	**Figure S61** ^{13}C NMR spectrum of compound **13**
**63**	**Figure S62** ^{1}H NMR spectrum of compound **14**
**64**	**Figure S63** ^{13}C NMR spectrum of compound **14**
**65**	**Figure S64** ^{1}H NMR spectrum of compound **15**
**66**	**Figure S65** ^{13}C NMR spectrum of compound **15**
**67**	**Figure S66** ^{1}H NMR spectrum of compound **16**
**68**	**Figure S67** ^{13}C NMR spectrum of compound **16**
**69**	**Figure S68** ^{1}H NMR spectrum of compound **17**
**70**	**Figure S69** ^{13}C NMR spectrum of compound **17**

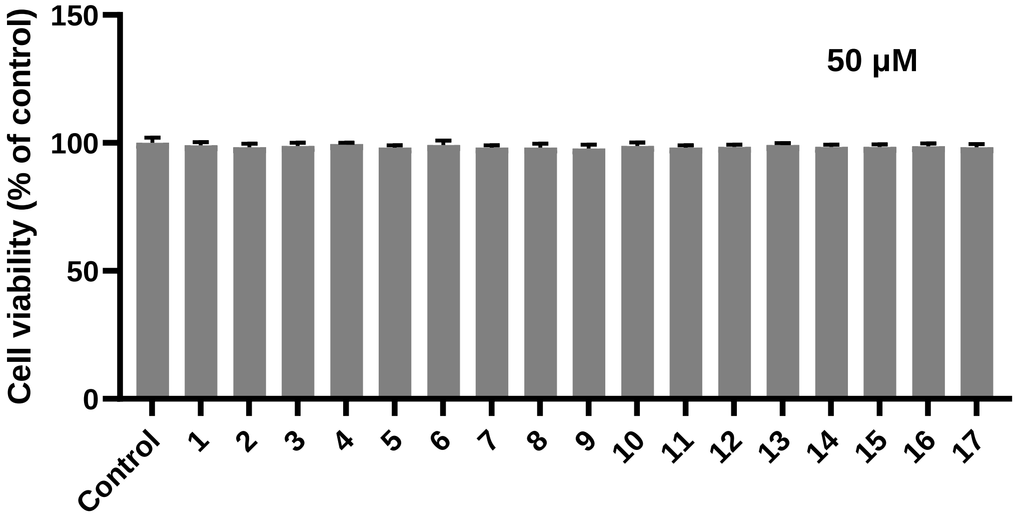

Figure S1 The cytotoxic of compounds **1**-17 on RAW264.7 cell

Table S1 Effect of compounds **1-17** on NO production in RAW246.7 cells.

**Compound**	**NO production (µM)**			
		**50 µM**	**25 µM**	**12.5 µM**
**Control**	2.42 ± 0.11			
**LPS**	13.30 ± 0.82$^{\#\#\#}$			
**1**		4.64 ± 0.42***	6.62 ± 0.05***	7.10 ± 0.14**
**2**		3.26 ± 0.34***	5.44 ± 0.13***	5.99 ± 0.26***
**3**		3.02 ± 0.13***	5.30 ± 0.26***	5.82 ± 0.42***
**4**		6.30 ± 0.34***	8.28 ± 0.30**	9.34 ± 0.27**
**5**		6.08 ± 0.08***	8.24 ± 0.03**	8.97 ± 0.34**
**6**		6.29 ± 0.21***	8.67 ± 0.13**	9.47 ± 0.11**
**7**		6.69 ± 0.27***	8.68 ± 0.30**	9.86 ± 0.14**
**8**		3.98 ± 0.26***	5.98 ± 0.28***	6.50 ± 0.13***
**9**		4.23 ± 0.40***	6.12 ± 0.28***	6.51 ± 0.11***
**10**		5.56 ± 0.27***	7.99 ± 0.26**	8.52 ± 0.27**
**11**		5.58 ± 0.53***	7.93 ± 0.03**	8.67 ± 0.51**
**12**		6.00 ± 0.27***	8.25 ± 0.26**	8.67 ± 0.04**
**13**		4.37 ± 0.15***	6.33 ± 0.34***	6.65 ± 0.23***
**14**		8.79 ± 0.15**	10.82 ± 0.21*	11.41 ± 0.18
**15**		5.20 ± 0.13***	7.68 ± 0.34**	8.24 ± 0.26**
**16**		4.78 ± 0.15***	7.38 ± 0.55**	7.59 ± 0.15**
**17**		4.92 ± 0.28***	6.62 ± 0.05***	7.10 ± 0.14**
**Hydrocortisone**		-	2.62 ± 0.15***	-

Data are expressed as mean ± S.D. (n = 3). The statistical analysis was evaluated using one-way analysis of variance followed by Tukey's multiple comparison test. *$p < 0.05$, **$p < 0.01$ and ***$p < 0.001$ vs LPS-treated group, $^{\#\#\#}p <$ 0.001 vs control group.

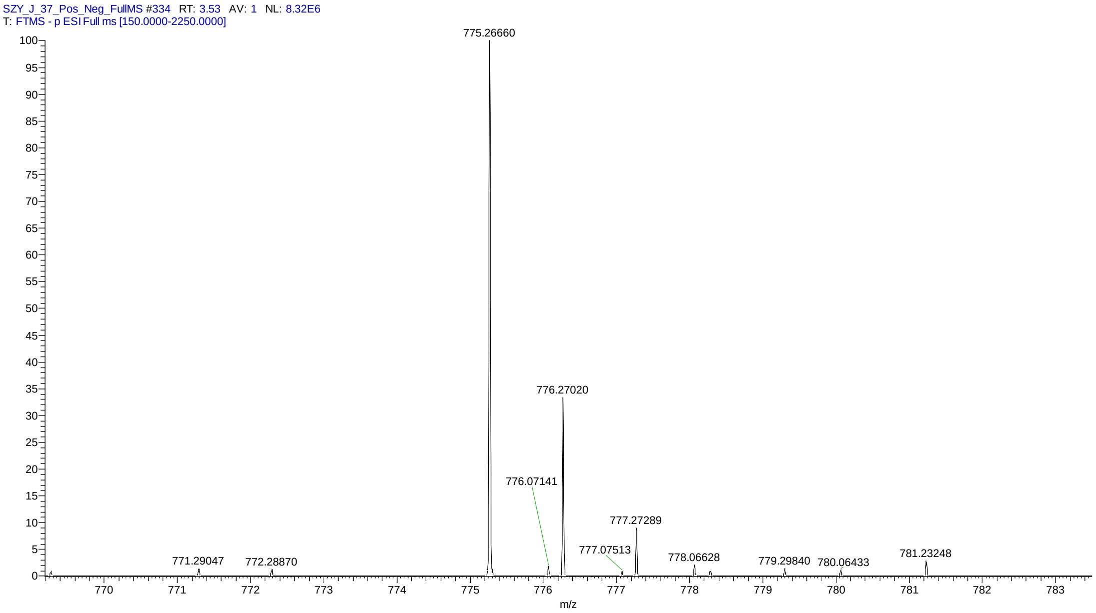

Figure S2 HRESIMS spectrum of compound **1**

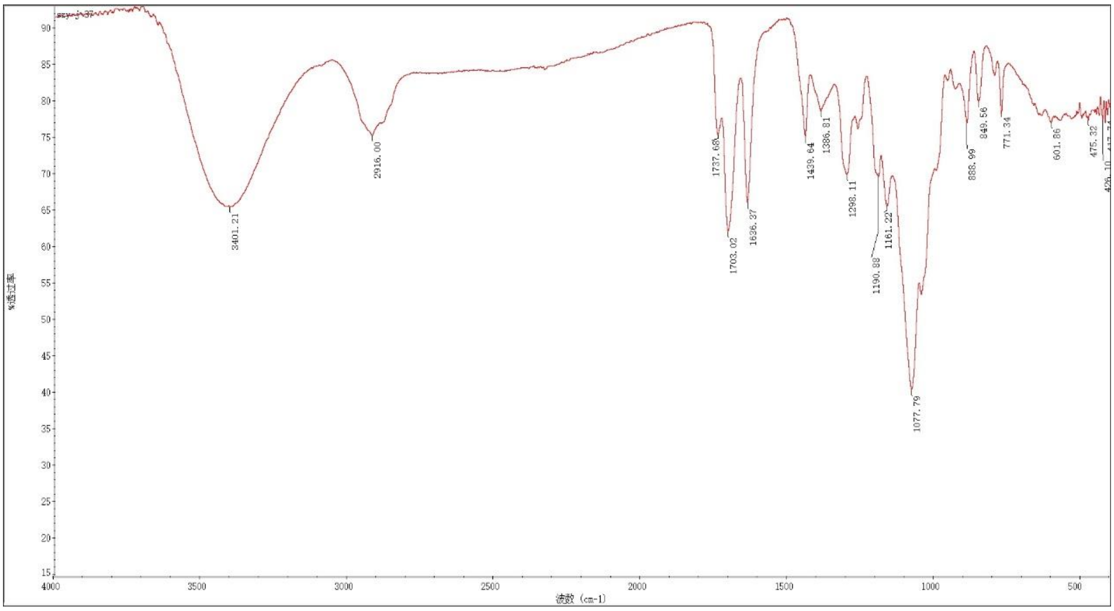

Figure S3 IR spectrum of compound **1**

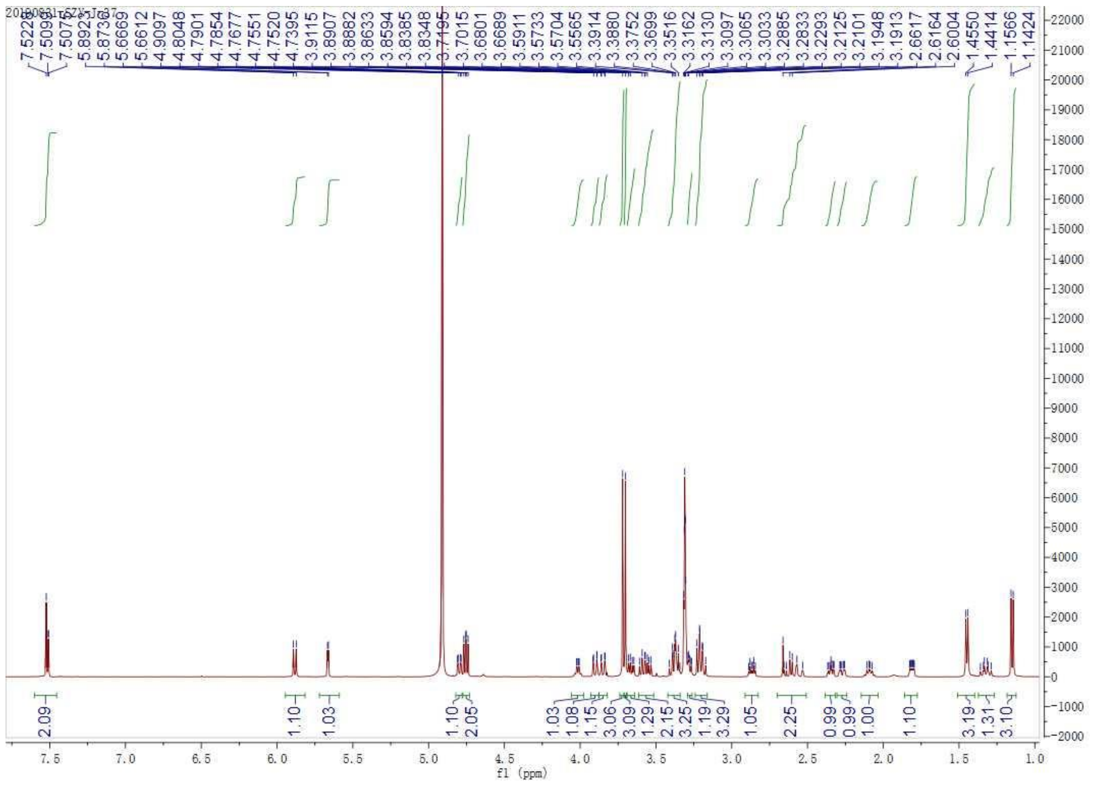

Figure S4. ^{1}H NMR spectrum of compound **1**

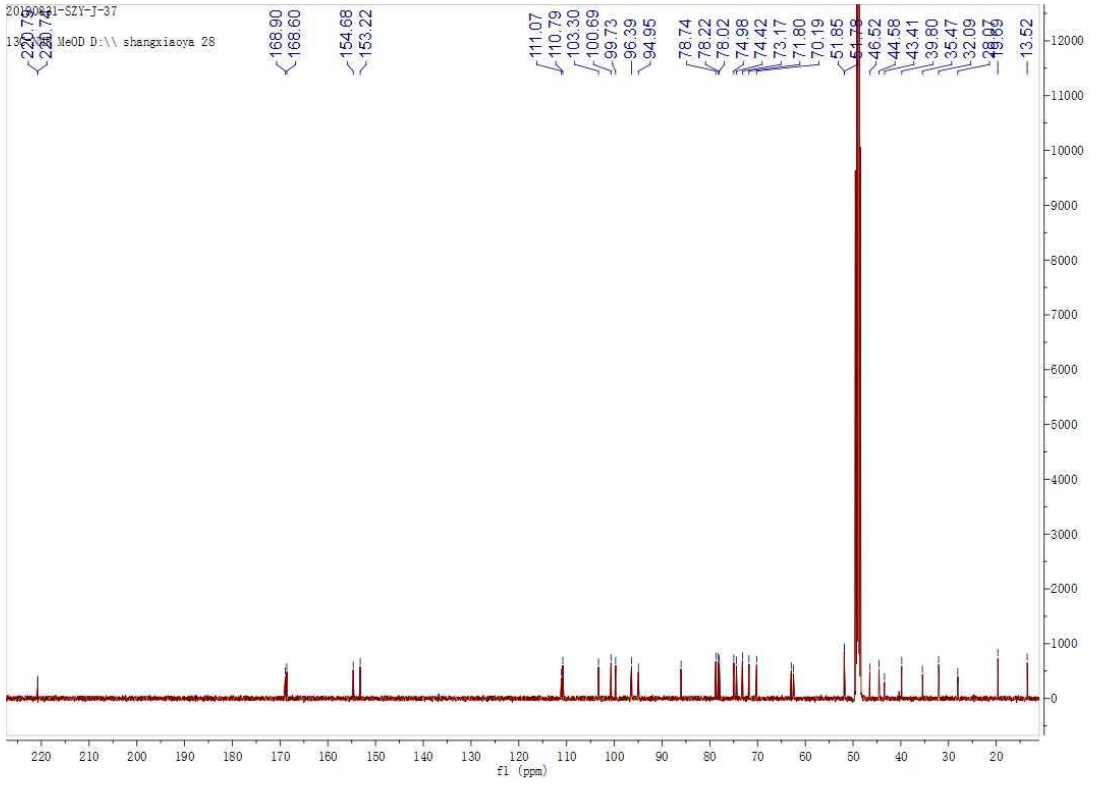

Figure S5 ^{13}C NMR spectrum of compound **1**

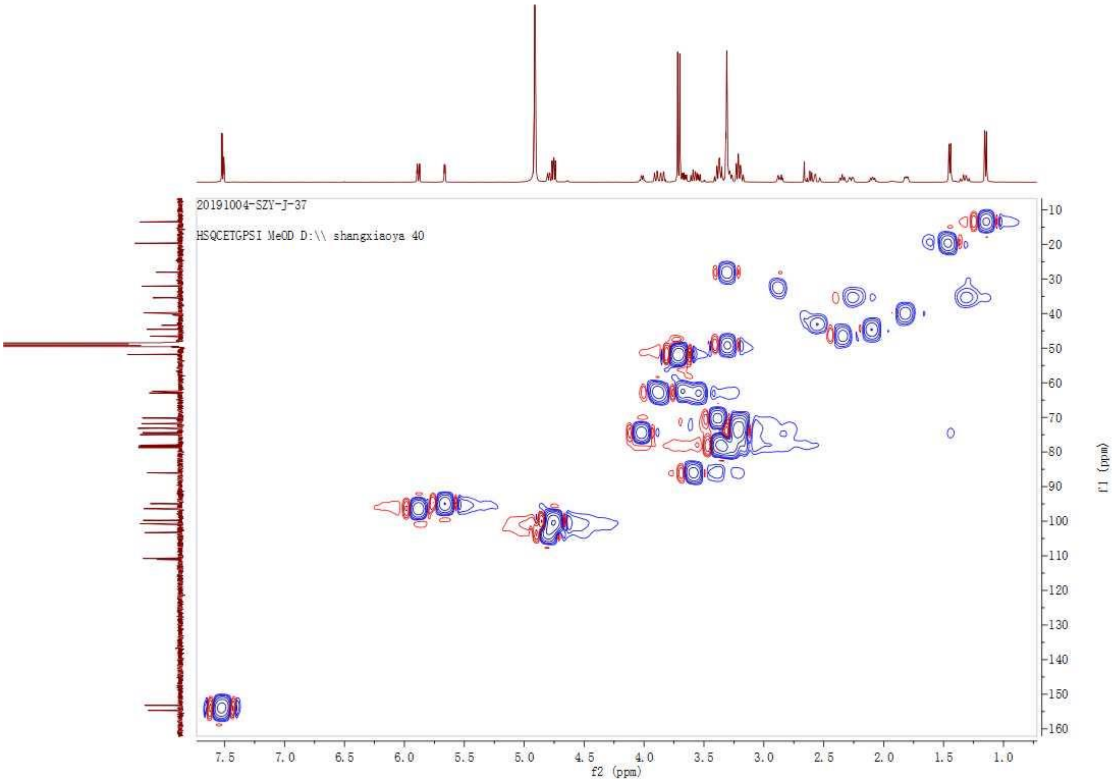

Figure S6 HSQC spectrum of compound **1**

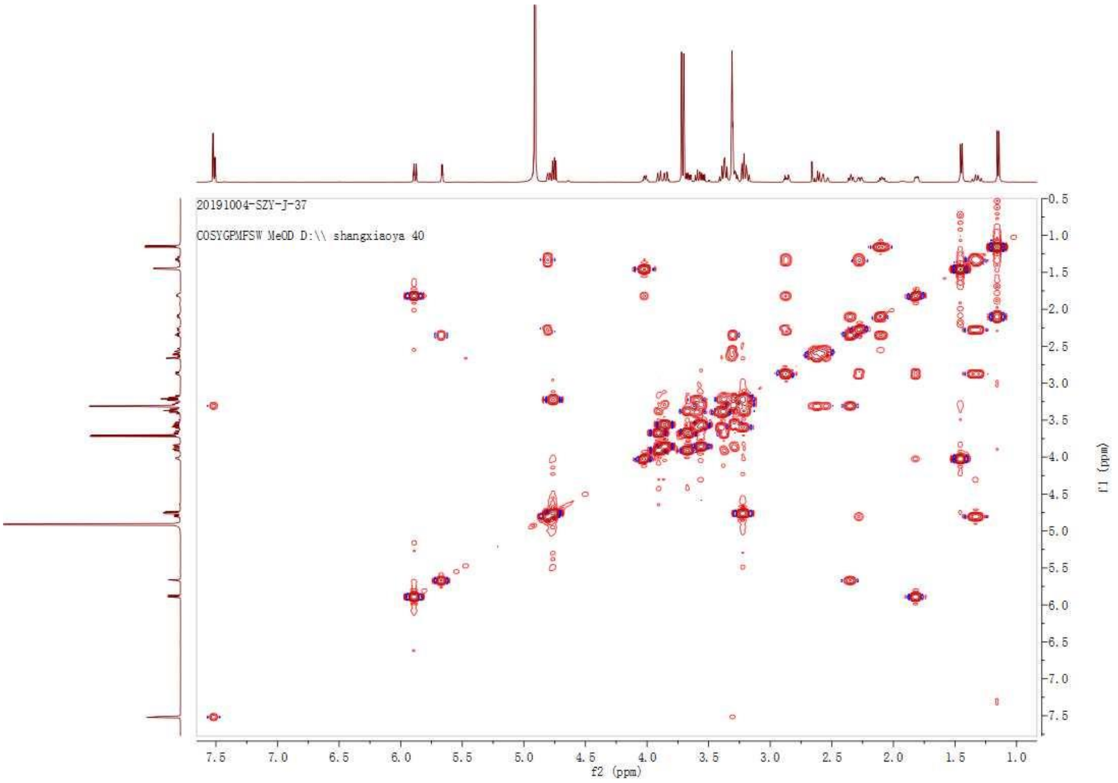

Figure S7 ^{1}H-^{1}H COSY spectrum of compound **1**

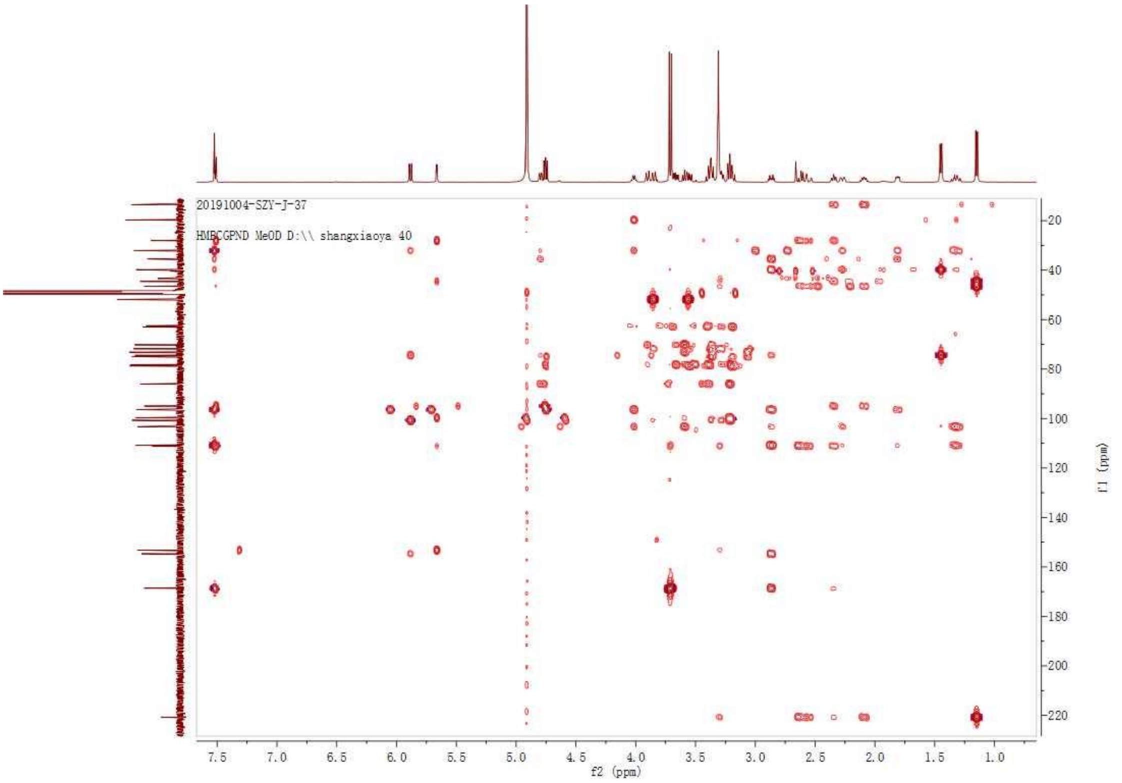

Figure S8 HMBC spectrum of compound **1**

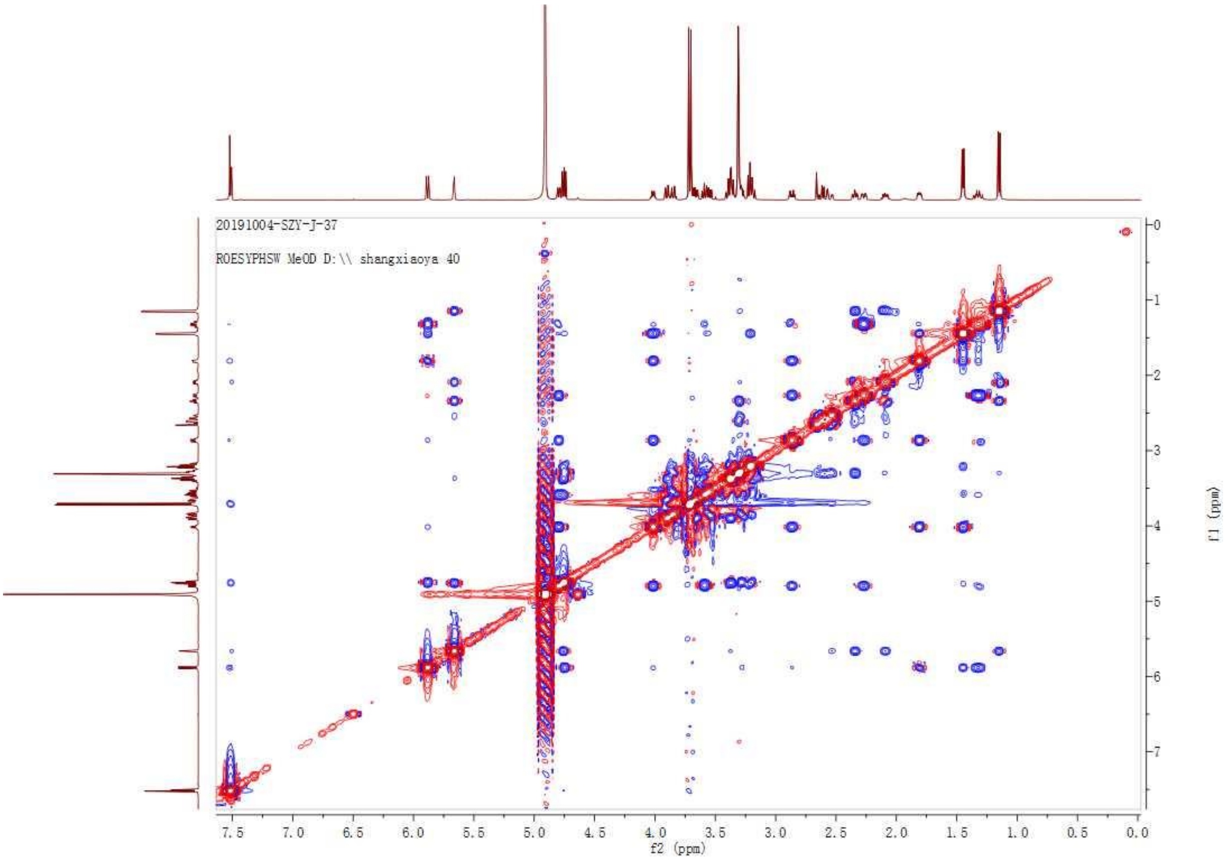

Figure S9 ROESY spectrum of compound **1**

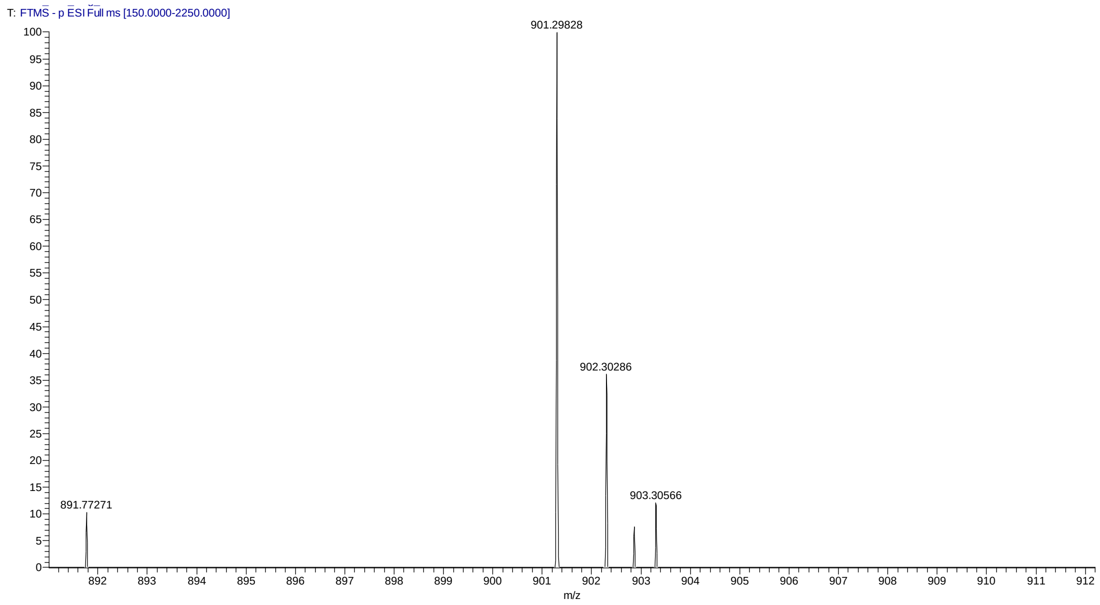

Figure S10 HRESIMS spectrum of compound **2**

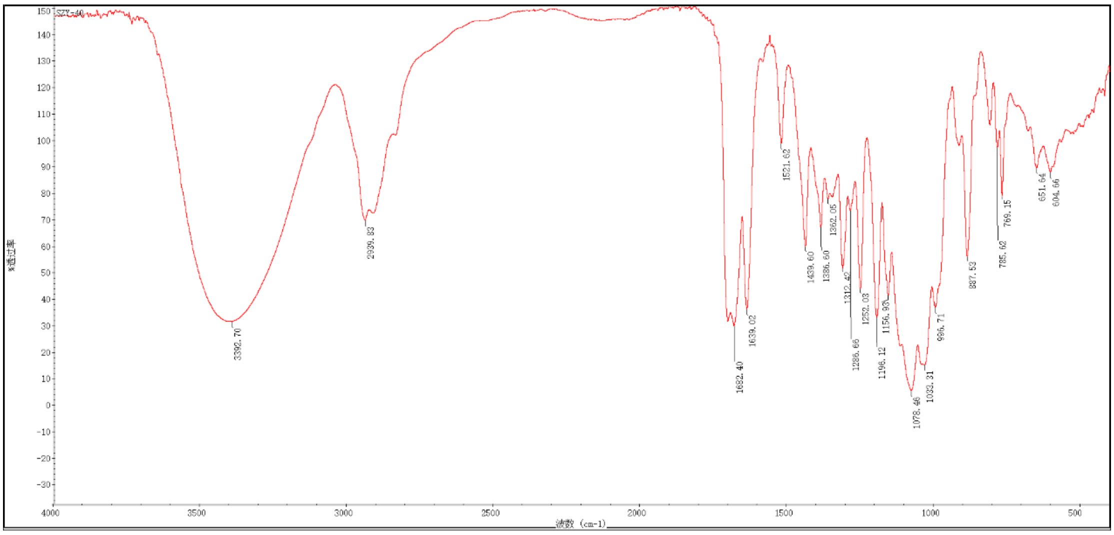

Figure S11 IR spectrum of compound **2**

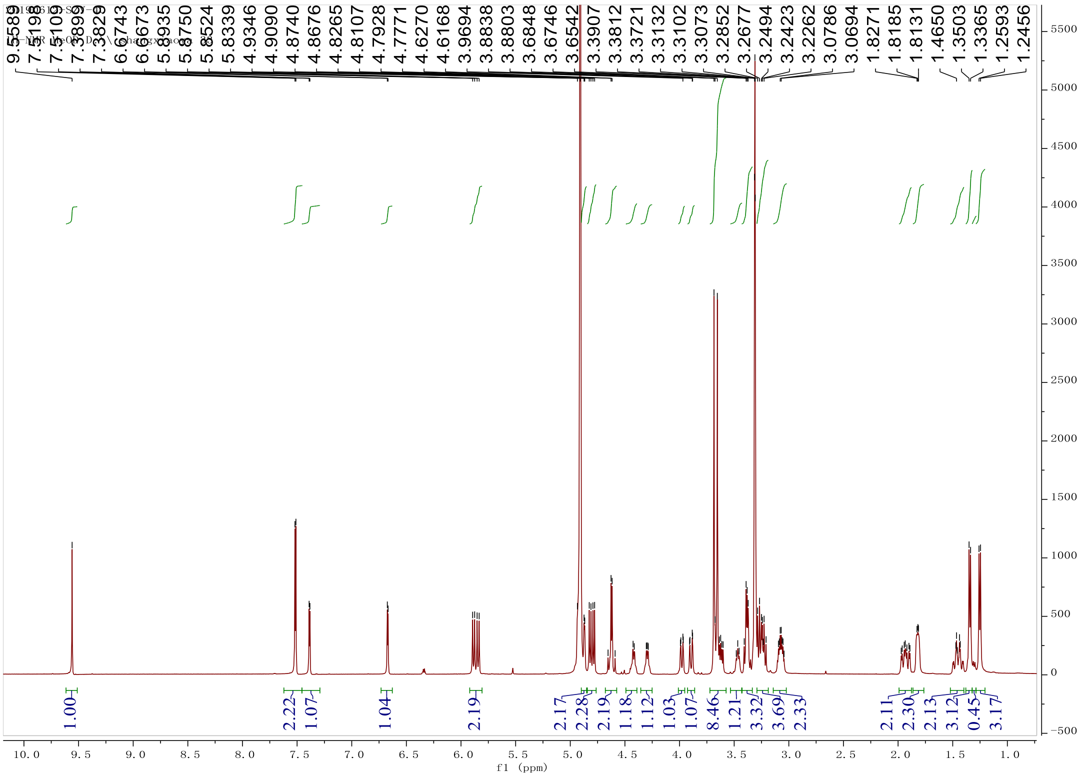

Figure S12 ^{1}H NMR spectrum of compound **2**

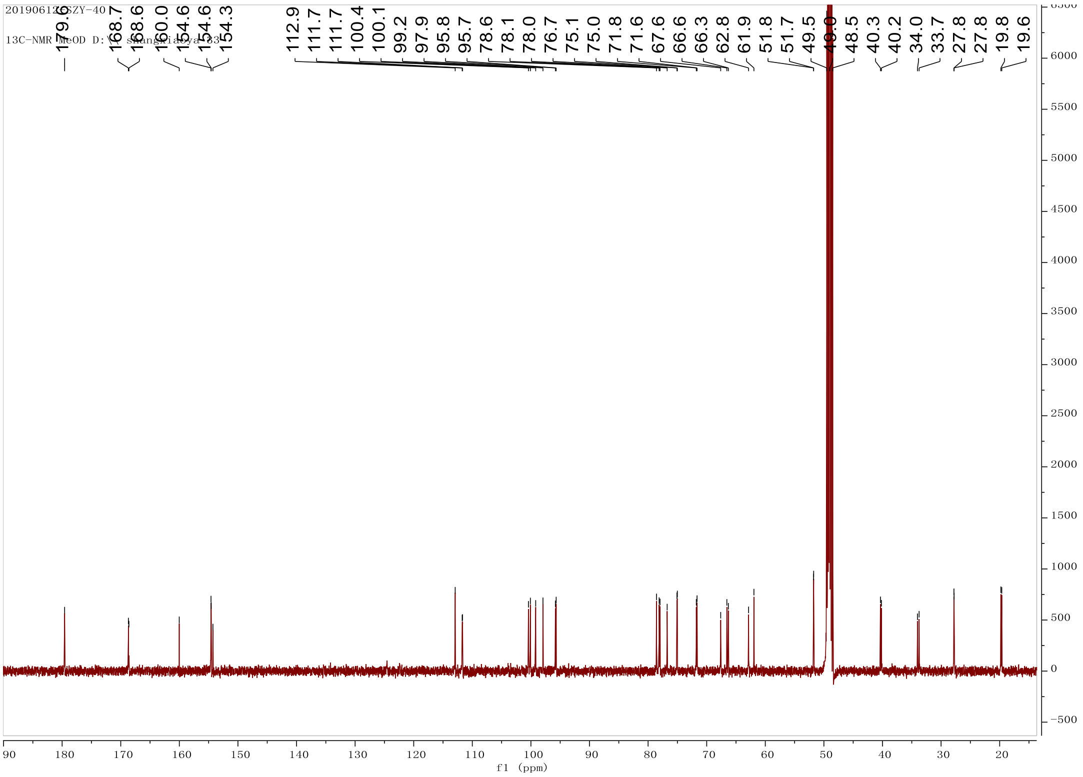

Figure S13 ^{13}C NMR spectrum of compound **2**

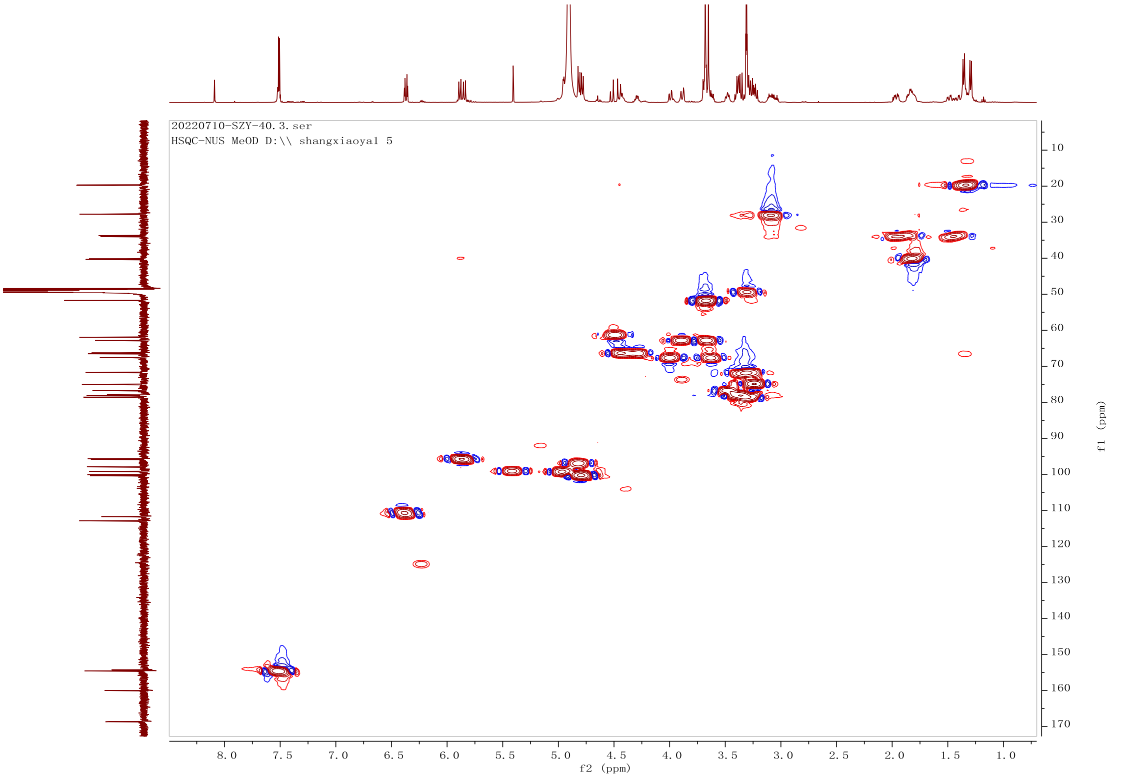

Figure S14 HSQC spectrum of compound **2**

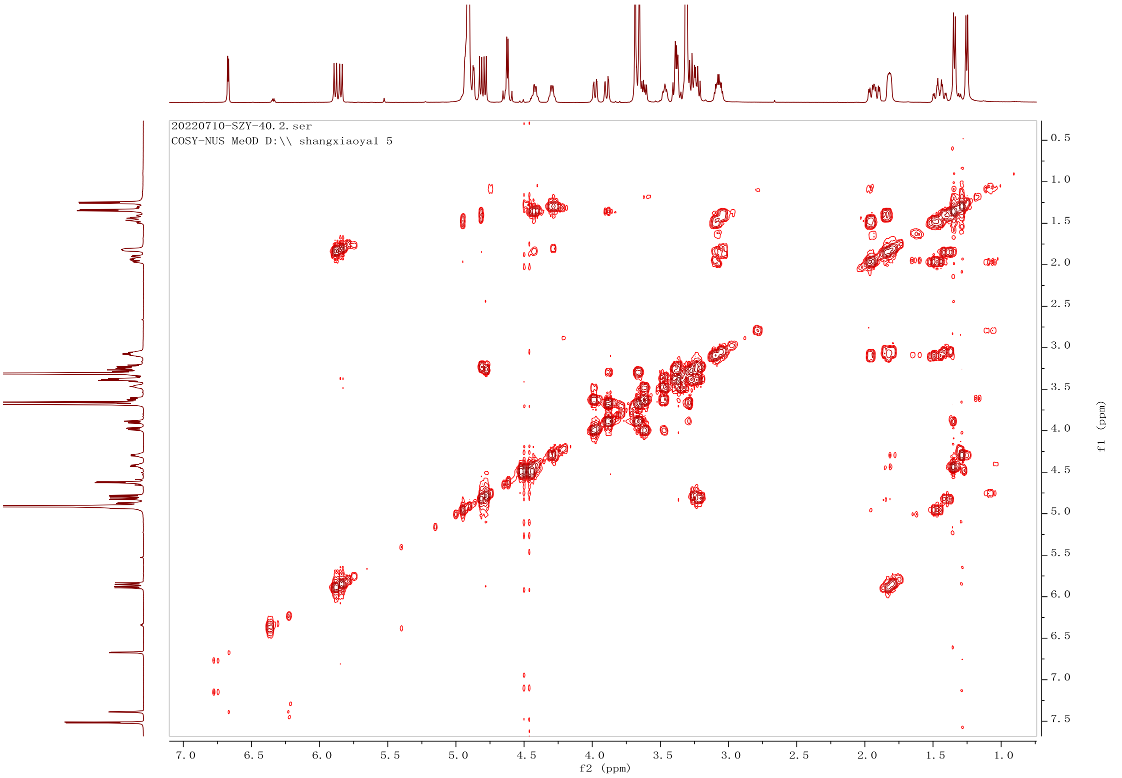

Figure S15 ^{1}H-^{1}H COSY spectrum of compound **2**

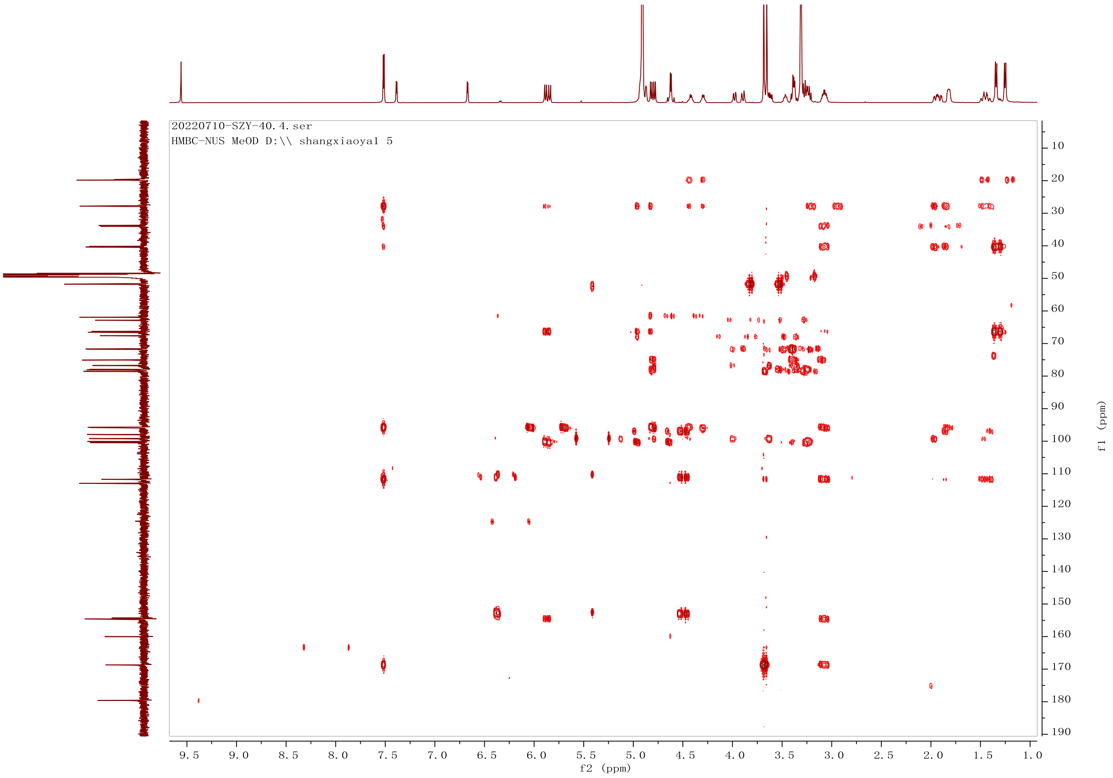

Figure S16 HMBC spectrum of compound **2**

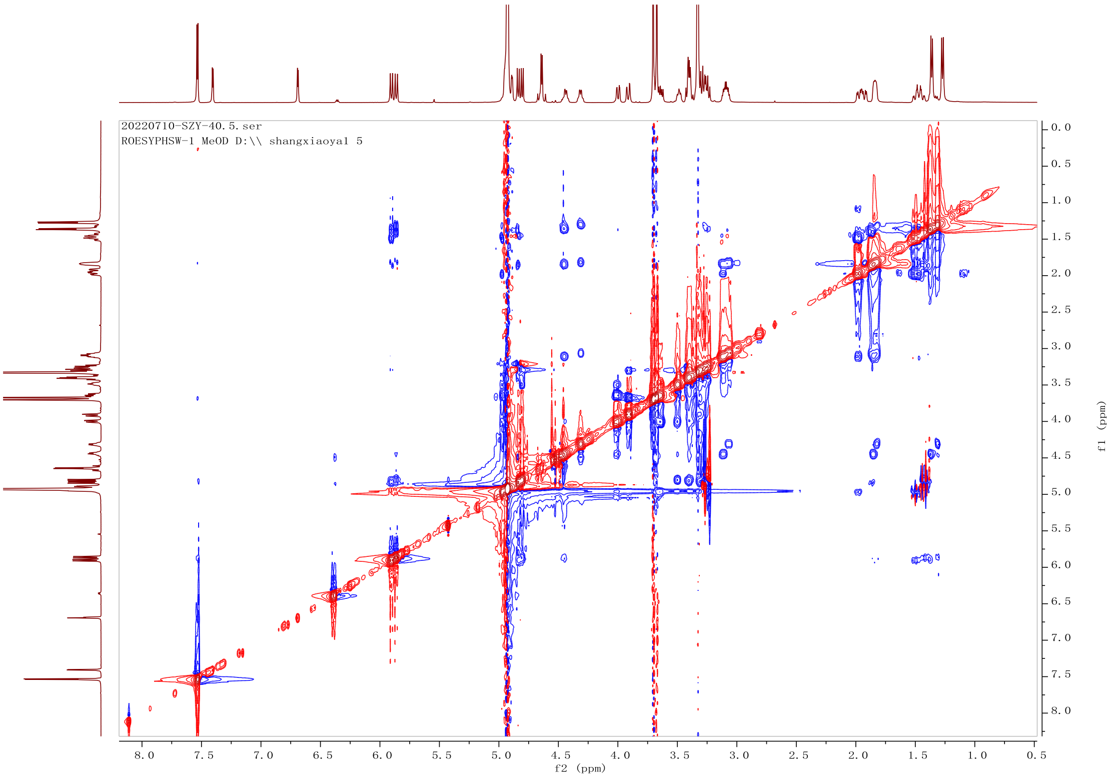

Figure S17 ROESY spectrum of compound **2**

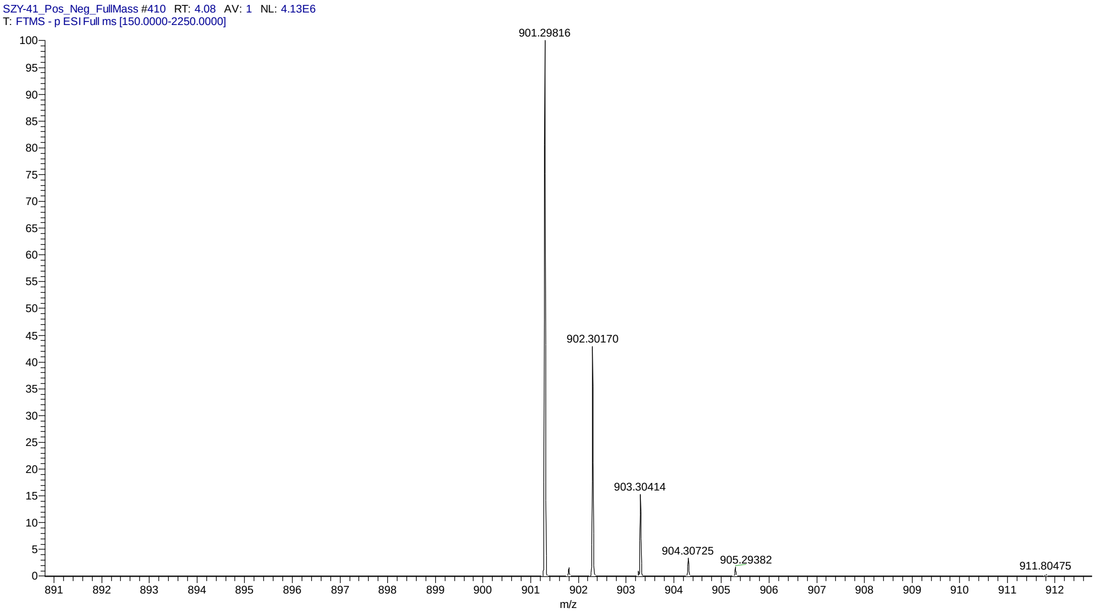

Figure S18 HRESIMS spectrum of compound **3**

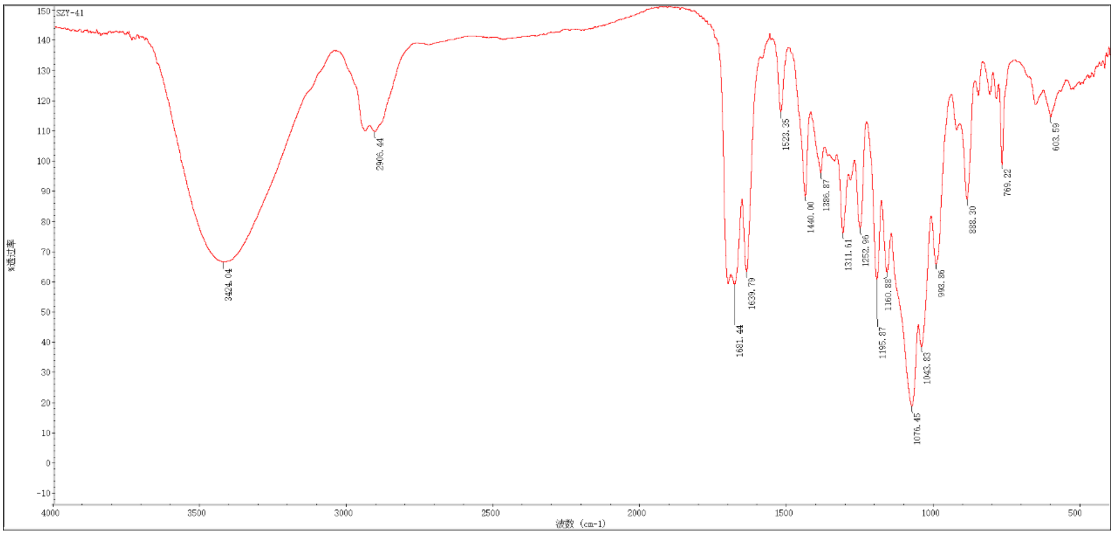

Figure S19 IR spectrum of compound **3**

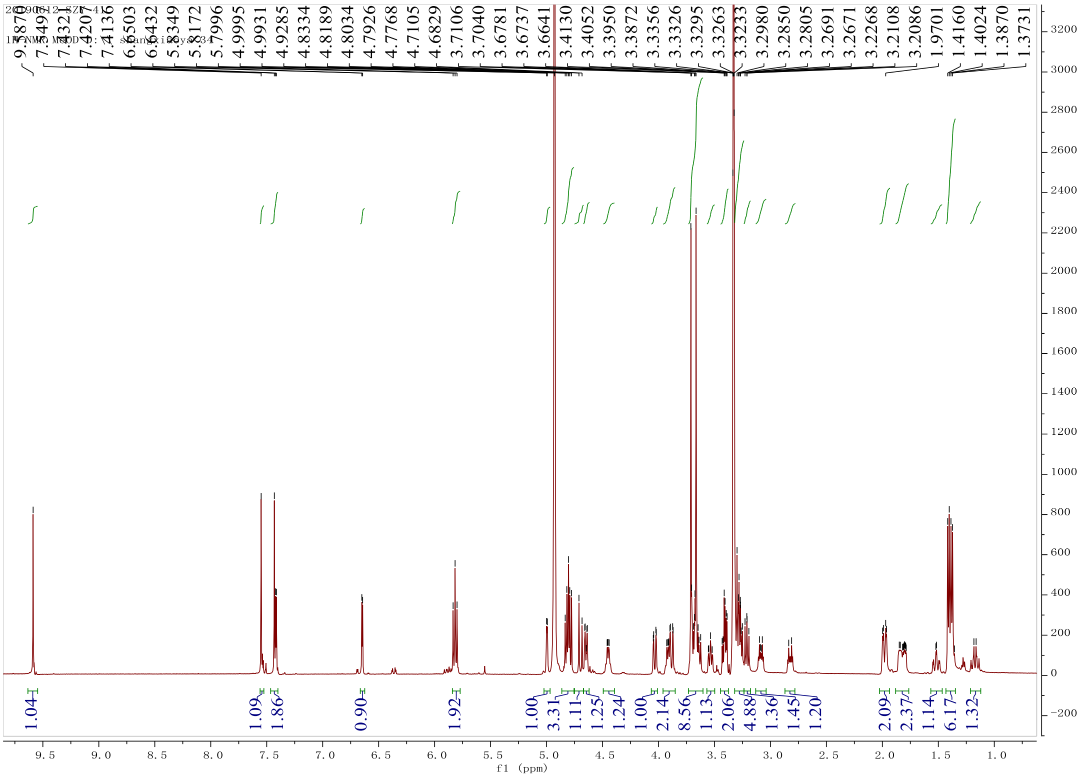

Figure S20 ^{1}H NMR spectrum of compound **3**

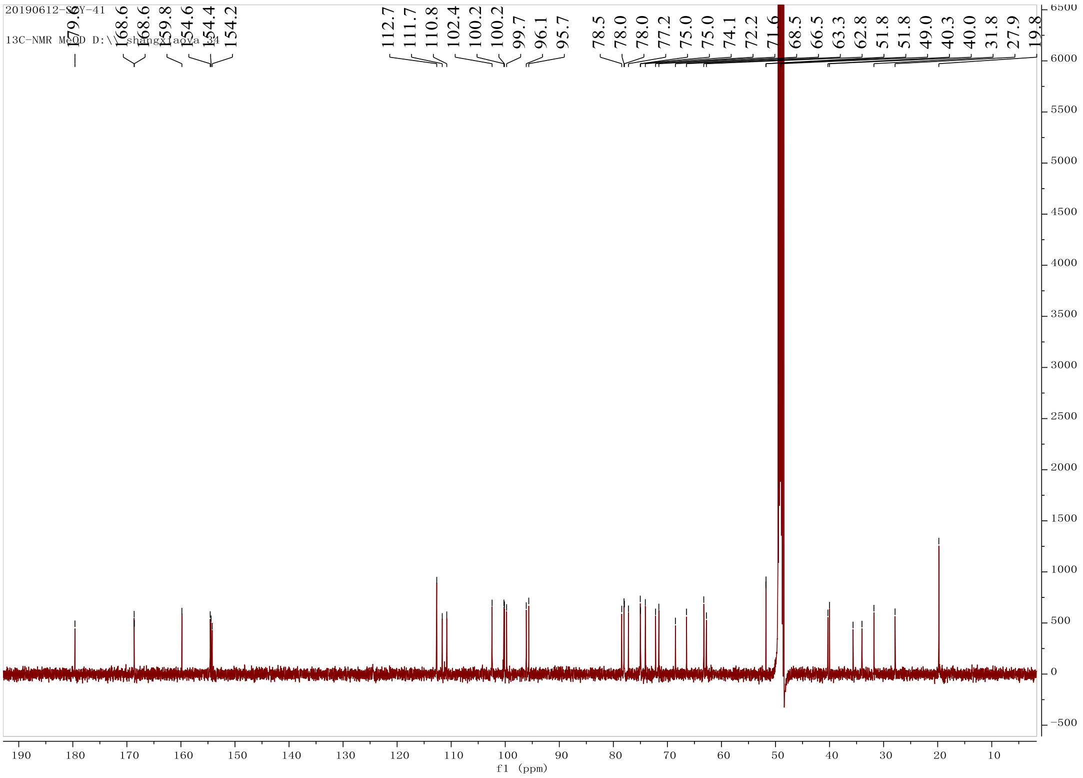

Figure S21 ^{13}C NMR spectrum of compound **3**

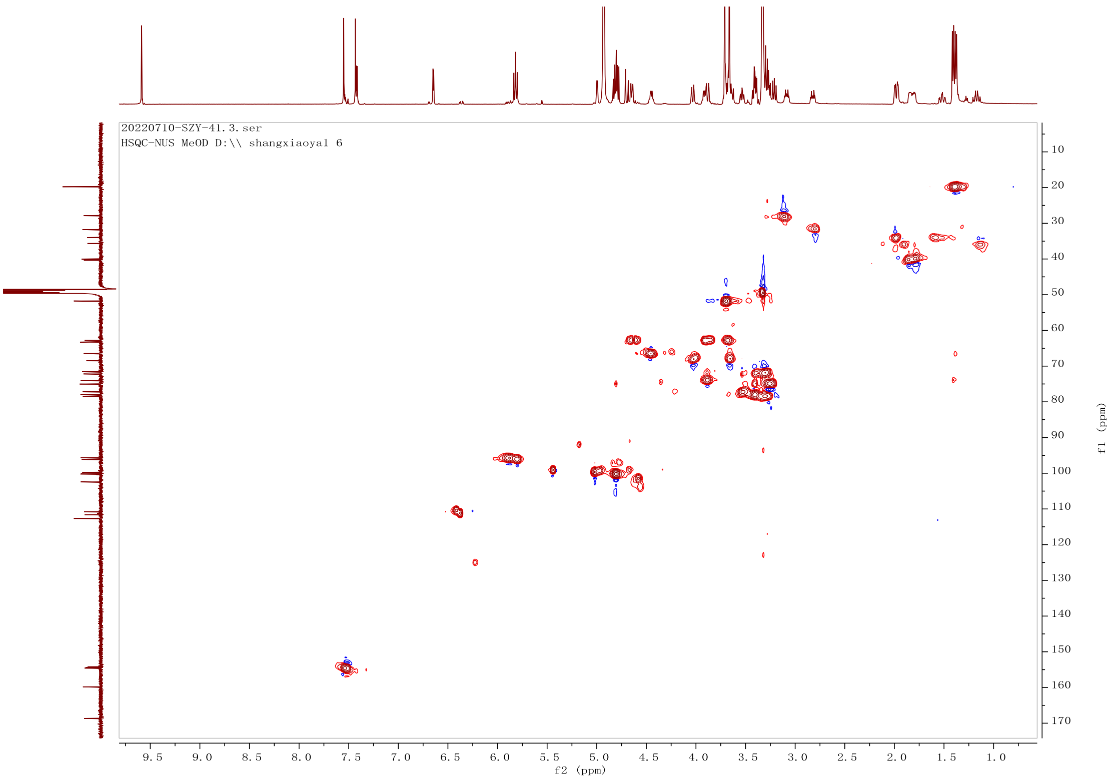

Figure S22 HSQC spectrum of compound **3**

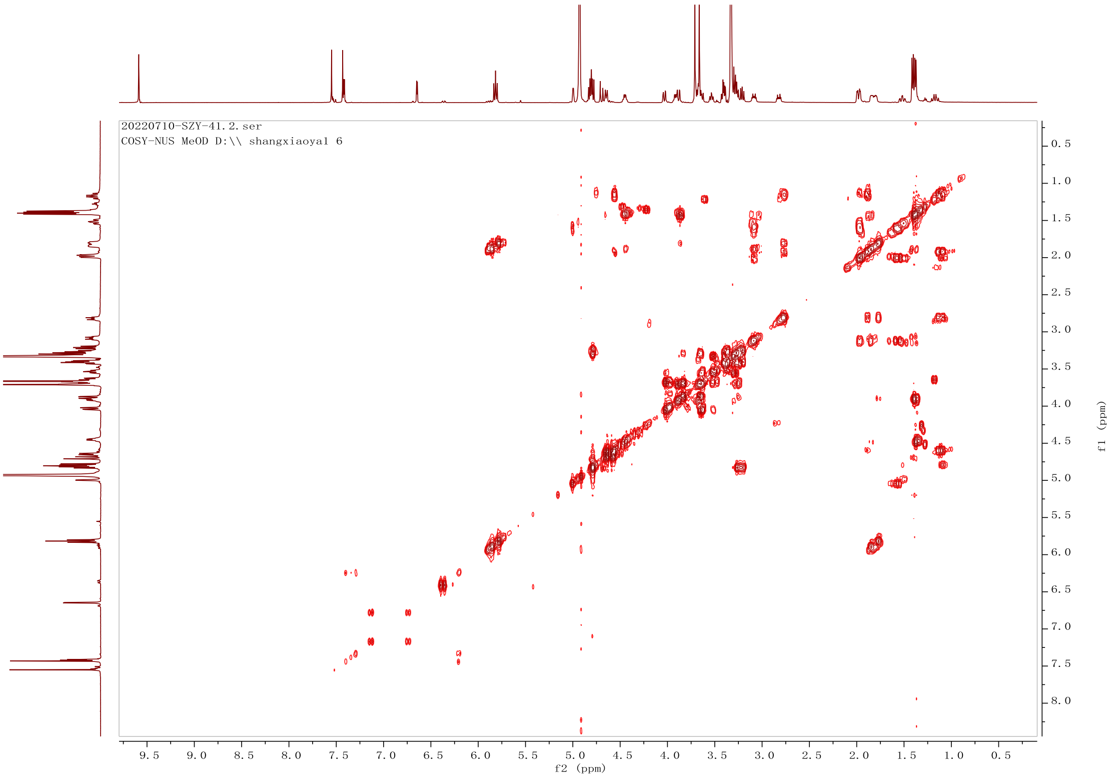

Figure S23 ^{1}H-^{1}H COSY spectrum of compound **3**

frontiers

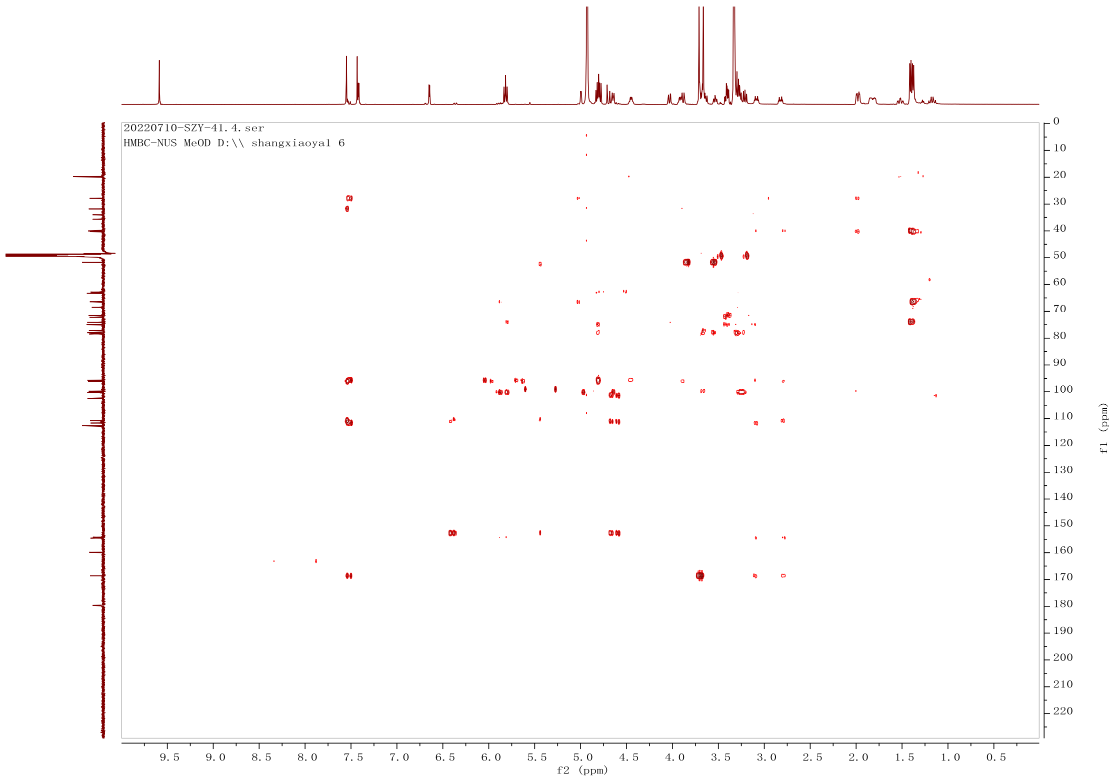

Figure S24 HMBC spectrum of compound **3**

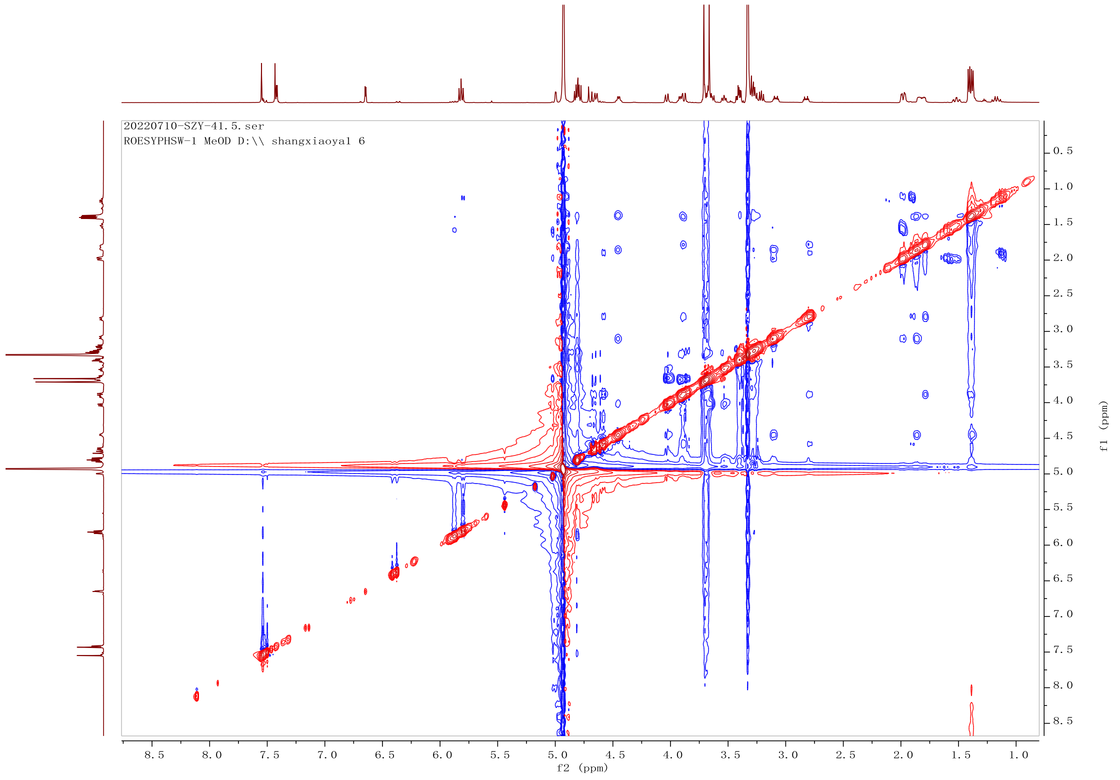

Figure S25 ROESY spectrum of compound **3**

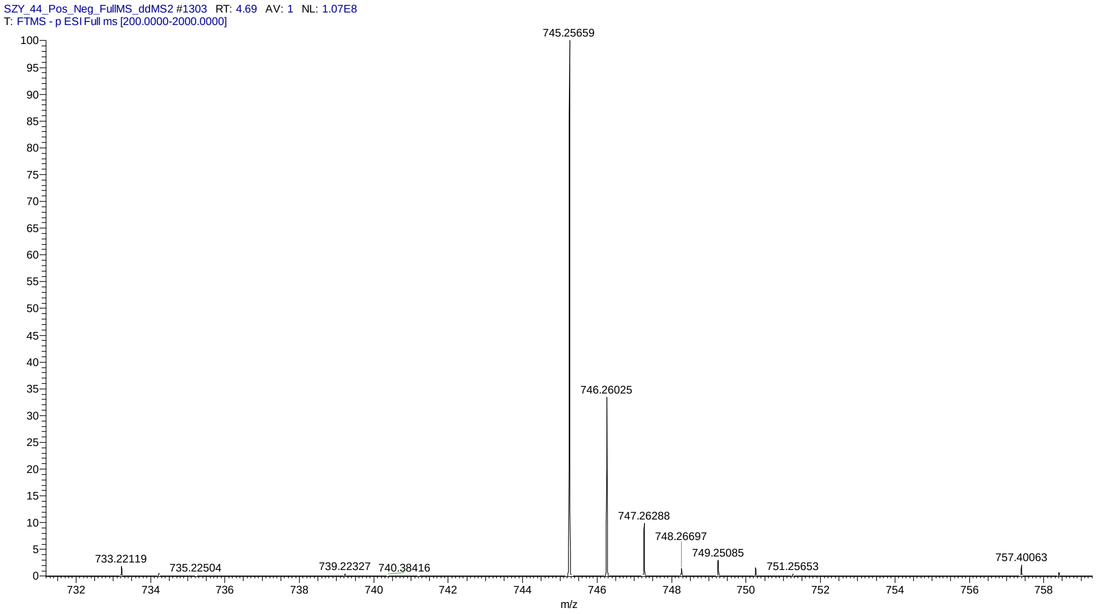

Figure S26 HRESIMS spectrum of compound **4**

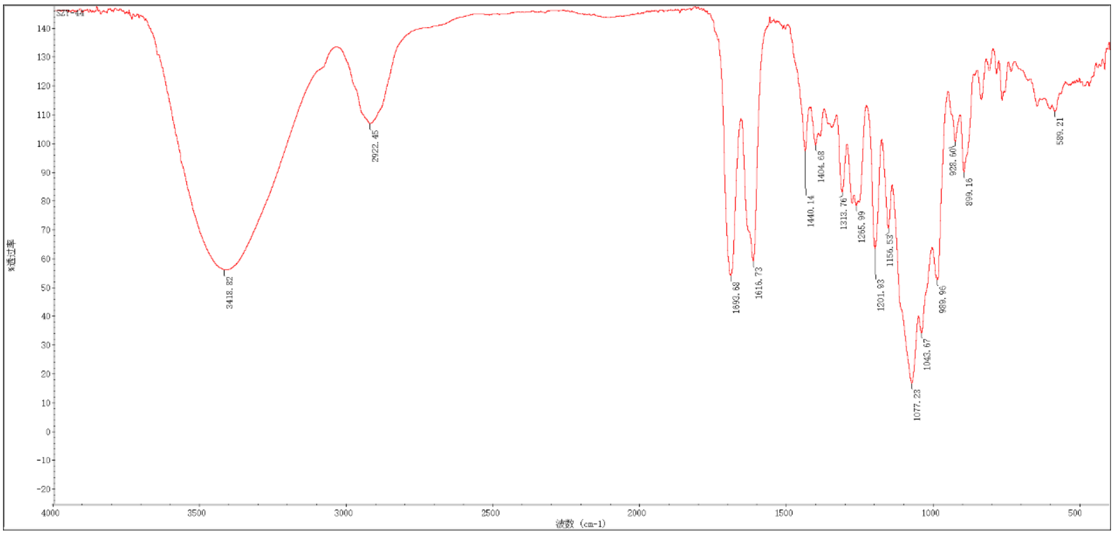

Figure S27 IR spectrum of compound **4**

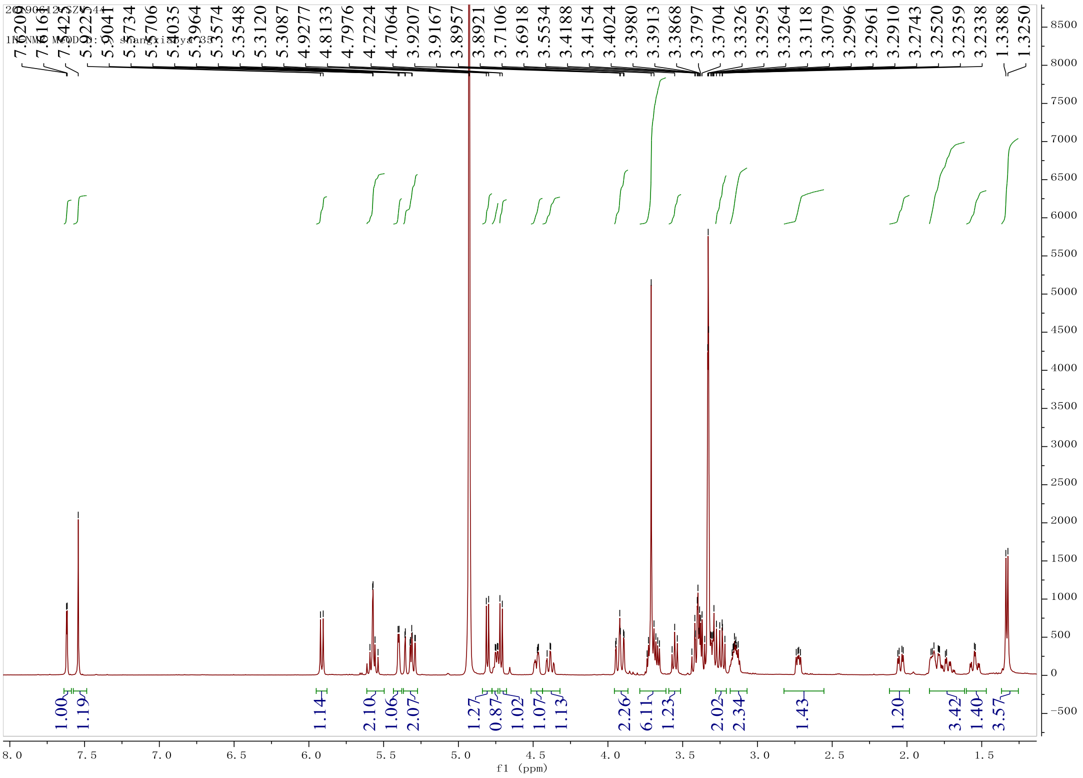

Figure S28 ^{1}H NMR spectrum of compound **4**

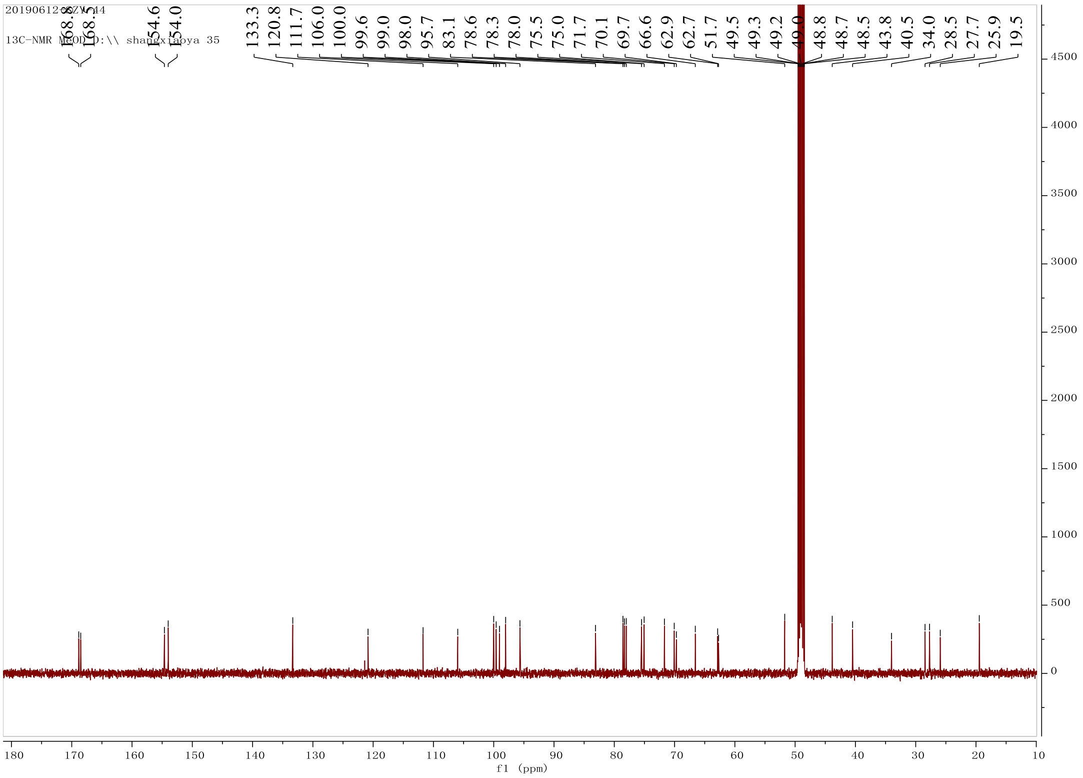

Figure S29 ^{13}C NMR spectrum of compound **4**

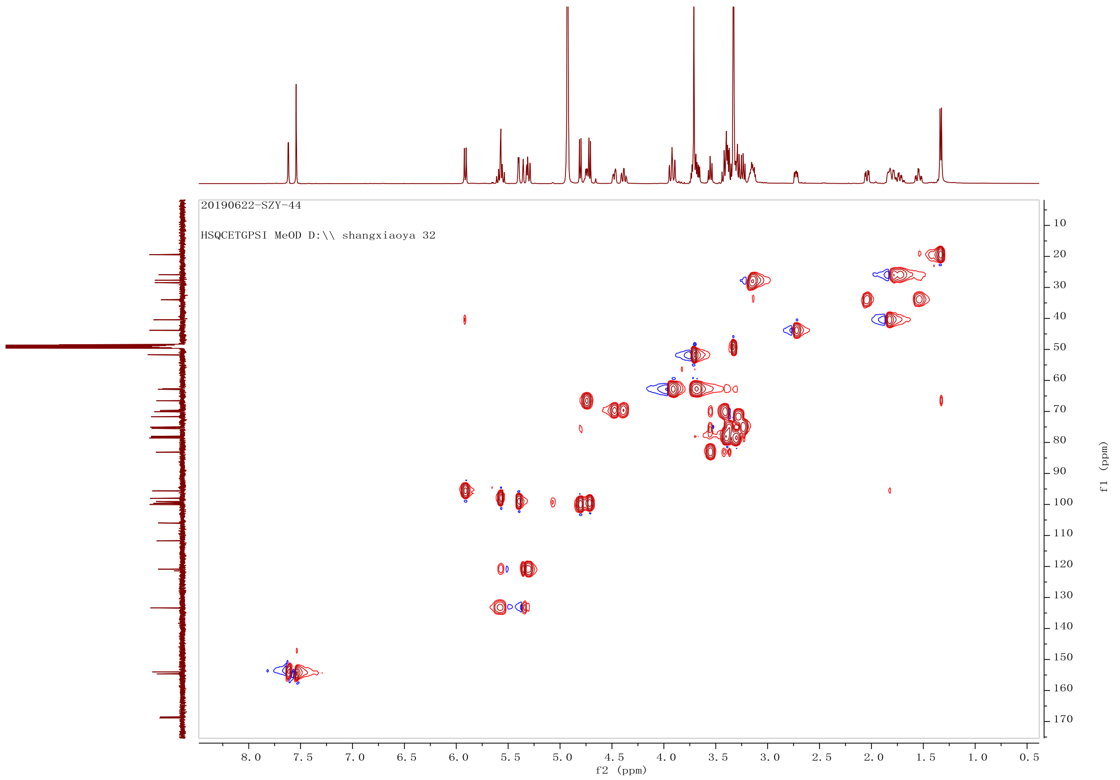

Figure S30 HSQC spectrum of compound **4**

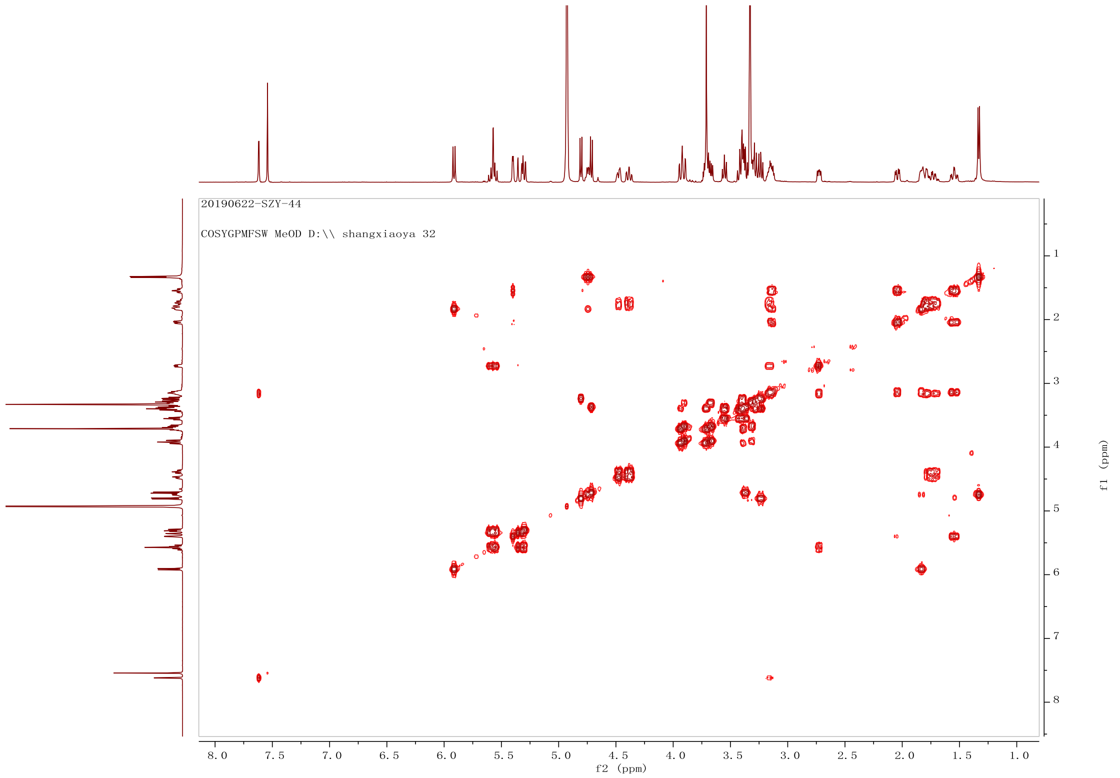

Figure S31 ^{1}H-^{1}H COSY spectrum of compound **4**

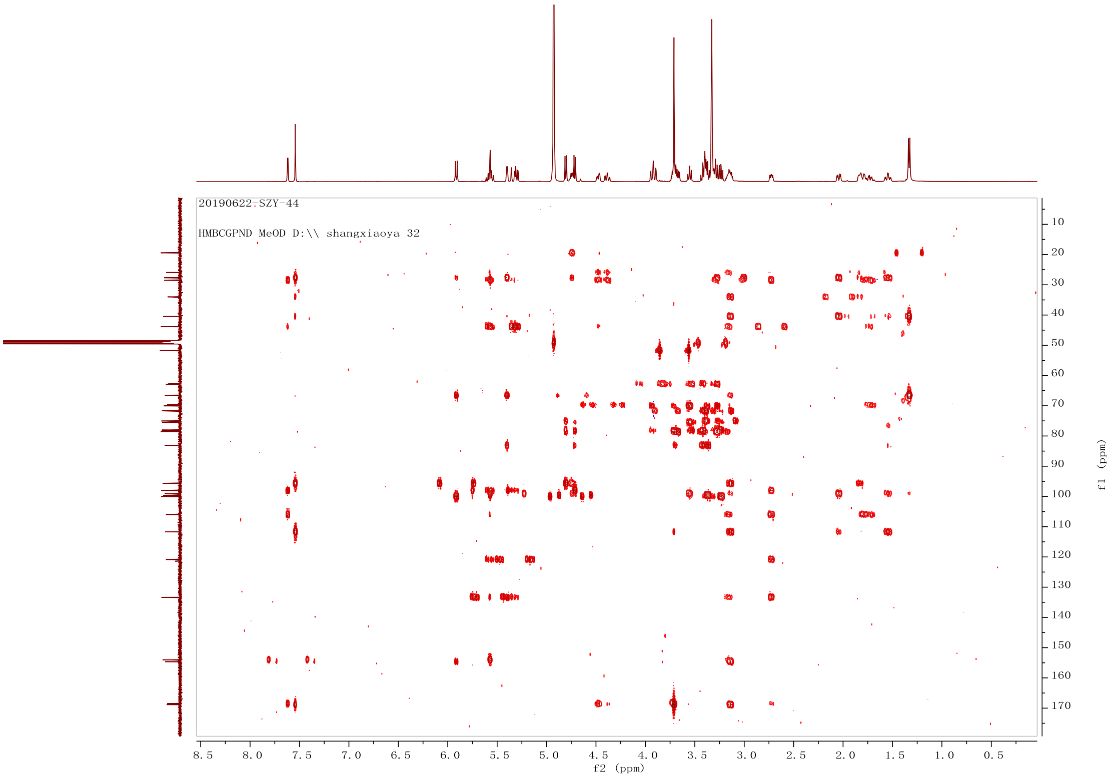

Figure S32 HMBC spectrum of compound **4**

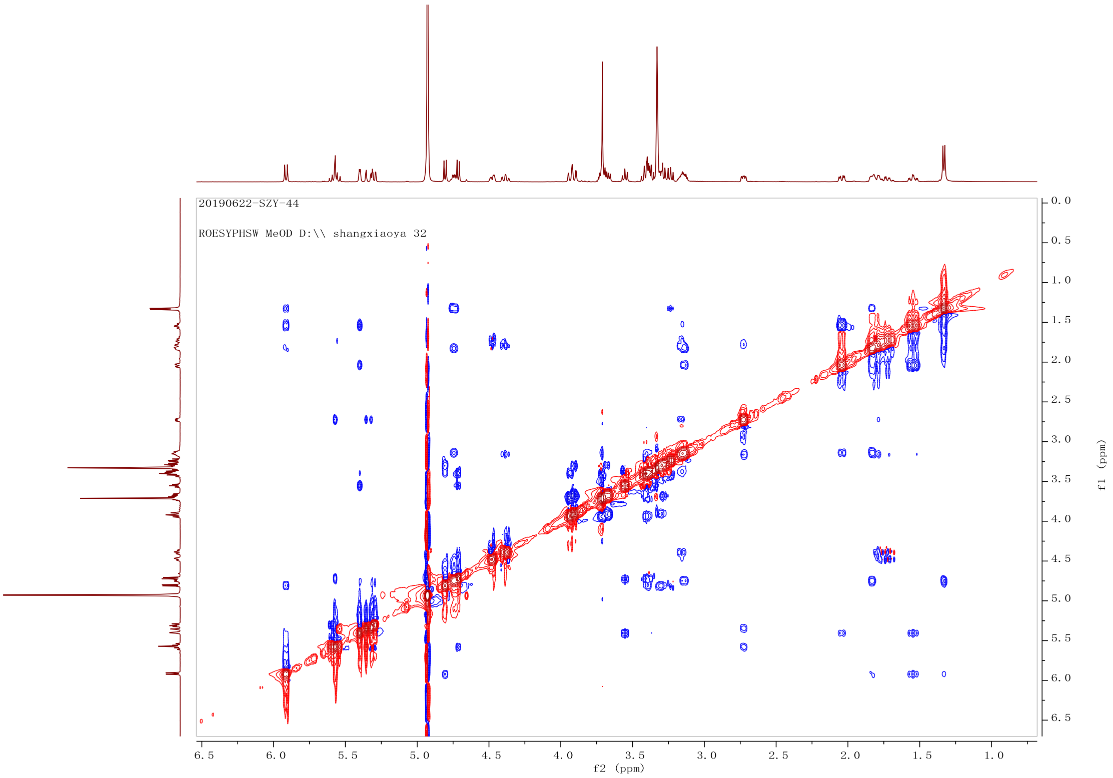

Figure S33 ROESY spectrum of compound **4**

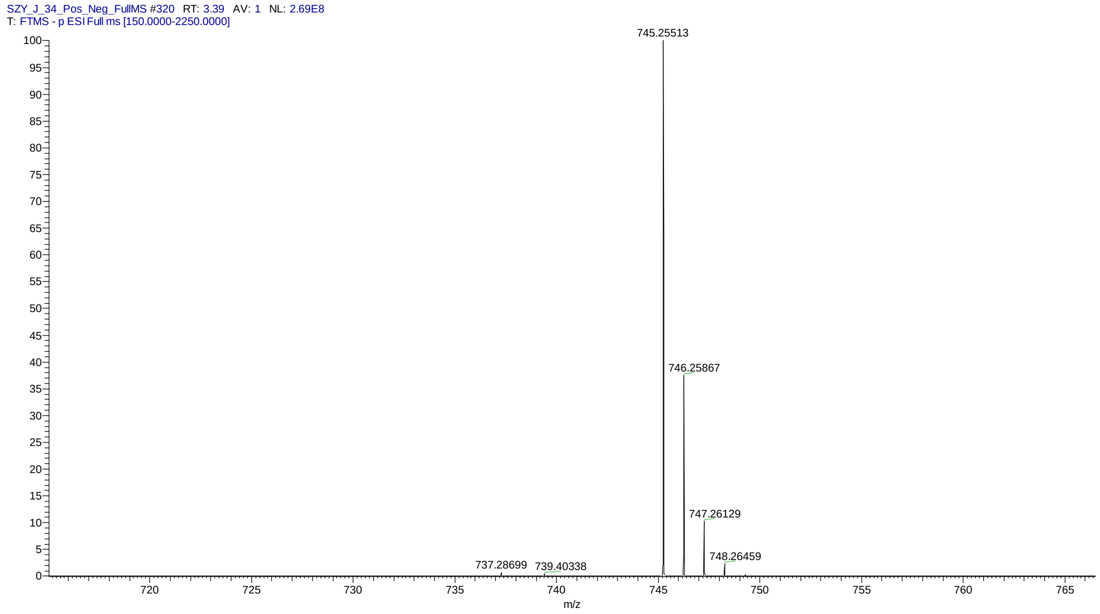

Figure S34 HRESIMS spectrum of compound **5**

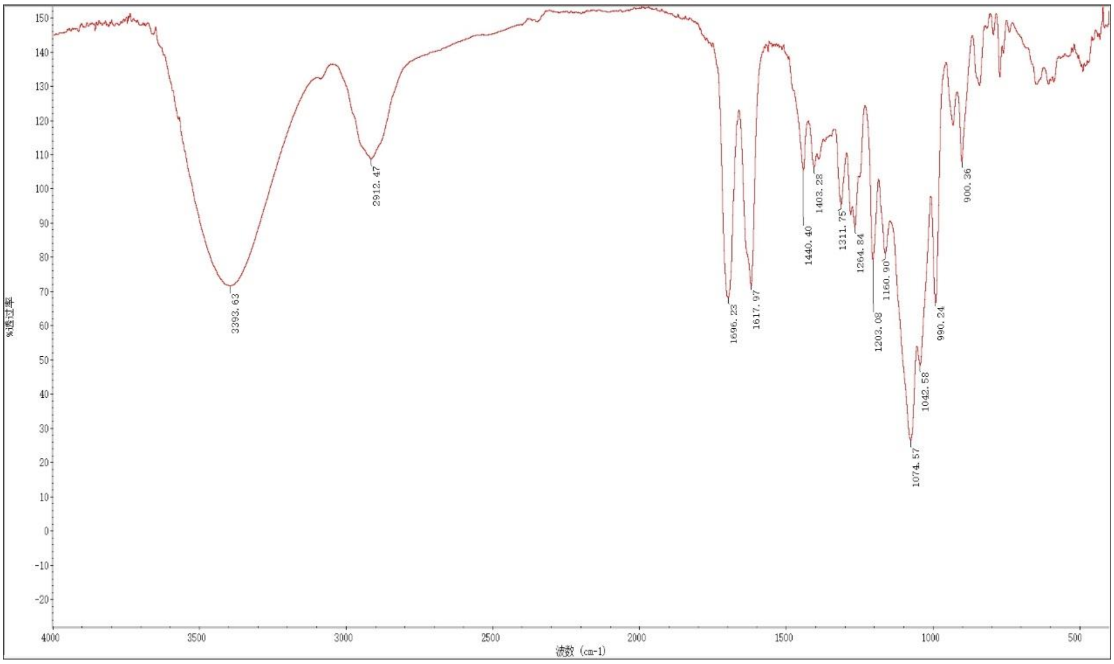

Figure S35 IR spectrum of compound **5**

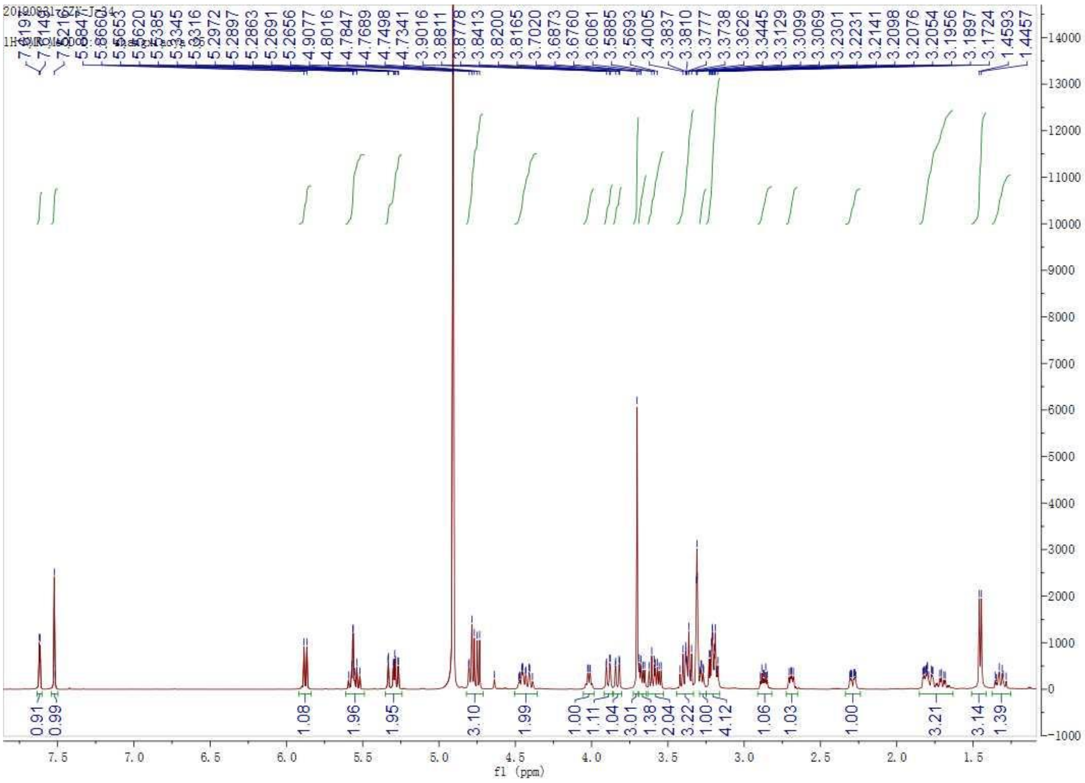

Figure S36 ^{1}H NMR spectrum of compound **5**

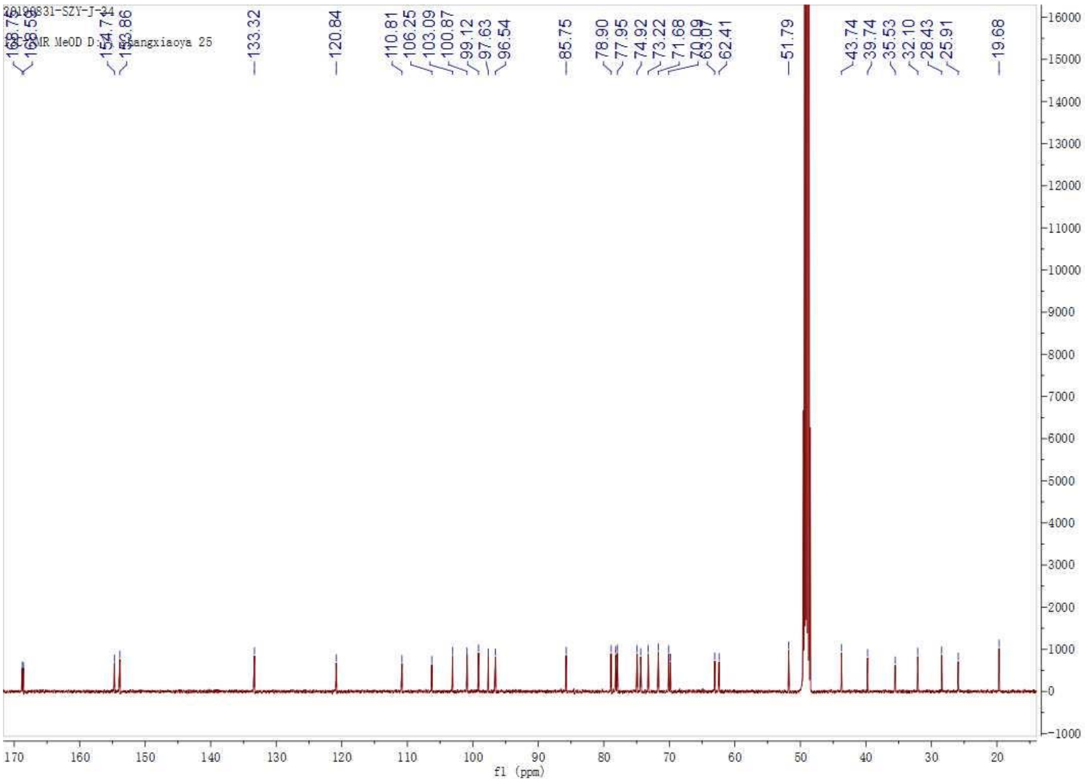

Figure S37 ^{13}C NMR spectrum of compound **5**

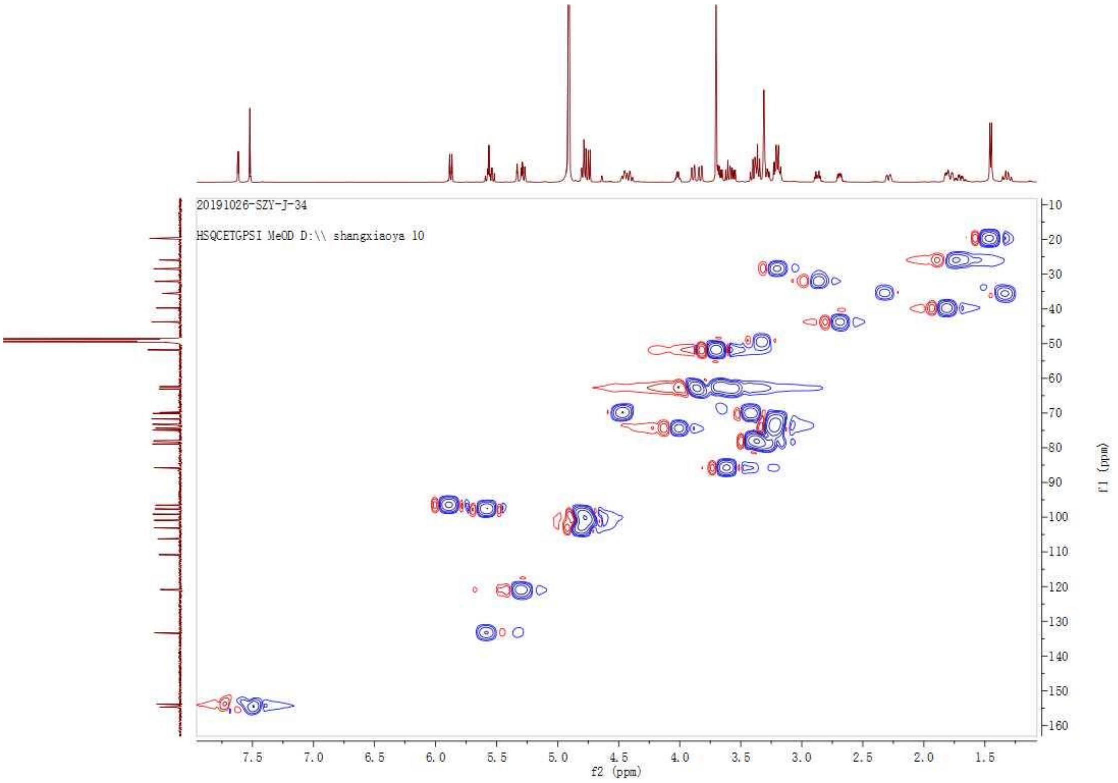

Figure S38 HSQC spectrum of compound **5**

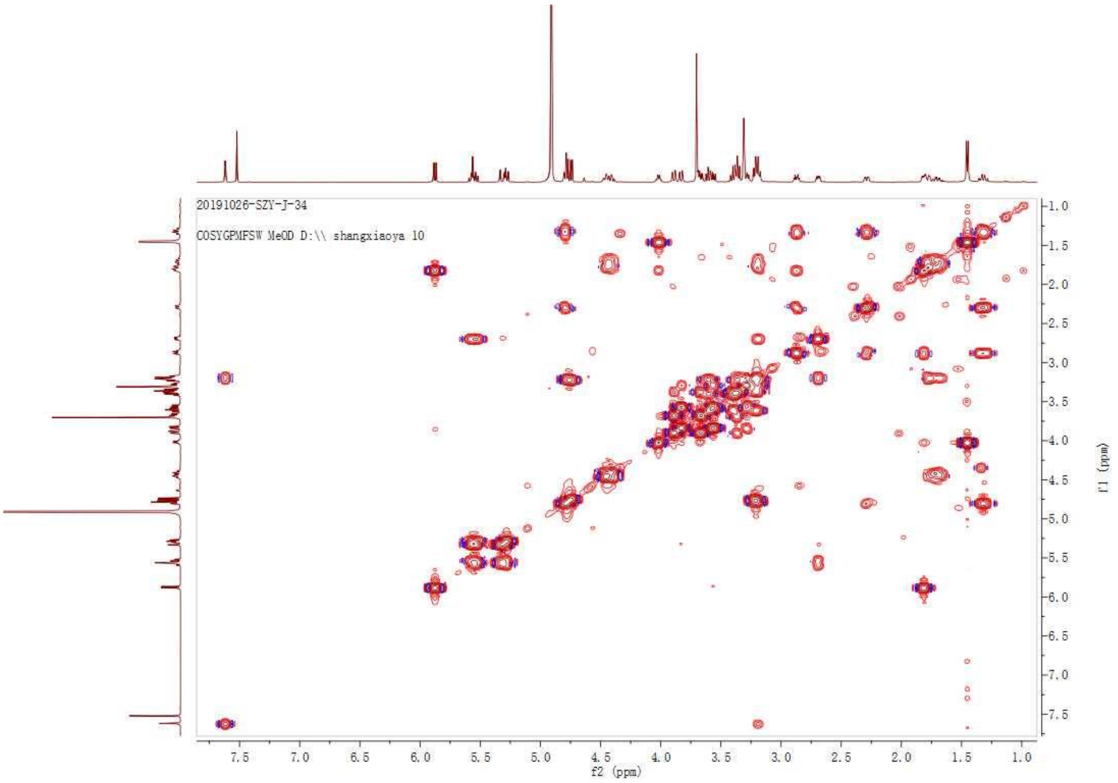

Figure S39 ^{1}H-^{1}H COSY spectrum of compound **5**

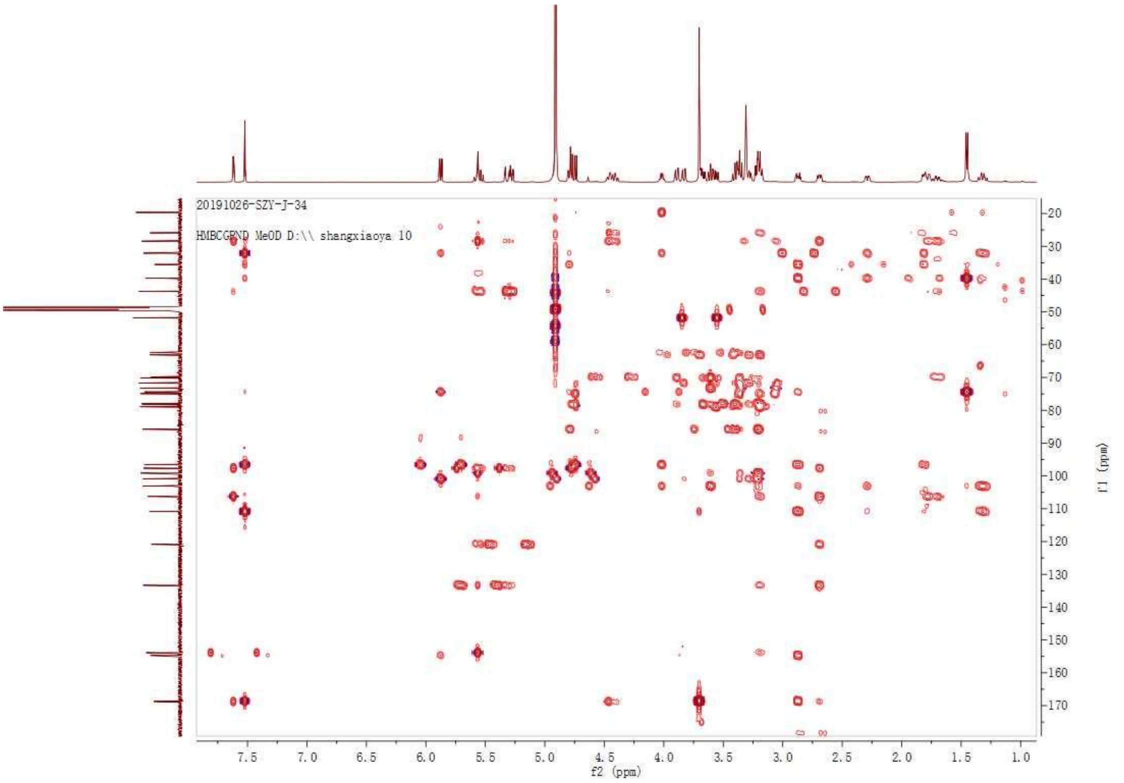

Figure S40 HMBC spectrum of compound **5**

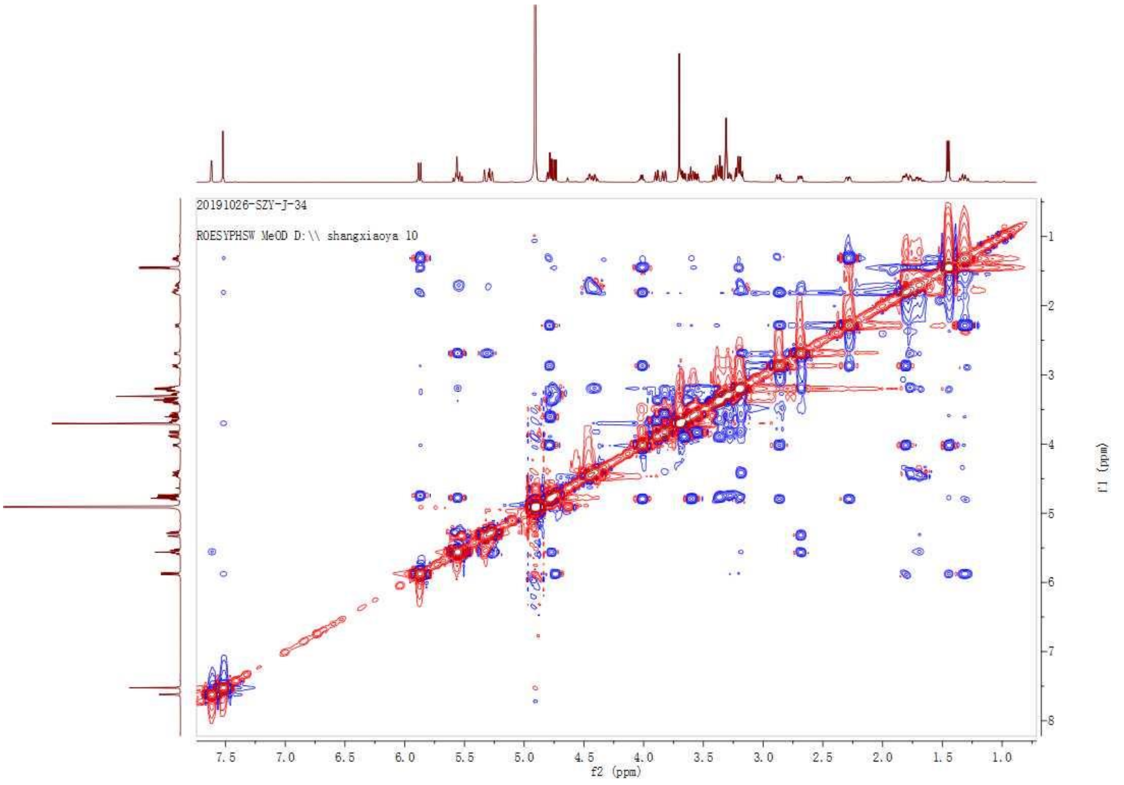

Figure S41 ROESY spectrum of compound **5**

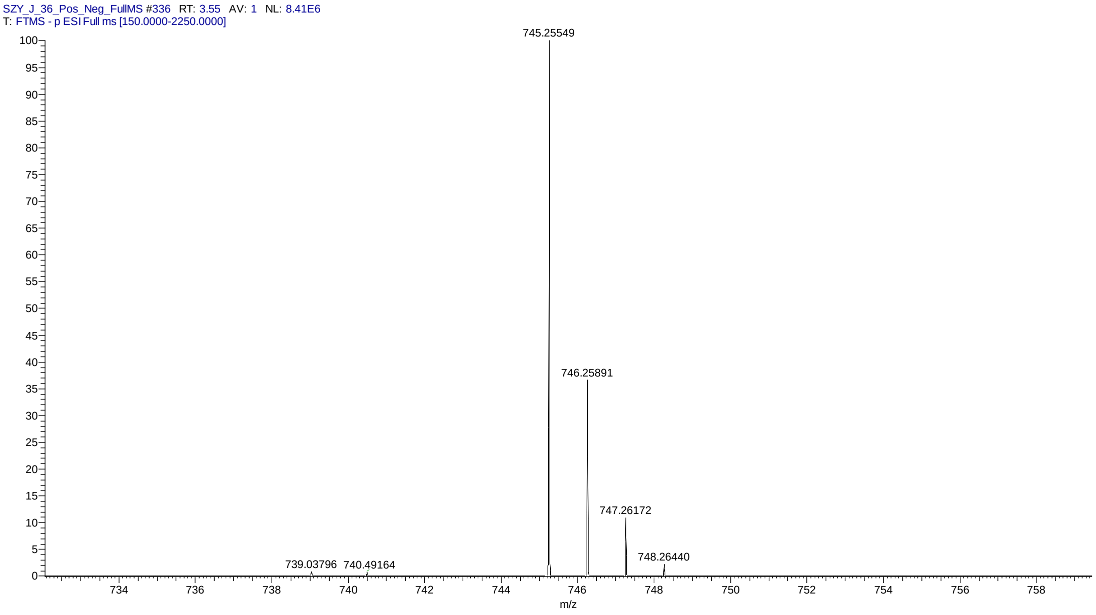

Figure S42 HRESIMS spectrum of compound **6**

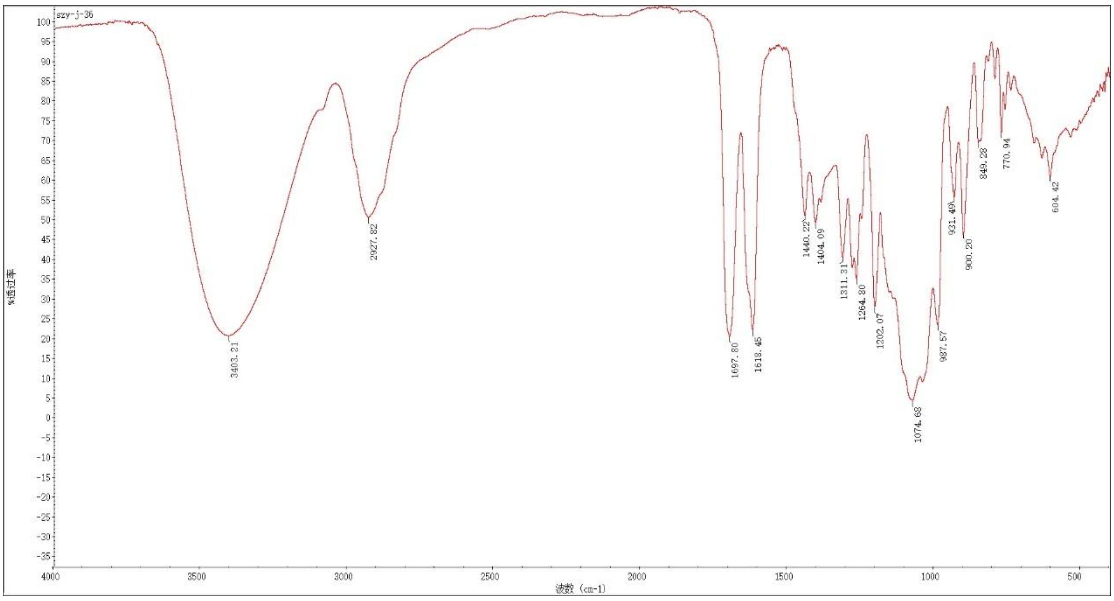

Figure S43 IR spectrum of compound **6**

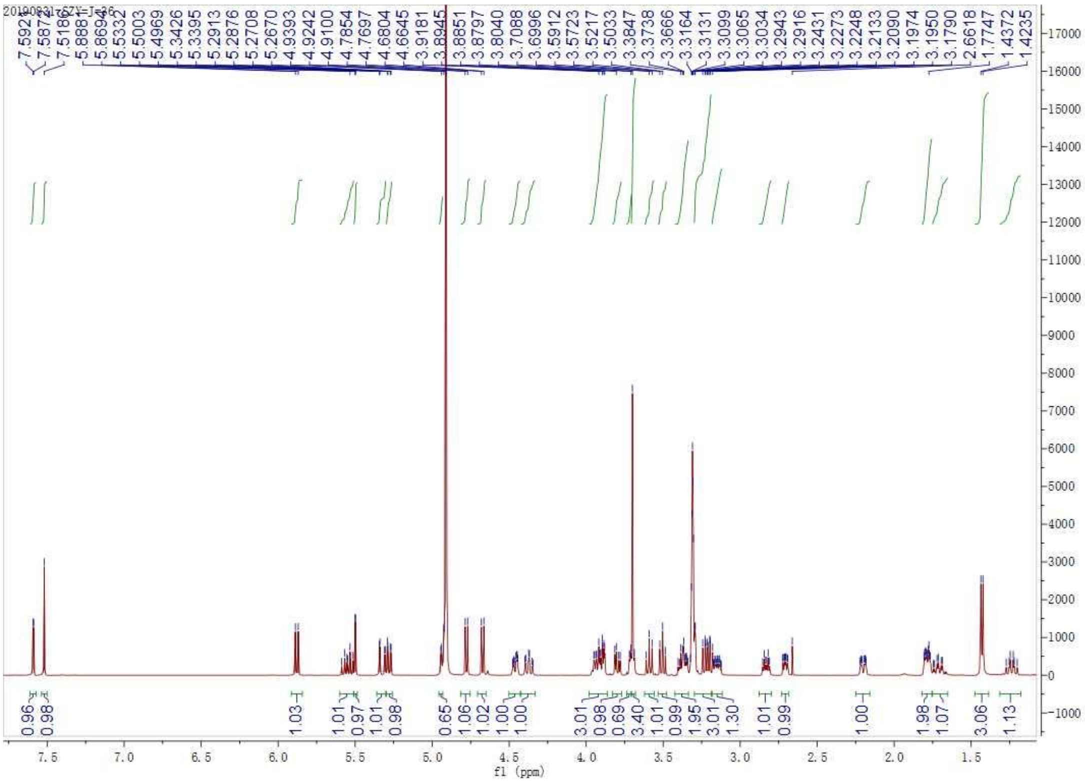

Figure S44 ^{1}H NMR spectrum of compound **6**

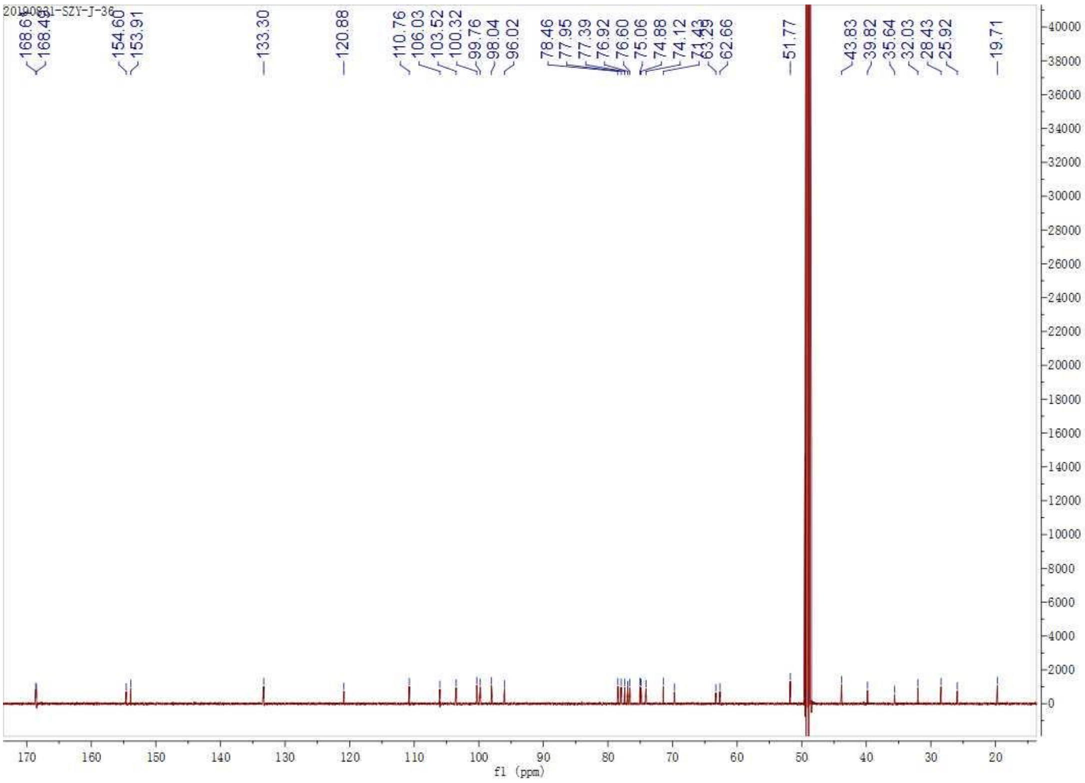

Figure S45 ^{13}C NMR spectrum of compound **6**

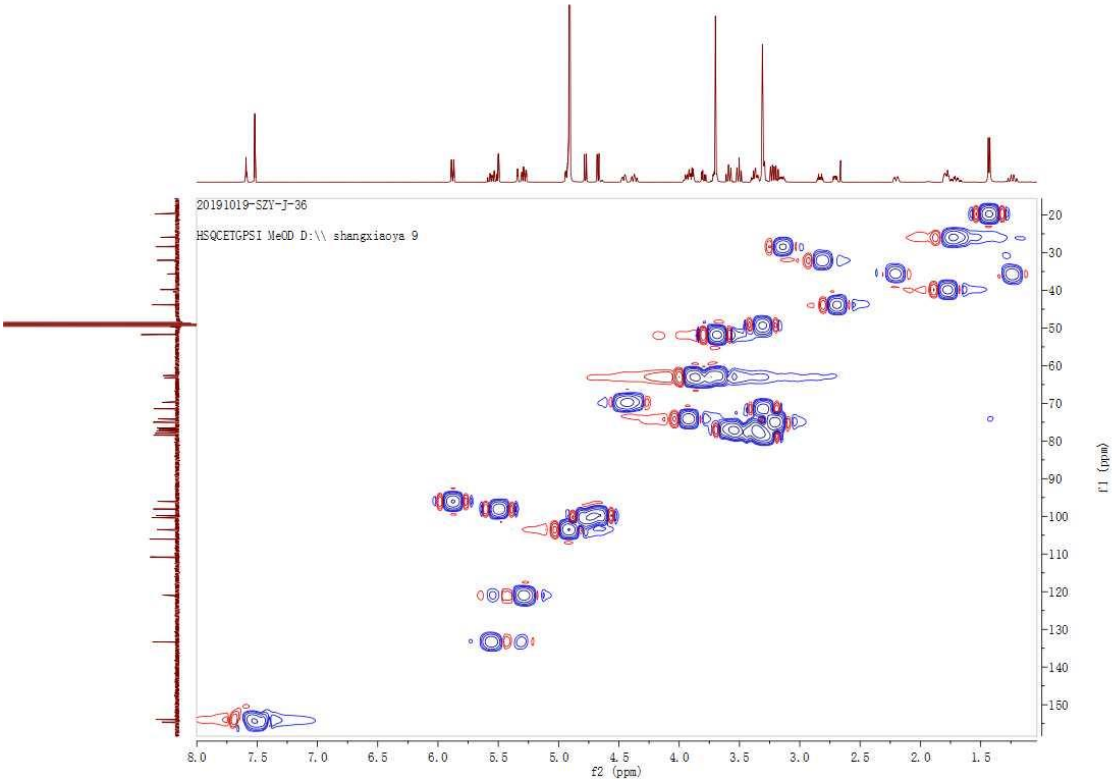

Figure S46 HSQC spectrum of compound **6**

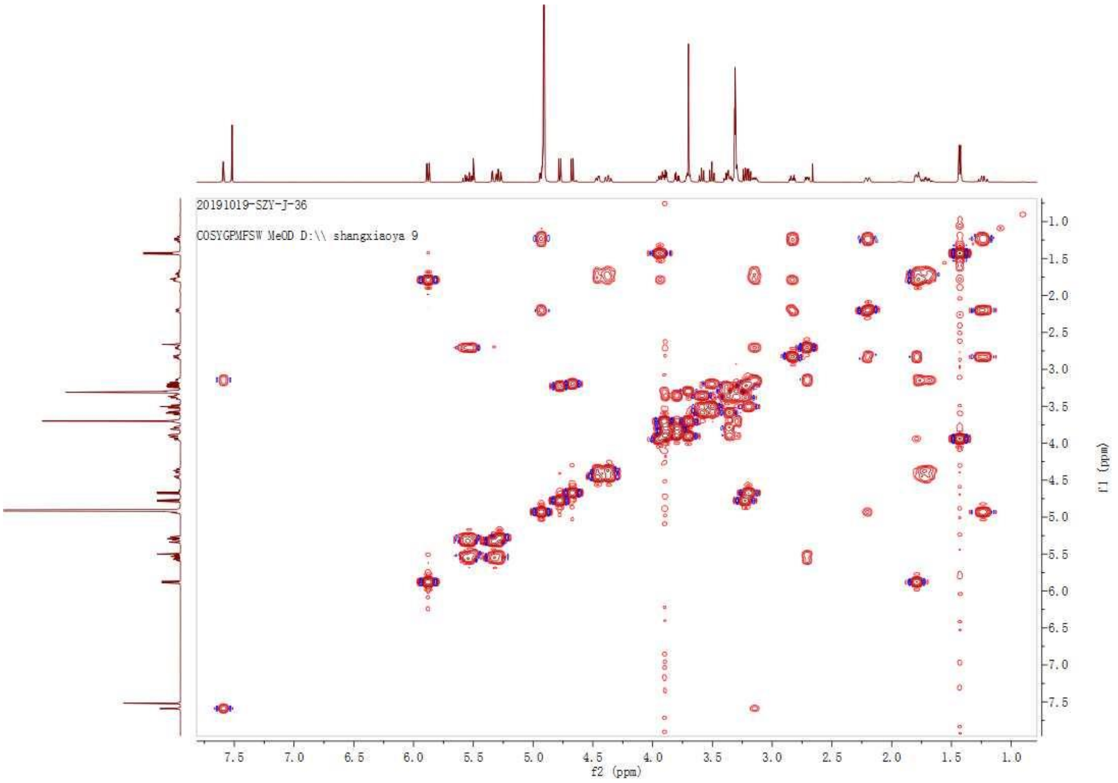

Figure S47 ^{1}H-^{1}H COSY spectrum of compound **6**

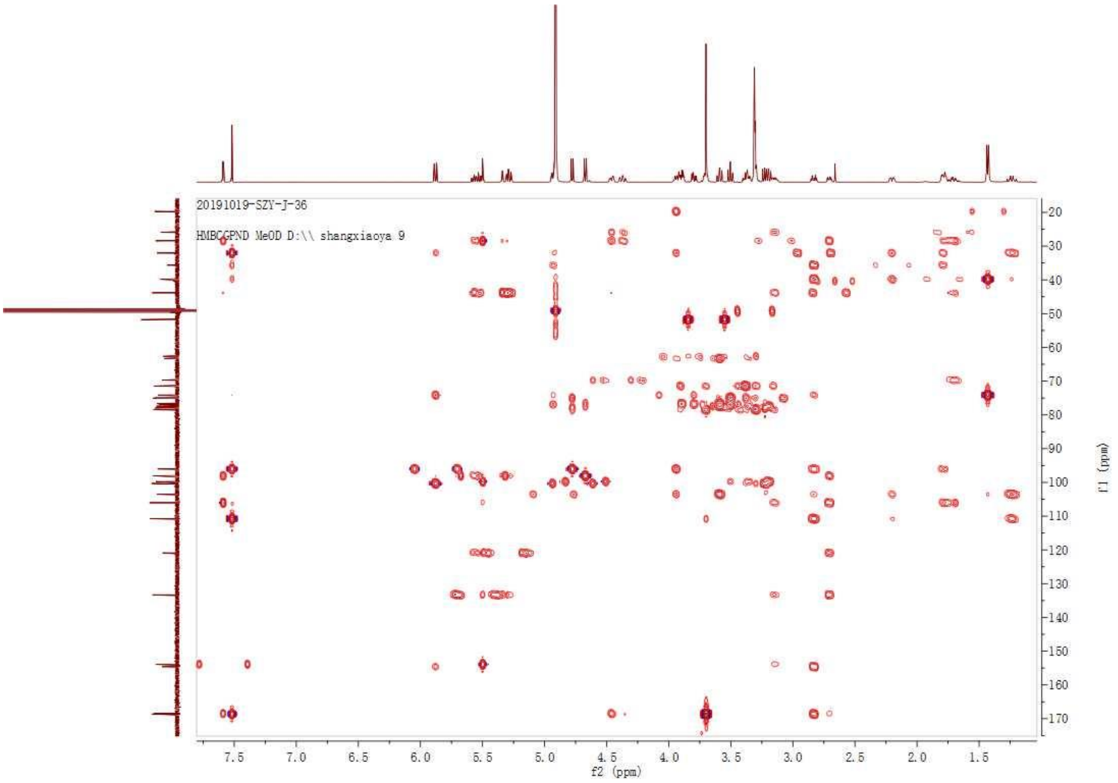

Figure S48 HMBC spectrum of compound **6**

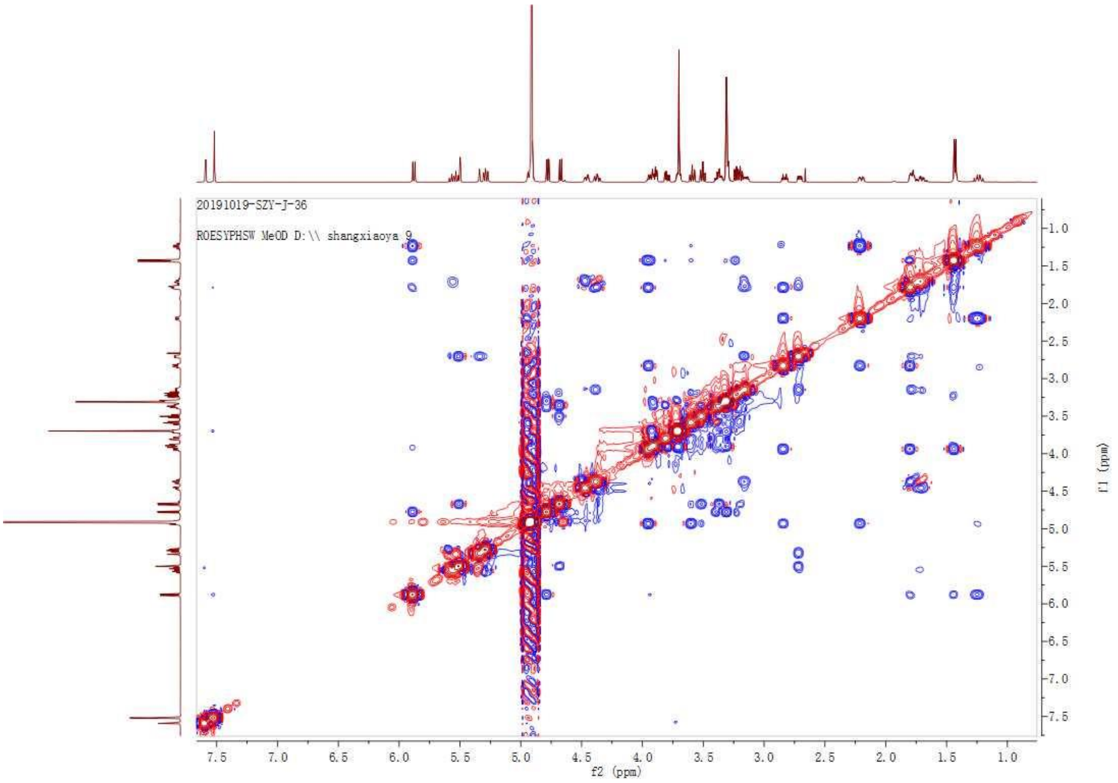

Figure S49 ROESY spectrum of compound **6**

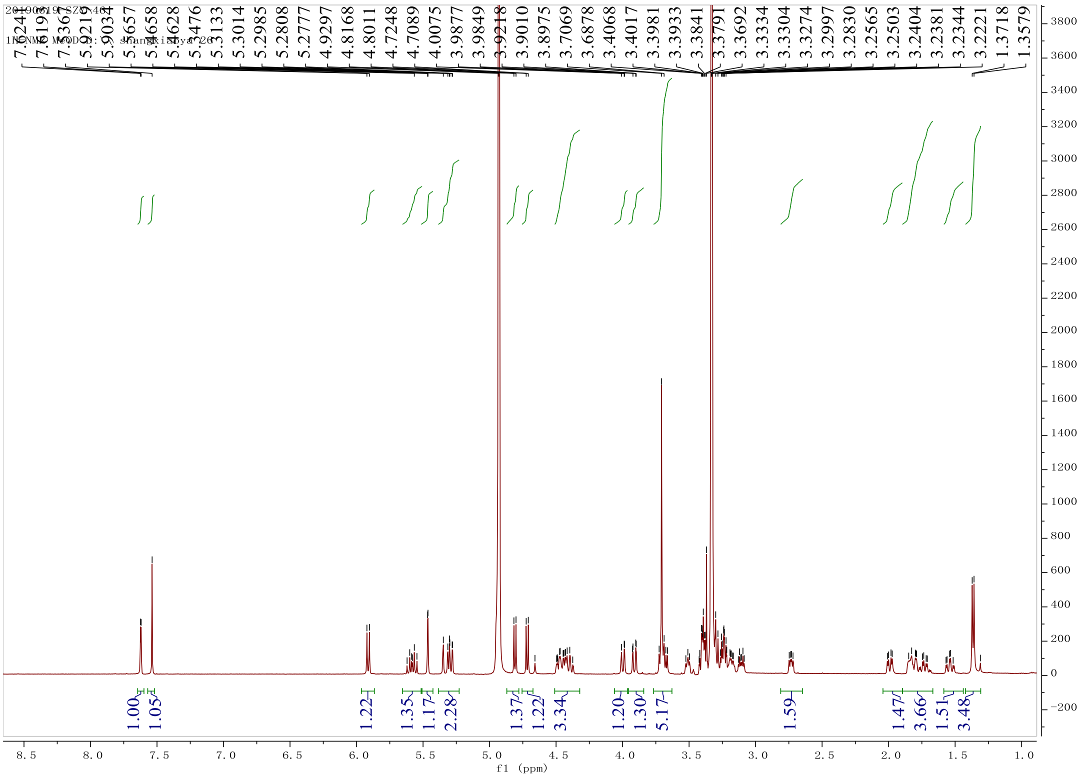

Figure S50 ^{1}H NMR spectrum of compound **7**

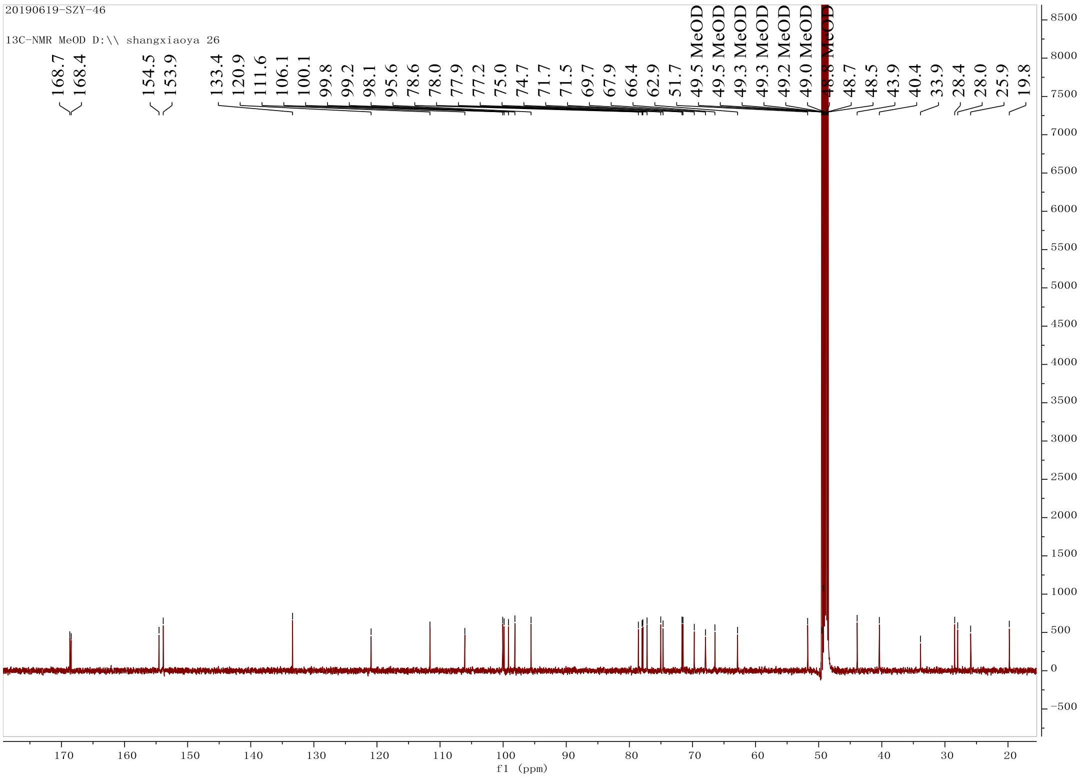

Figure S51 ^{13}C NMR spectrum of compound **7**

frontiers

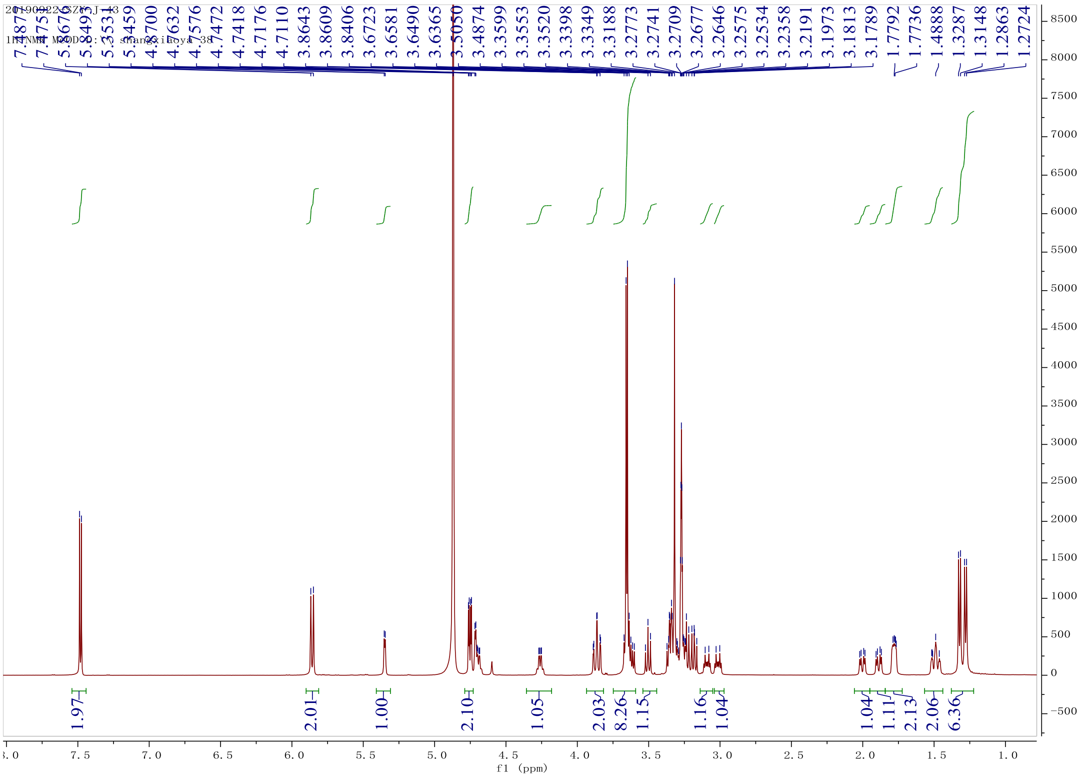

Figure S52 ^{1}H NMR spectrum of compound **8**

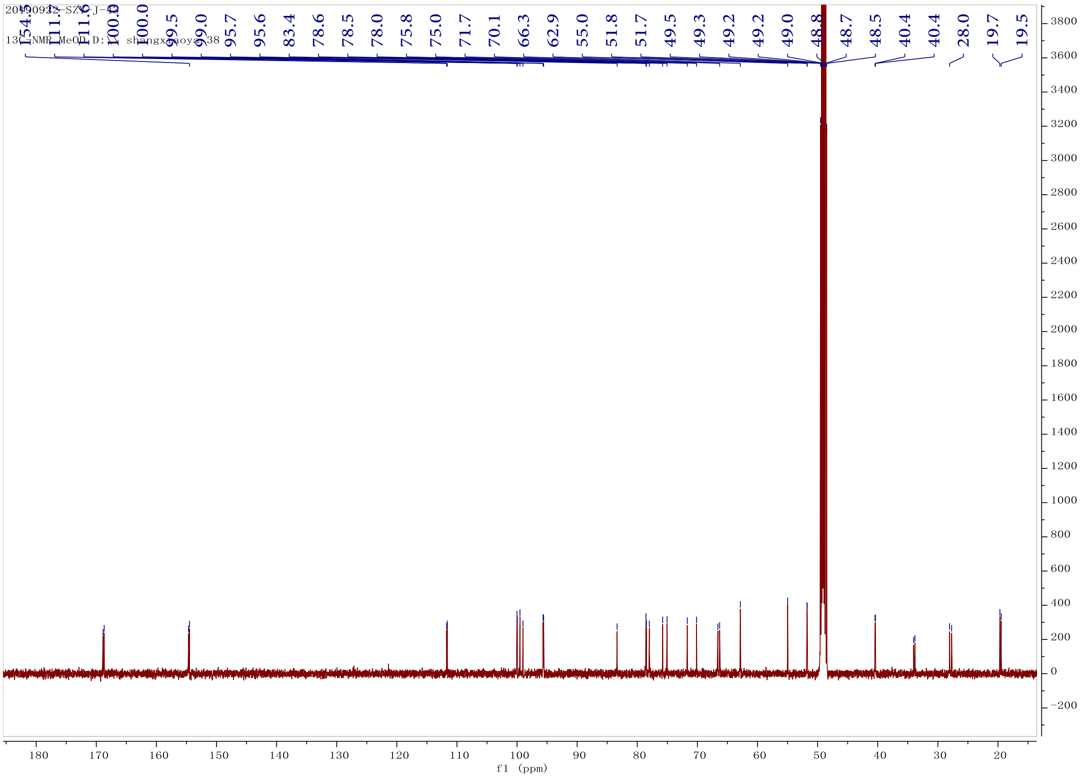

Figure S53 ^{13}C NMR spectrum of compound **8**

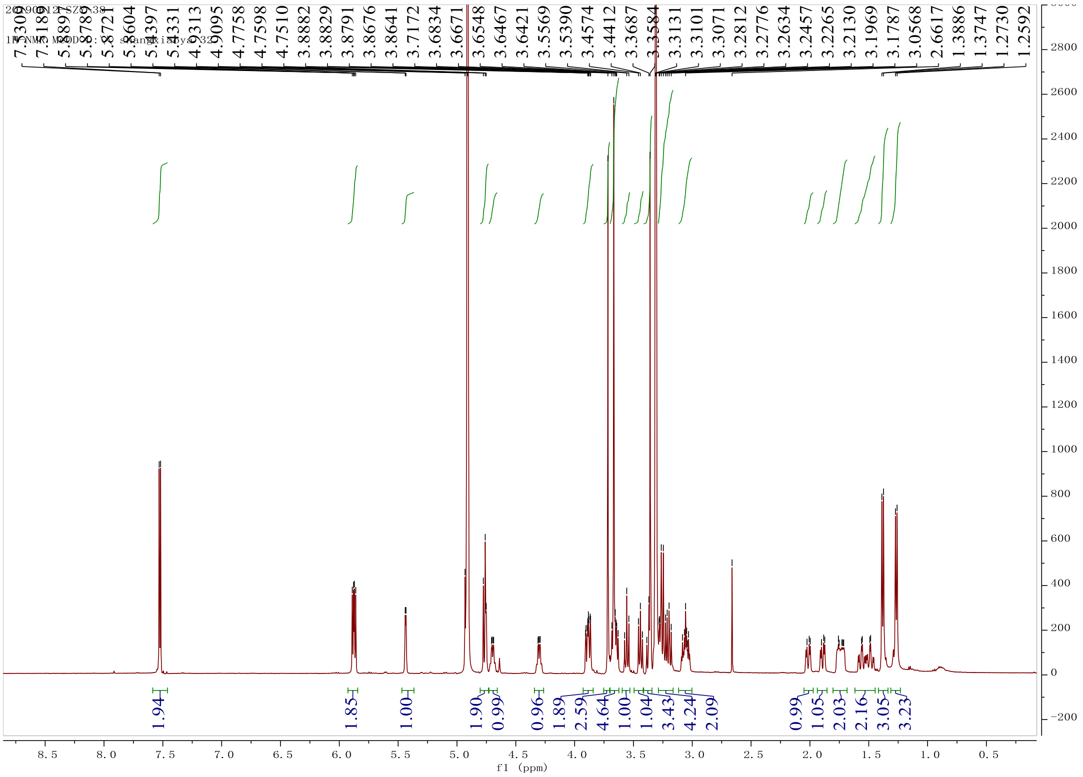

Figure S54 ^{1}H NMR spectrum of compound **9**

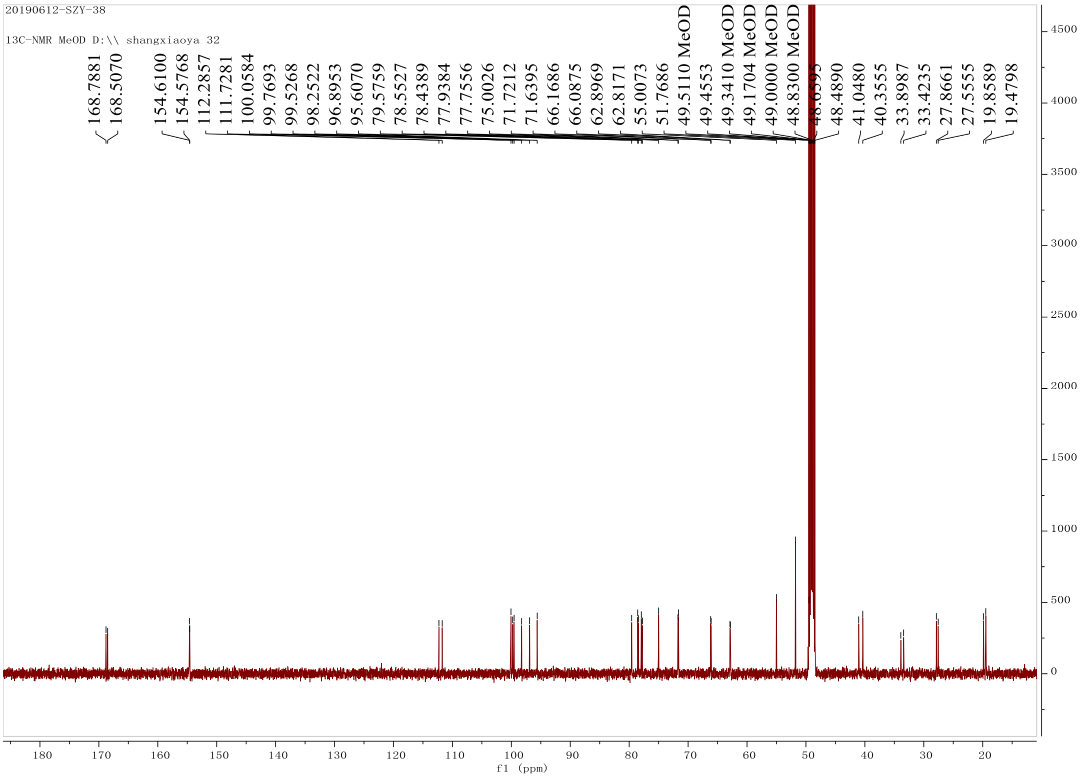

Figure S55 ^{13}C NMR spectrum of compound **9**

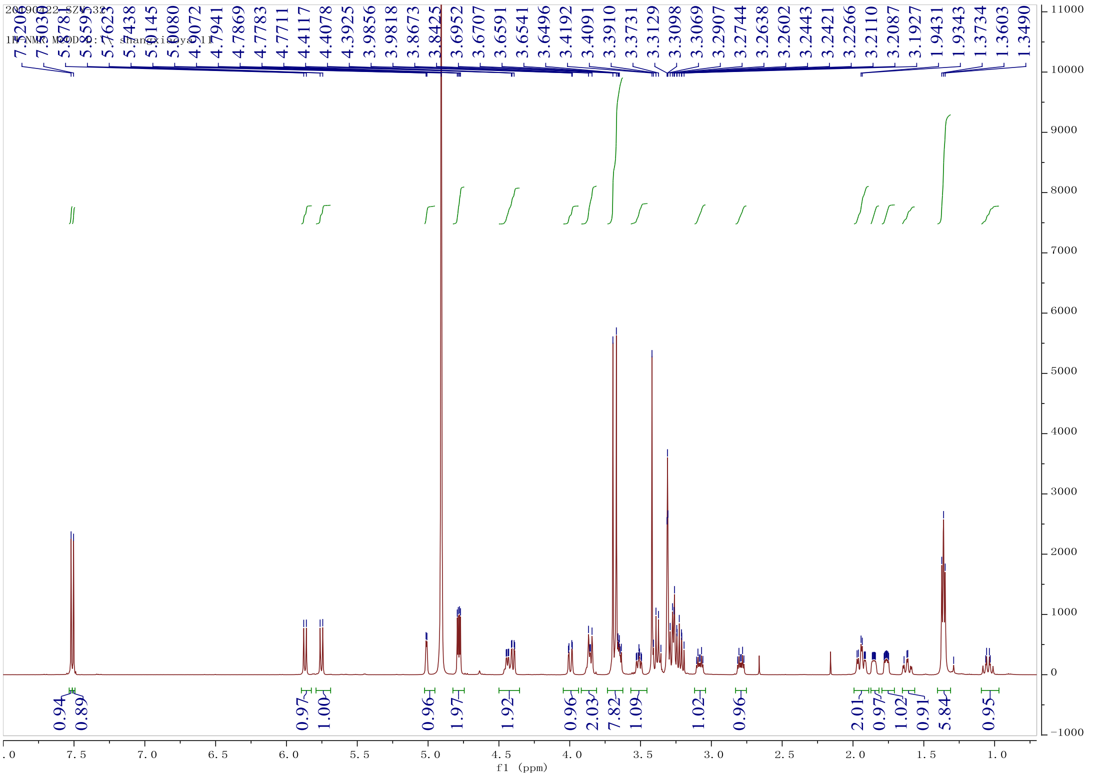

Figure S56 ^{1}H NMR spectrum of compound **10**

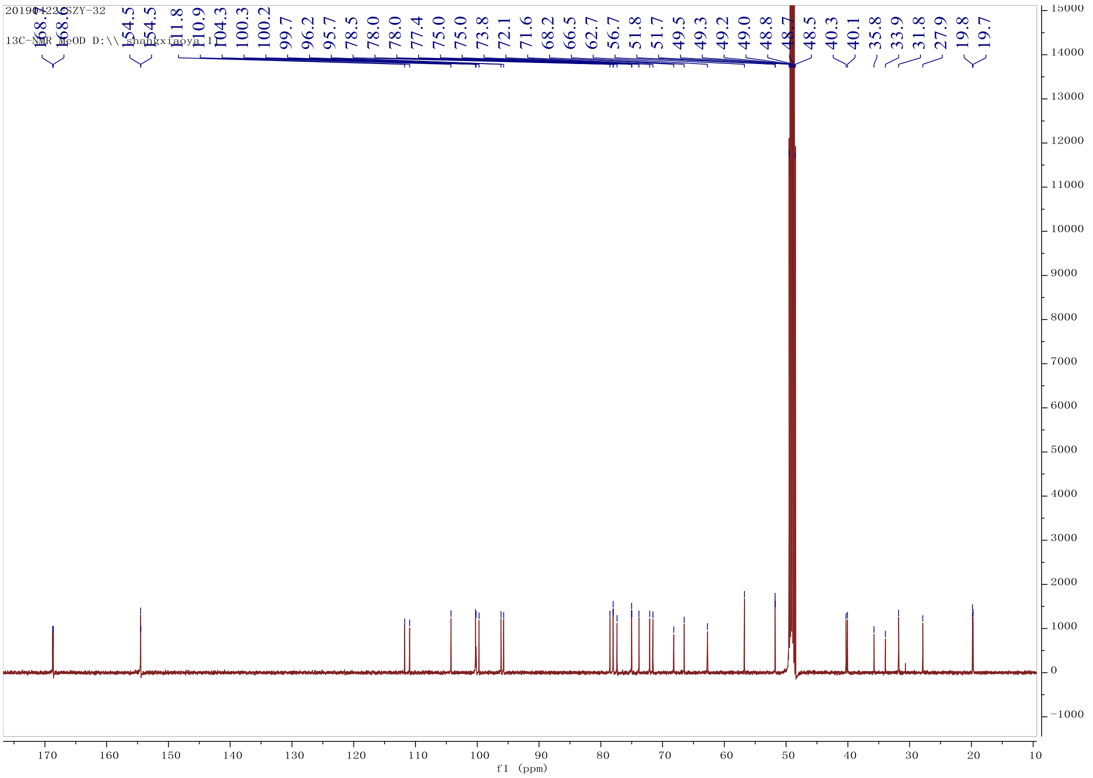

Figure S57 ^{13}C NMR spectrum of compound **10**

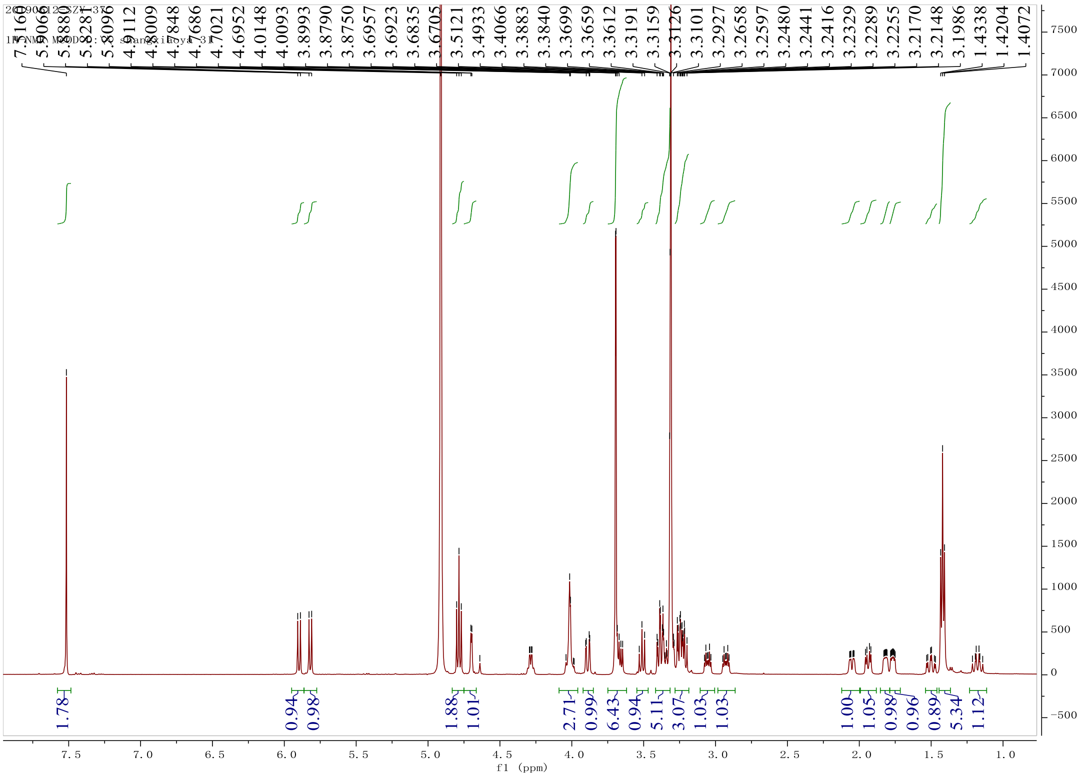

Figure S58 ^{1}H NMR spectrum of compound **11**

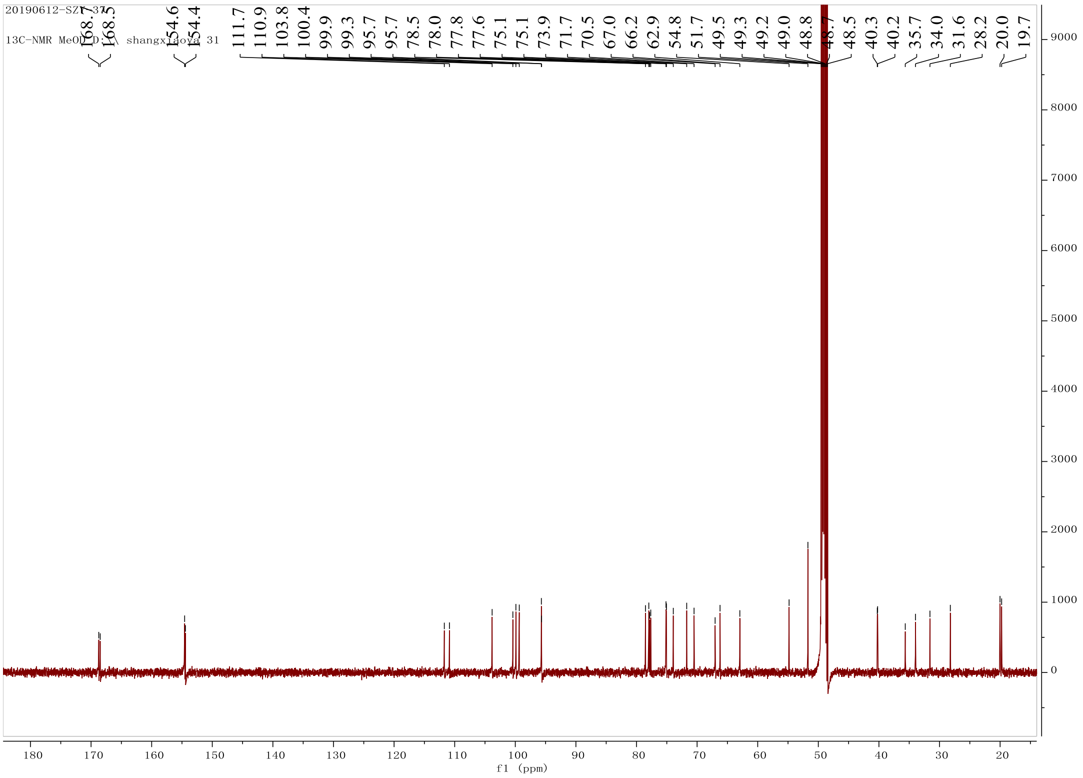

Figure S59 ^{13}C NMR spectrum of compound **11**

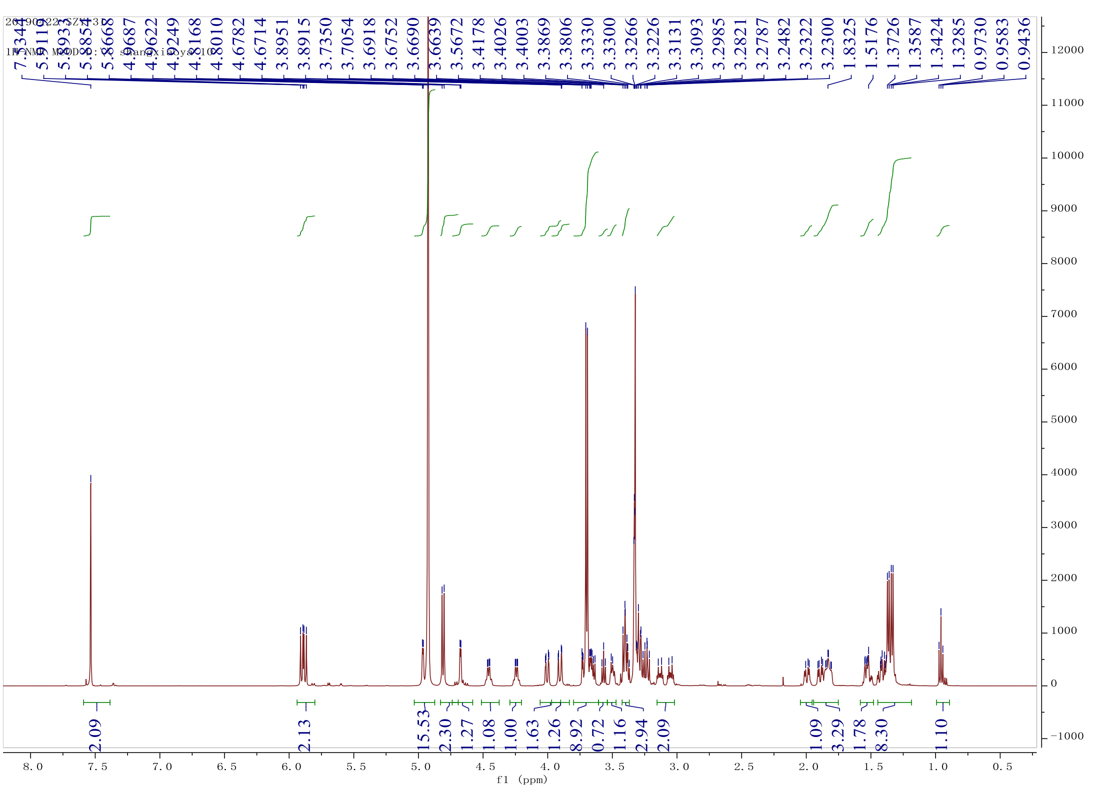

Figure S60 ^{1}H NMR spectrum of compound **12**

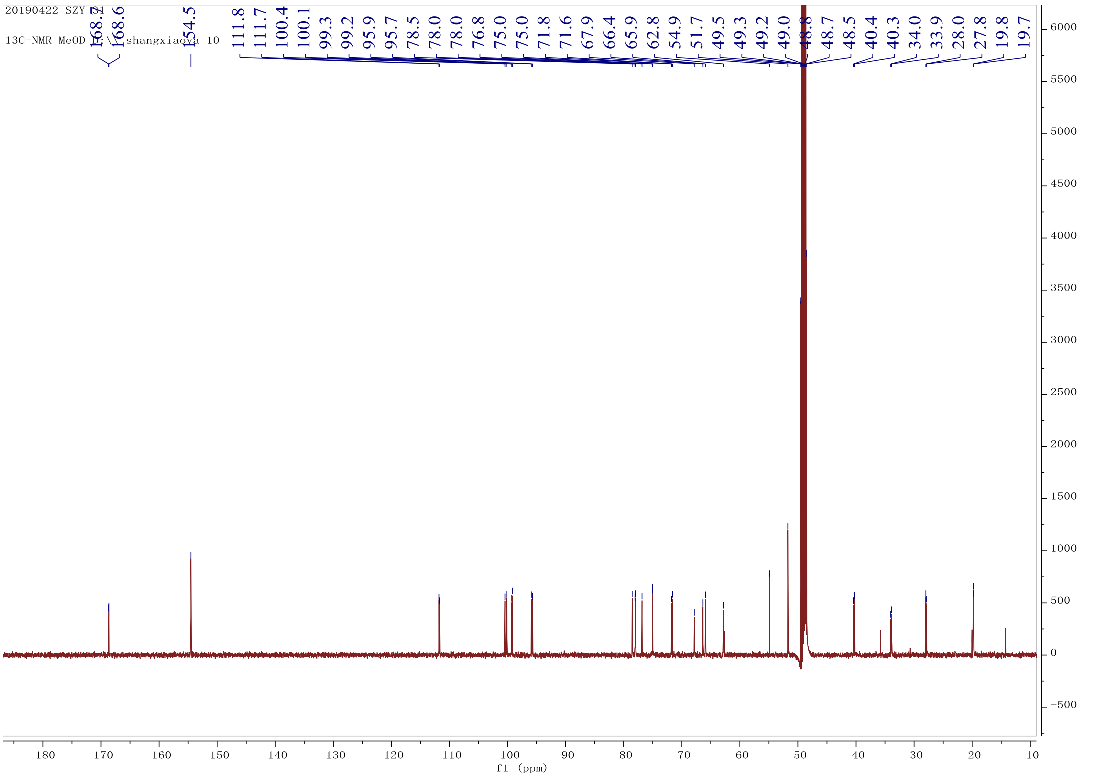

Figure S61 ^{13}C NMR spectrum of compound **12**

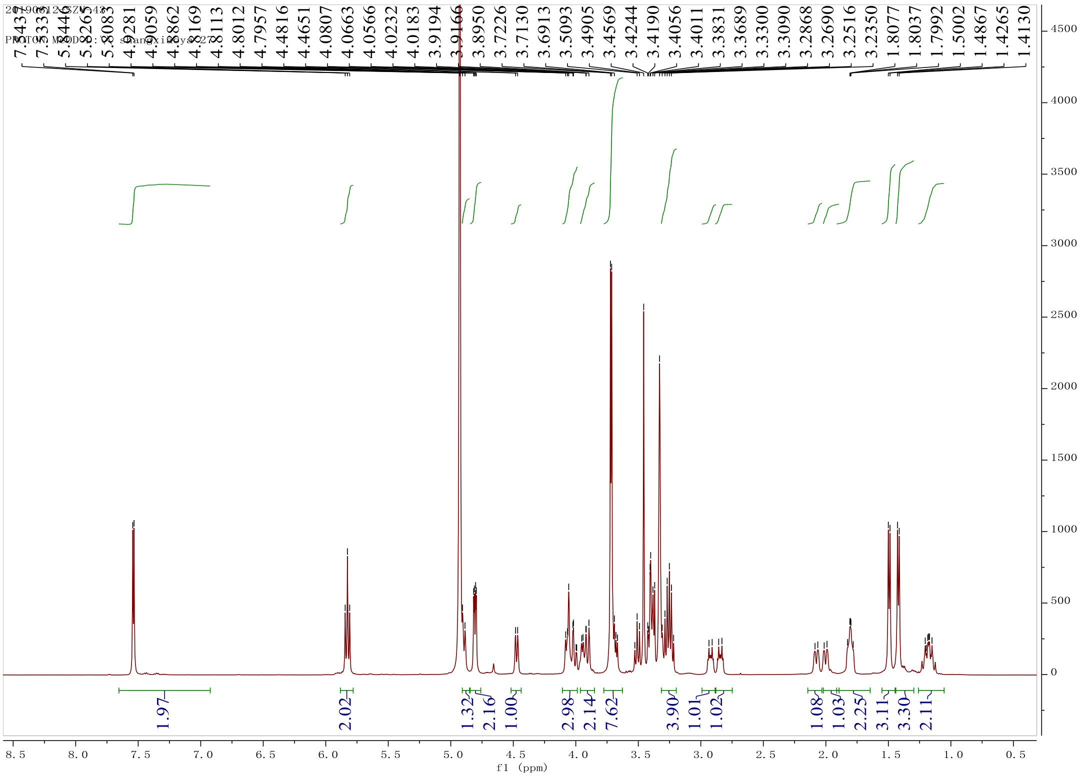

Figure S62 ^{1}H NMR spectrum of compound **13**

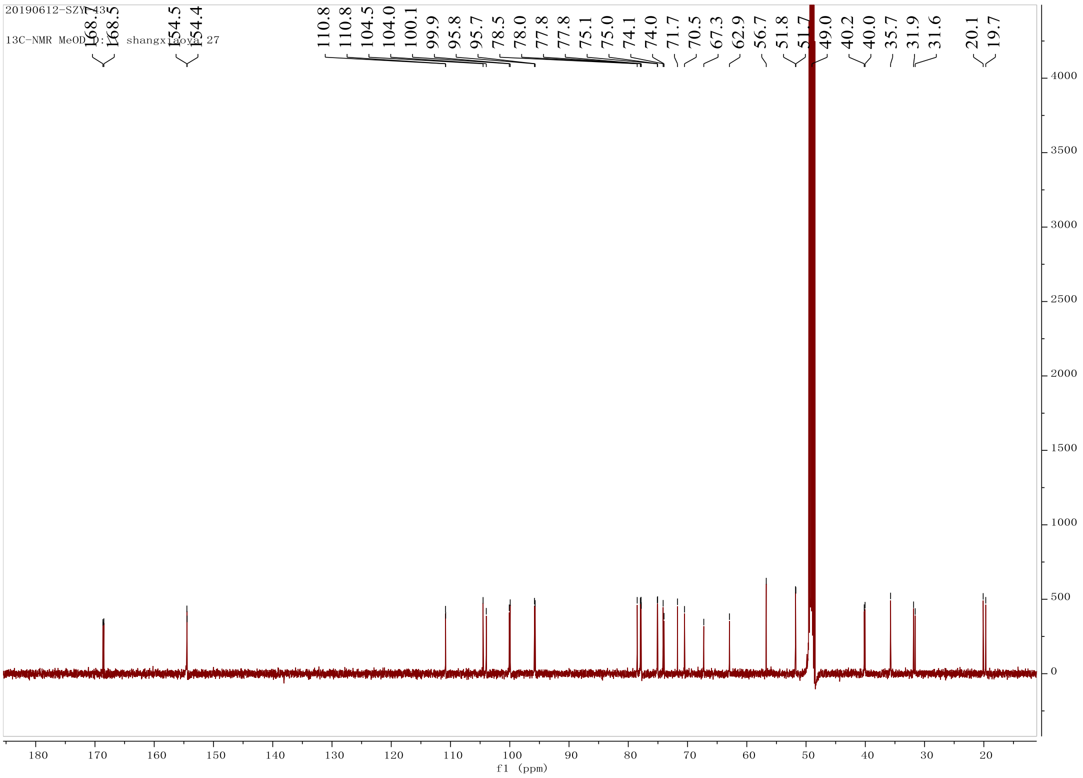

Figure S63 ^{13}C NMR spectrum of compound **13**

frontiers

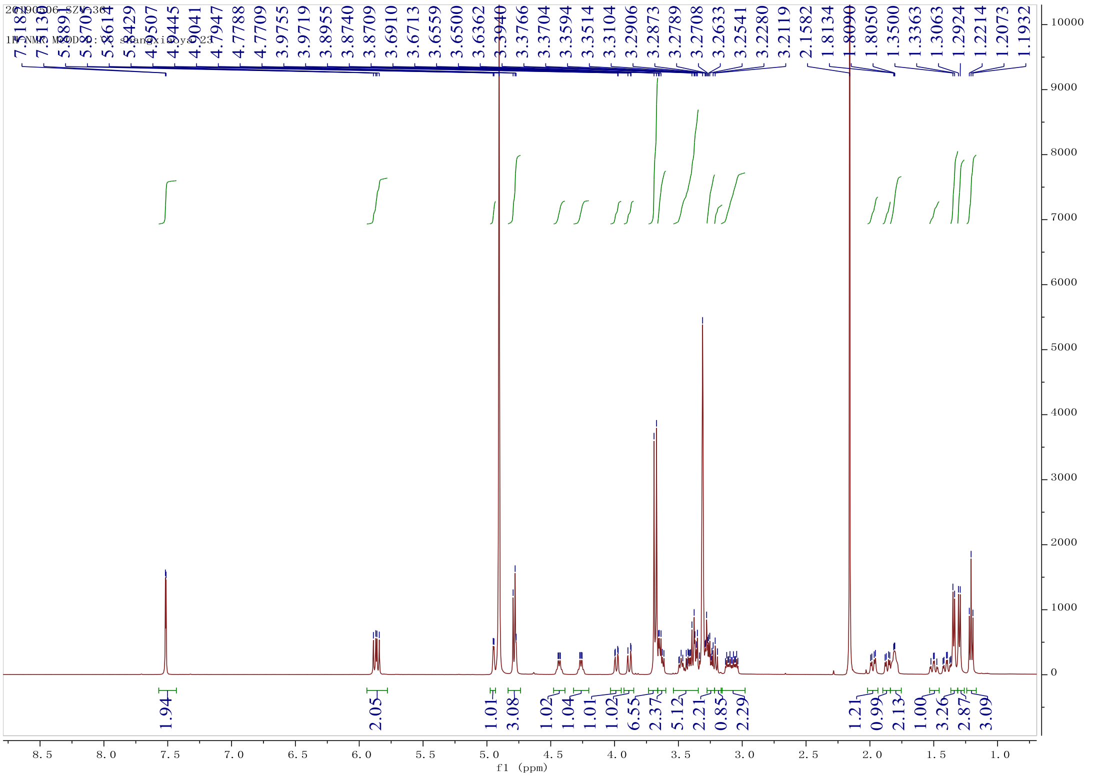

Figure S64 ^{1}H NMR spectrum of compound **14**

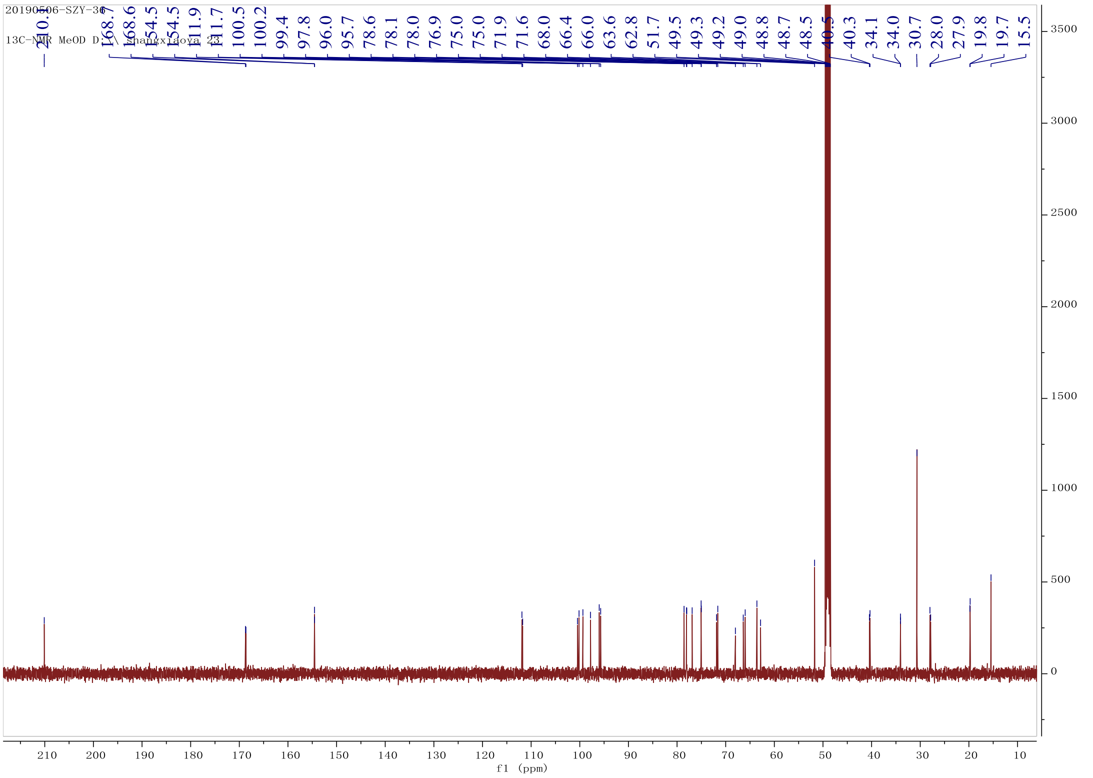

Figure S65 ^{13}C NMR spectrum of compound **14**

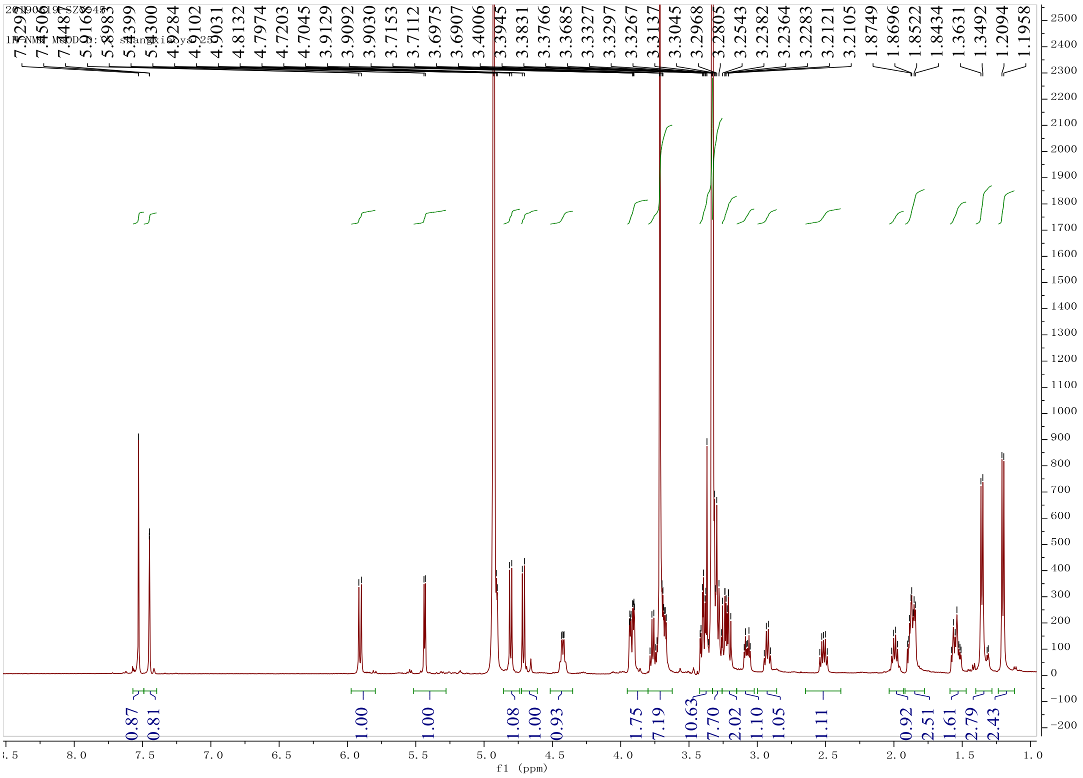

Figure S66 ^{1}H NMR spectrum of compound **15**

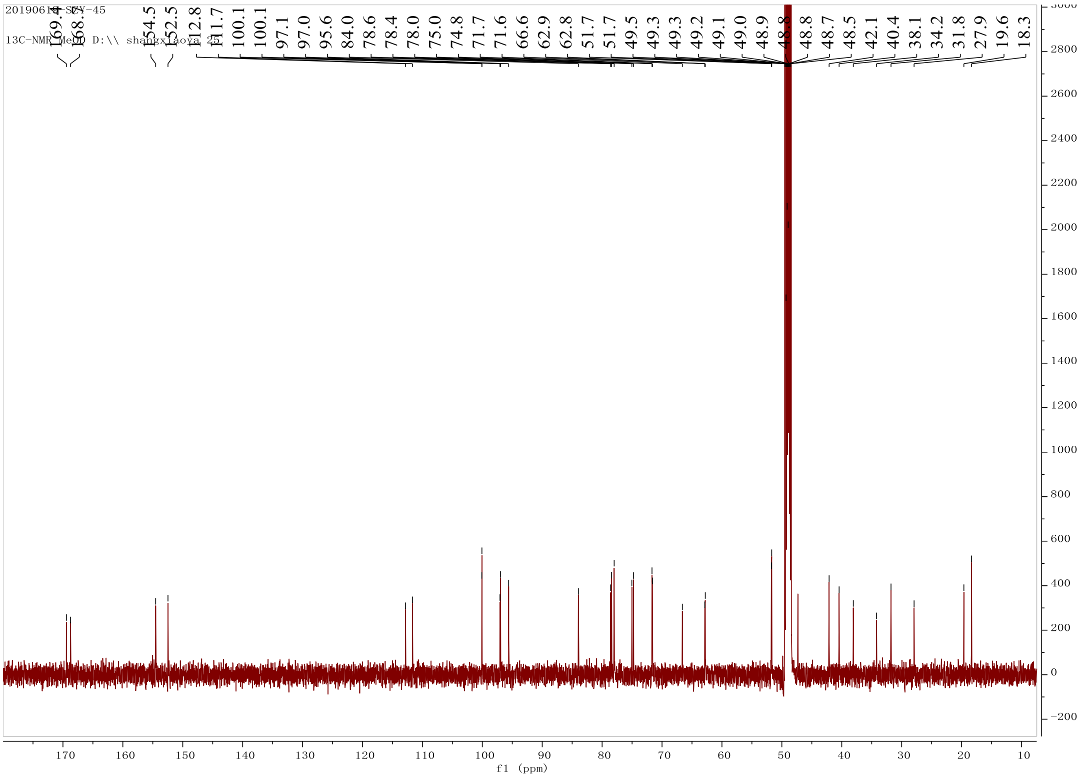

Figure S67 ^{13}C NMR spectrum of compound **15**

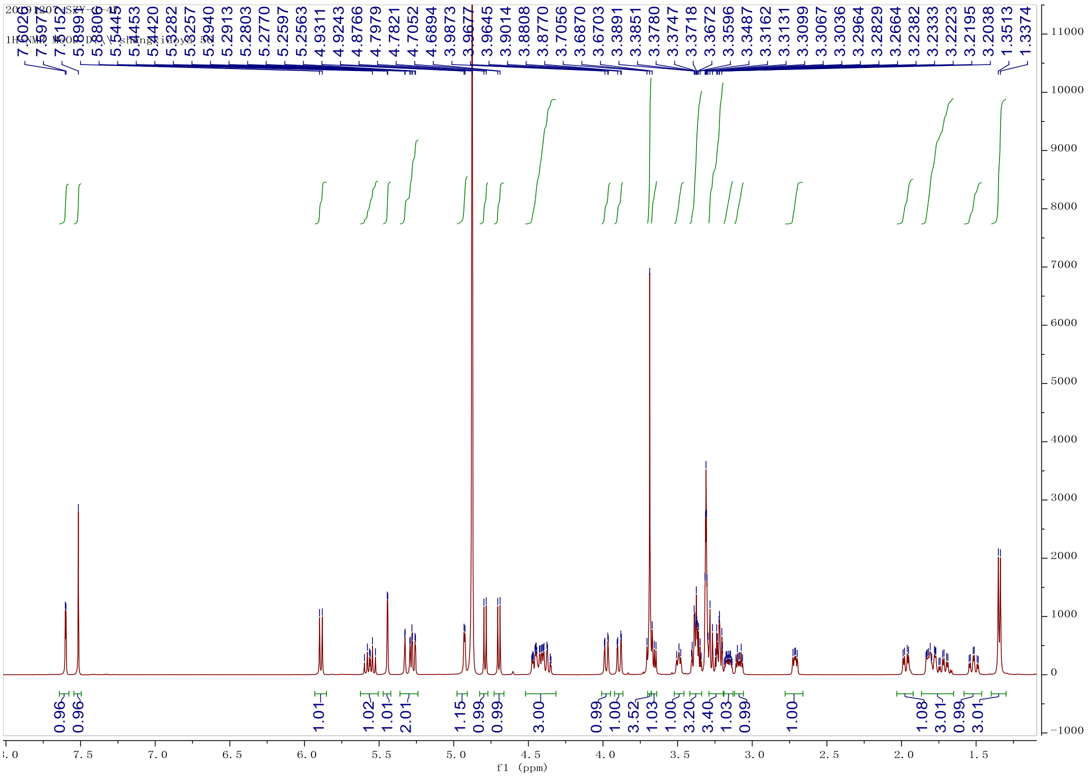

Figure S68 ^{1}H NMR spectrum of compound **16**

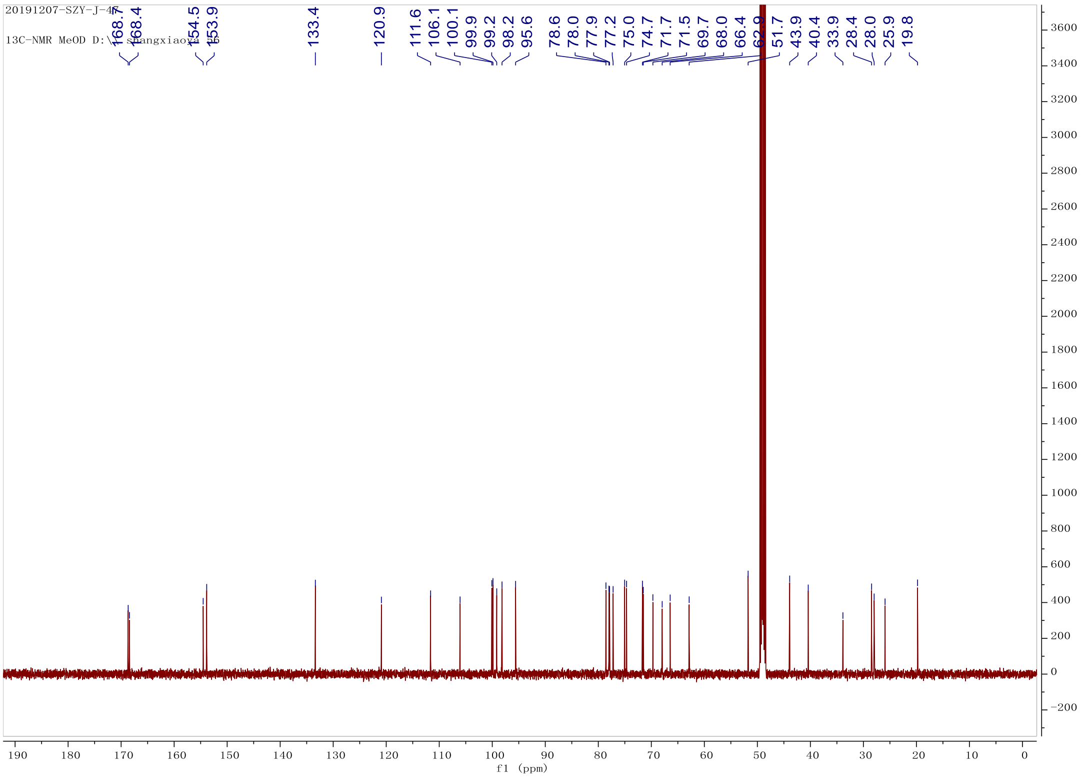

Figure S69 ^{13}C NMR spectrum of compound **16**

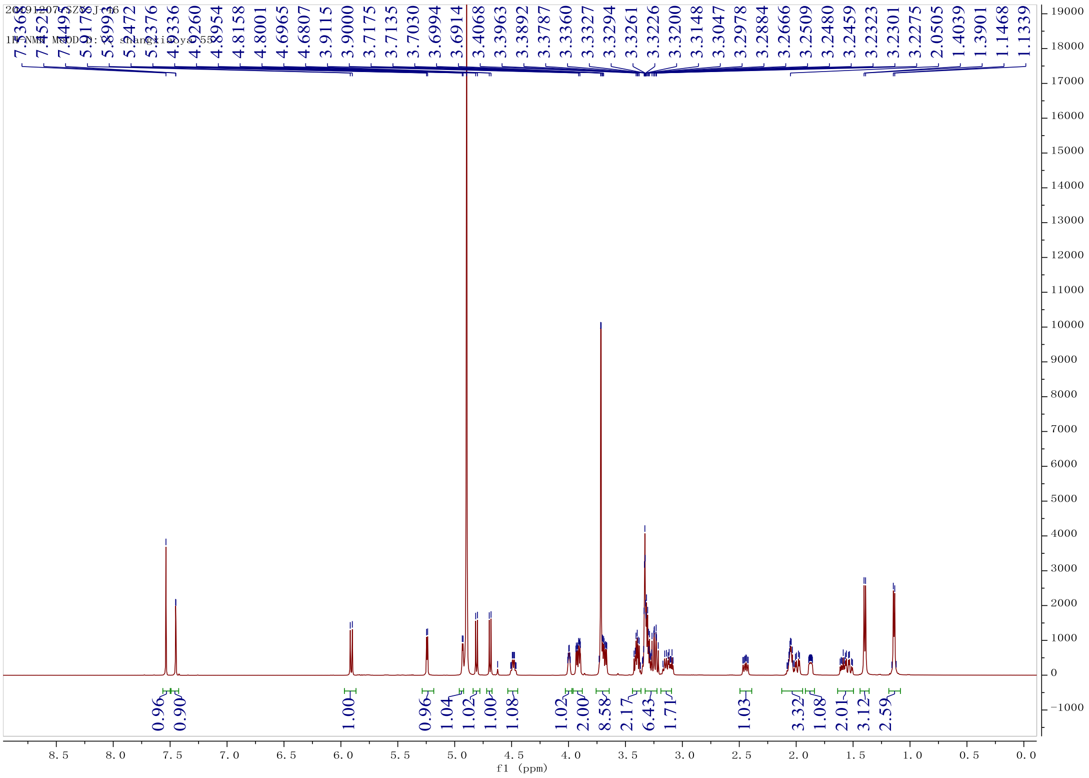

Figure S70 ^{1}H NMR spectrum of compound **17**

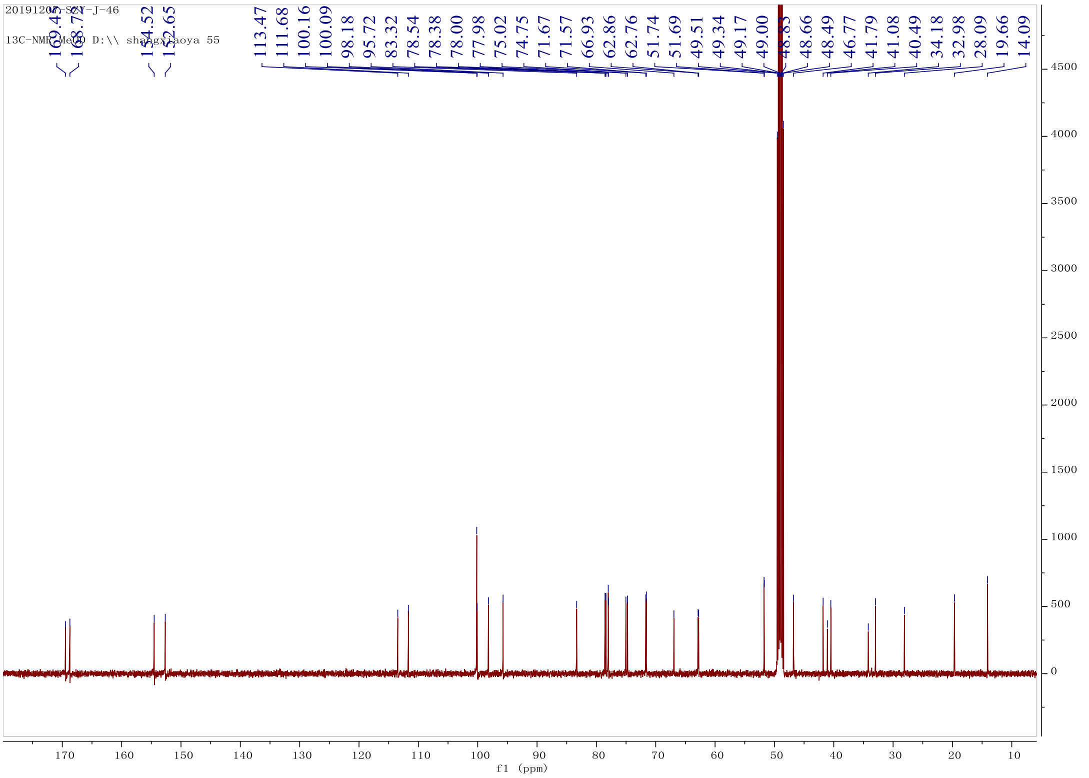

Figure S71 ^{13}C NMR spectrum of compound **17**
